# On the Itô-Stratonovich diffusion limit for the magnetic field in a 3D thin domain

**DOI:** 10.1007/s40072-026-00427-x

**Published:** 2026-05-29

**Authors:** Federico Butori, Franco Flandoli, Eliseo Luongo

**Affiliations:** 1 Scuola Normale Superiore, Piazza dei Cavalieri, 7, 56126 Pisa, Italy; 2 Fakultät für Mathematik, Universität Bielefeld, 33501 Bielefeld, Germany

**Keywords:** Thin Layer, Magnetohydrodynamics, Transport Noise, Eddy Viscosity Model, Alpha Effect, Beta Effect, 60H15, 76F25

## Abstract

We introduce a stochastic model for a passive magnetic field in a three dimensional thin domain. The velocity field, white in time and modelling phenomenologically a turbulent fluid, acts on the magnetic field as a transport-stretching noise. We prove, in a quantitative way, that, in the simultaneous scaling limit of the thickness of the thin layer and the separation of scales, the mean in the thin direction of the magnetic field is close to the solution of the equation for the magnetic field with additional dissipation. For certain choices of noises with correlations between their components, without mirror symmetry and with a non zero mean helicity, we identify, in the limit, a first-order term, in addition to the extra dissipation term. However, in the limit system this term does not produce growth of the $L^2$ norm, which is commonly referred to in the literature as the dynamo effect. Consequently, we extend a no-dynamo theorem to thin layers.

## Introduction

We consider a linear vector-valued SPDE in the unknown $B_{t}^{\varepsilon }=B_{t}^{\varepsilon }\left( x\right) \in \mathbb {R}^{3}$, $x\in \mathbb {T}_{\varepsilon }^{3}=\mathbb {T}^{2}\times \mathbb {R}/(2\varepsilon \pi \mathbb {Z})$ (a model of thin domain), $t\in \left[ 0,T\right]$, of advection type, loosely written as (see the rigorous formulation in Subsection 2.4 below)

1.1 $$\begin{aligned} {\left\{ \begin{array}{ll} \partial _{t}B_{t}^{\varepsilon } &  =\eta \Delta B_{t}^{\varepsilon }+\nabla \times \left( v_{t}^{\varepsilon }\times B_{t}^{\varepsilon }\right) \\ \operatorname {div}B_{t}^{\varepsilon } &  =0\\ B_{t}^{\varepsilon }|_{t=0} &  =B_{0}^{\varepsilon } \end{array}\right. } \end{aligned}$$

where $v_{t}^{\varepsilon }=v_{t}^{\varepsilon }\left( x\right) \in \mathbb {R}^{3}$ is a white noise in time, with suitable space covariance structure, described in detail in subsection 2.3. The multiplications $v_{t}^{\varepsilon }\cdot \nabla B_{t}^{\varepsilon }$ and $B_{t}^{\varepsilon }\cdot \nabla v_{t}^{\varepsilon }$ appearing in the development of the term $\nabla \times \left( v_{t}^{\varepsilon }\times B_{t}^{\varepsilon }\right)$ are understood in Stratonovich sense.

Our main contribution is proving that the vertical average of the magnetic field, $\overline{B^{\varepsilon }_{t}}=\frac{1}{2\pi \varepsilon }\int _0^{2\pi \varepsilon } B^{\varepsilon }_t(x_1,x_2,x_3)dx_{3}$, converges in a suitable distributional topology to the solution $\overline{B}_{t}$ of the system

1.2 $$\begin{aligned} {\left\{ \begin{array}{ll} \partial _{t}\overline{B}_{t} &  =\left( \eta +\eta _{T}\right) \Delta \overline{B}_{t}+\nabla \times \left( \mathcal {A}\overline{B} _{t}\right) \\ \operatorname {div}\overline{B}_{t} &  =0\\ \overline{B}_{t}|_{t=0} &  =\overline{B}_{0} \end{array}\right. } \end{aligned}$$

where $\overline{B}_{0}$ is the limit of $\frac{1}{2\pi \varepsilon }\int _0^{2\pi \varepsilon } B^{\varepsilon }_0(x_1,x_2,x_3)dx_{3}$. Here $\eta _{T}$ is an “eddy magnetic diffusivity”, linked to the space-covariance of $v_{t}^{\varepsilon }\left( x\right)$; the additional term $\eta _{T}\Delta \overline{B}_{t}$ is often called the beta-term in the literature devoted to equations for the magnetic field driven by a turbulent velocity field (see subsection 1.1 below). $\mathcal {A}$ is a matrix linked to the mean helicity of the noise; the term $\nabla \times \left( \mathcal {A}\overline{B}_{t}\right)$ is usually called the alpha-term. The convergence result is stated in detail in Section 2.4, see in particular Theorem 2.15, and the formulation given above is reconstructed in Appendix B.

We classify such a theorem as an *Itô-Stratonovich stochastic diffusion limit*, in analogy with the deterministic and stochastic theory of diffusion limits based on homogenization techniques (see for instance [16, 19]). The approach however is different and is strongly based on the Itô-Stratonovich corrector, initiated by [14] (see also [12] and references therein), from which we derive this terminology. We give a rough idea of how this technique works. The Itô formulation of equation (1.1) involves a linear, second-order corrector term that is roughly obtained by squaring the operators $\mathcal {L}_{v^\varepsilon }\cdot := \nabla \times (v^\varepsilon \times \cdot )$. We refer to Section 2.4 for the detailed computations and simply denote by $\mathcal {D}^{\varepsilon }$ the additional differential operator arising from the Itô-Stratonovich corrector. We interpret this operator as a *mean field* term (as opposed to the *fluctuations* represented by the Itô integrals), see also subsection 1.1 below. It can be shaped in various ways, through appropriate choices of the noise covariance, so that it admits a non-trivial limit as $\varepsilon \rightarrow 0$. In particular, it can be chosen so that

$$\begin{aligned} \mathcal {D}^{\varepsilon }\cdot \rightarrow \eta _T\Delta \cdot +\nabla (\mathcal {A}\times \cdot )\quad \text {as }\varepsilon \rightarrow 0, \end{aligned}$$

cf. (1.2). Owing to the linearity of the equation, this easily yields

$$\begin{aligned} \mathbb {E}\left[ \overline{B^{\varepsilon }_{t}}\right] \rightarrow \overline{B}_t\quad \text {as }\varepsilon \rightarrow 0. \end{aligned}$$

However, the convergence $\overline{B^{\varepsilon }_{t}}\rightarrow \overline{B}_t$ requires, additionally, a careful control of the fluctuations induced by the stochastic integrals. Ultimately, this issue is linked to the derivation of bounds, uniform in ε, in sufficiently strong norms of $\overline{B^{\varepsilon }_{t}}$. In the case of transport noise, cf. [14], this is trivial due to its energy-preserving nature. In our setting, however, energy preservation is broken by the random stretching term $B^\varepsilon _t\cdot \nabla v^\varepsilon _t,$ which has increasingly strong effects as $\varepsilon \rightarrow 0$. This behaviour is caused by the assumptions on $v^\varepsilon$ required to retain a non-trivial limiting mean-field operator; see, e.g., [6, Appendix 2]. We refer to Section 2.5 for a more in-depth discussion of the difficulties in obtaining uniform controls in the presence of transport–stretching noise, as well as for a presentation of our strategy to address this problem in the framework of magnetic fields in a thin domain.

In this context, our main result, Theorem 2.15, represents an important technical advancement in the study of these kinds of diffusion limits for three dimensional fluids. Our model lies between the full three-dimensional regime and previously considered toy models, while retaining physical relevance, as explained in subsection 1.1. The closest previous result in this direction is [9], a 2D-3C (two-dimensional, three component) model, where a number of ideas used also here have been already introduced. However, the thin domain treated here is a truly 3D-3C model, which reduces to 2D-3C only in the limit. The control of the limit is demanding, as already mentioned, due to the random stretching term. Without it, as in [8], the control of the limit is easier, while the 2D-3C version of this system of [9], being 2D from the beginning, eliminates part of the difficulty.

### Comments on the physical motivation

The model introduced above for $B_{t}^{\varepsilon }$ is a classical model for a magnetic field driven by a velocity field $v_{t}^{\varepsilon }$, with some degree of resistivity (namely closeness to ideal) given by η. We refer to the classical monographs [21, 22] for a detailed discussion on the topics highlighted in this section. This model is used in Plasma Physics to describe several phenomena, the one of most interest for us being the dynamo effect, that is the capability of a magnetohydrodynamic system to self-sustain, against resistive dissipation. Let us comment about the model and result under the usual view of dynamo research.

The step from the equation for $B_{t}^{\varepsilon }$ to a “mean field" equation like the one above for $\overline{B}_{t}$ is a classical step in the Physics literature on this topic. The usual procedure consists in taking averages of $B_{t}^{\varepsilon }$, getting a Reynolds type tensor which mixes bilinearly the randomness of $B_{t}^{\varepsilon }$ and $v_{t} ^{\varepsilon }$, and close the equation for the average, large scales components, of $B_{t} ^{\varepsilon }$ by suitable closure assumptions on the average of the bilinear term. Compared to these derivations, we do not aim to get new final mean field equations, since those indicated in Plasma Physics literature are already deep and compared with reality; our aim is to justify them. Indeed, their validity is far from trivial, presumably false in full generality, for substantial reasons and not just for a question of mathematical rigour. Let us explain why.

As already mentioned, in our approach, the Itô-Stratonovich corrector appearing when we rewrite the term $\nabla \times \left( v_{t}^{\varepsilon }\times B_{t}^{\varepsilon }\right)$ from the Stratonovich form to the Itô one, corresponds precisely to the mean field term (or sum of terms) discovered in the literature under the most natural closure formulae. But in our rigorous derivation there is still the Itô term, which should disappear in the limit when $\varepsilon \rightarrow 0$. There is no obvious reason why it should disappear, and in general it contains fluctuations which may be very important and alter the limit result. In scalar and 2D models, where stretching of vector quantities does not appear, it is possible to prove that the Itô term is negligible in the limit (see for instance [3, 6, 7, 10, 12–14] ). In 3D, the problem is extremely difficult. We start to throw some light with this work in a truly 3D model, thanks to the asymptotic simplifications of the thin domain; previously some basic ingredients have been understood for the 2D-3C model of [9].

Therefore, to summarize the previous discussion, our main contribution is to provide a framework in which to make rigorous the mean field theory of a magnetic field $B_{t}^{\varepsilon }$ advected by a turbulent velocity field $v_{t}^{\varepsilon }$.

A comment about the physical meaning of our mean field equation is now in order.

The final mean field system for $\overline{B}_{t}$ contains two new terms, the alpha and beta terms. Let us start from the beta term $\eta _{T}\Delta \overline{B}_{t}$. Let us recall the following sentence from Krause e Rädler [17, Page 12]: “homogeneous isotropic mirror symmetric turbulence only influences the decay rate of the mean magnetic fields, which is enhanced in almost all cases of physical interest”. When the field $v_{t}^{\varepsilon }$ is mirror symmetric, the average helicity of $v_{t}^{\varepsilon }$ (defined in Appendix B below in the case of our white noise field $v_{t}^{\varepsilon }$) is zero, which then implies that $\mathcal {A}=0$, namely the alpha-term $\nabla \times \left( \mathcal {A} \overline{B}_{t}\right)$ is absent. In this case, the only mean-field effect of the turbulent field $v_{t}^{\varepsilon }$ is to produce the beta-term $\eta _{T}\Delta \overline{B}_{t}$, which enhances the decay rate, as stated by [17, Page 12]. Therefore our result rigorously confirms that prediction, in the particular case of thin domains.

Concerning the other additional term, namely the alpha-term $\nabla \times \left( \mathcal {A}\overline{B}_{t}\right)$, the story is more complicated. In fully 3D cases such a term may be responsible for the growth of the magnetic field by stretching and it is advocated as one of the sources of dynamo, a very important observed phenomenon which is related to the growth or maintenance of the magnetic field in certain planets and stars. However, in our case the term $\nabla \times \left( \mathcal {A}\overline{B}_{t}\right)$, although present, cannot produce an increase of the magnetic field. This is due to the specific algebraic structure, described in Section 2.4. The matrix $\mathcal {A}$ is degenerate and propagates only certain information of $\overline{B}_{t}$ (those coming from the vertical part of the vector potential) which do not create a loop suitable for increase (the vertical part of the vector potential may only dissipate). We refer to Remark 2.18 below for a better understanding of the last sentence.

To understand this discrepancy, let us point out that in our model there are two sources of high frequency perturbations that affect the mean field large scales magnetic field $\overline{B}_t$. One is given by the turbulent velocity field $v^\varepsilon$, the noise part, while the other is given by the geometry of the layer, which provokes high frequency oscillations in the vertical (thin) direction. The averaging of the noise part is taken care by the probabilistic part of our argument, which is the core of our proof, while the vertical small scales are filtered out by taking averages in the vertical direction. This step allows us to deal with an effective 2D-3C quantity. However, it does not make the problem simply 2D-3C as in previous models, due to additional Reynolds-type tensors, stemming from the bilinear terms, which couple the fast oscillating quantities with the large scale averaged quantities which we have to handle carefully. We refer to Section 2.5 for details.

As it is well known, Cowling’s theorem states that a 2D-3C conducting plasma cannot give rise to a dynamo, due to geometric constraints, see for instance [15, Section 3.5]. However, in almost all examples of astrophysical plasmas (such as rotating galaxies), whose motion is confined to an *almost* two-dimensional plane, large scale magnetic fields are in fact observed. Their very existence, as well as their persistence, is believed to be connected to dynamo effects (see [1]). This means that small tridimensional effects cannot be neglected when modelling such systems. It follows that 2D-3C models necessarily fail to capture the right physics. As a consequence of our results, we rigorously extend this constraint by showing that not only 2D-3C models lack such important features, but also a fully 3D stochastic model of a thin domain, whose dimension is reduced *only* by averaging out the vertical oscillations (thus retaining the effects of the coupling terms mentioned above, which are completely neglected in 2D-3C models), fails to capture the relevant physical phenomena, thus corroborating with a rigorous result the principle for which such systems must be treated in their full 3D complexity. We also mention that the case of an isotropic 3D domain has been studied by the first and third named authors in [2], which appeared after the first version of the present manuscript. That work provides an example in which a turbulent stochastic fluid $v^{\varepsilon }$, similar to the one considered here (cf. Hypothesis 2.6 and [2, Hypothesis 2.5]), and the induced alpha term are capable of sustaining the magnetic field. From a modelling perspective, the results presented there can be seen as complementary to those discussed here. Both support the conclusion that averaging out vertical oscillations may lead to a loss of crucial physical mechanisms, and that magnetohydrodynamic systems must be addressed in their full 3D complexity. Specifically, by the result of the present work we can conclude that if a dynamo is active in our model, then it must necessarily act on the high frequency modes $(B^\varepsilon )' = B^\varepsilon - \overline{B^\varepsilon }$ (the one neglected by the averaging procedure), a conclusion which is compatible and corroborated by the results in [2].

### Comparison with the literature

The dynamo problem for the linear equation (1.1), also called kinematic dynamo, has been recently addressed in various forms in the mathematical literature. In this section we briefly mention those we believe are the most relevant. The classical formalization of the dynamo problem in mathematical terms is extensively discussed in the seminal reference [4], where it is stated in terms of Lyapunov exponents, and where also the concepts of slow and fast dynamos are introduced. Loosely speaking, the problem asks to construct a flow *u*, deterministic or random, for which the solution of the induction equation grows exponentially in the $L^2$ norm, on any time interval. Then, the dynamo is called *fast* if the exponential growth rate stays positive in the limit of zero resistivity. On this topic, see also the classical work [26]. The problem stated in this way does not require directly the derivation of any mean field model, and indeed it was recently approached, in the ideal η=0 case, in [33], by means of the positivity of the top Lyapunov exponent of the Lagrangian trajectories of the (randomized) underlying flow *u*; and later solved, in the much more difficult resistive case (η>0), in [34] using tools from perturbation theory, providing also the first rigorous example of a fast dynamo on $\mathbb {R}^3$, induced by an autonomous flow. We also mention the work [27], where the author proves existence of *almost* fast dynamos in $\mathbb {T}^3$ using a controllability argument to construct an ad hoc time-dependent flow. Finally, in the stochastic setting (i.e. when the flow is given by a white in time stochastic process), the first and third named author, subsequently to the appearance of the first version of this paper, showed in [2] that, in the full 3D setting, a helical stochastic flow can induce a dynamo, in a looser sense than that in [4], by proving a mean field-type result akin to the one proved here, but crucially, with enough richness so to sustain a dynamo. Beyond the dynamo problem, the derivation of mean field models has an independent interest, for which we refer to the monograph [35], where the authors derive several mean-field models using homogenization-type tools. In the present work, the spirit is more akin to this second line of research, however going in the opposite direction of the dynamo problem, by providing, as explained in the previous section, a novel result of *no-dynamo* type. Finally, in comparison with our subsequent work [2], it is worth noticing that here, thanks to the possibility of exploiting a regularizing effect of the special thin geometry, we are able to relax the assumptions on the noise, allowing a slower decay rate for the noise coefficients (i.e. stronger fluctuations) (*cf.* Hypothesis 2.6 and [2, Hypothesis 2.5]). Moreover, from the technical point of view, in the present work several new ideas are needed to deal with the scaling of the equation *and* of the domain at the same time, and to extract a regularizing effect of the thin domain (*cf.* Section 2.5).

### Overview

The content of the paper is the following one. In Section 2 we introduce rigorously our framework. In particular, we define the structure of the noise, $v^{\varepsilon }_t$, in subsection 2.3, while we state our main result in Section 2.4 and describe the main ideas behind its proof in Section 2.5. The remainder of the paper is dedicated to proving our main result. In Section 3 we prove estimates for $B^{\varepsilon }_t$ uniformly in the thickness of the domain, then we show the convergence of $\overline{B^{\varepsilon }_{t}}$ to $\overline{B}_t$ in Section 4 . We conclude the paper with two appendices. In Appendix A we provide an explicit representation of the Itô-Stratonovich corrector in (1.1). In Appendix B we compute an average of the helicity of $v^{\varepsilon }_t$, highlighting its connection to the alpha-term in (1.2).

## Functional setting and main results

In subsection 2.1 we introduce the notation we follow along the paper. In Subsection 2.2 we introduce some functional analysis tools we employ in order to carry on rigorously our analysis described in Section 1. We describe the noise we employ in subsection 2.3 providing some insights in the meaning of the assumptions and comparisons with the existing literature. We state our main result in Section 2.4 and describe the main ideas behind its proof in Section 2.5 for the convenience of the reader.

### Notation

In the following we denote by $\mathbb {T}^2=\mathbb {R}^2/(2\pi \mathbb {Z})^2$ (resp. $\mathbb {T}^3=\mathbb {R}^2/(2\pi \mathbb {Z})^3$) the two (resp. three) dimensional torus. Moreover, for each ε>0 let us denote by

$$\begin{aligned} \mathbb {T}^3_\varepsilon =\mathbb {T}^2\times \mathbb {R}/(2\varepsilon \pi \mathbb {Z}) \end{aligned}$$

the thin layer three dimensional torus. In Subsection 2.2 we focus on function spaces on periodic domains which we in general denote by $\mathcal {D}$ varying between $\mathbb {T}^2,\ \mathbb {T}^3,\ \mathbb {T}^3_{\varepsilon }$.

Similarly we denote by $\mathbb {Z}^3_0=\mathbb {Z}^3\smallsetminus \{0\}$ (resp. $\mathbb {Z}^2_0=\mathbb {Z}^2\smallsetminus \{0\}$) the three (resp. two) dimensional lattice made by points with integer coordinates. Moreover, for each ε>0 let us denote by

$$\begin{aligned} \mathbb {Z}^{3,\varepsilon ^{-1}}_0=\mathbb {Z}^2\times \varepsilon ^{-1}\mathbb {Z}\smallsetminus \{0\} \end{aligned}$$

the thin layer three dimensional lattice. We introduce a partition of $\mathbb {Z}^{3,\varepsilon ^{-1}}_0=\Gamma ^{\varepsilon }_+\cup \Gamma ^{\varepsilon }_-$ such that

$$\begin{aligned}&\Gamma ^{\varepsilon }_+ =\{k\in \mathbb {Z}^{3,\varepsilon ^{-1}}_0: k_3>0 \vee (k_3=0 \wedge k_2>0) \vee (k_3=k_2=0 \wedge k_1>0)\},\\&\Gamma ^{\varepsilon }_{-}=-\Gamma ^{\varepsilon }_+. \end{aligned}$$

If $v=(v_1,v_2,v_3)^t:\mathcal {D}\rightarrow \mathbb {R}^3$ we will sometimes denote by $Dv=(\nabla v)^t$, $v_H=(v_1,v_2)^t$ in the following.

For each $j\ge 0,\ l>1$ we denote by

$$\begin{aligned}&\zeta ^N_{H,j}= N^{j-2}\sum _{\begin{array}{c} k_H\in \mathbb {Z}^2_0\\ N\le |k_H|\le 2N \end{array}}\frac{1}{|k_H|^j},\\&\zeta _{H,j}=\int _{1\le |x|\le 2}\frac{dx_1dx_2}{|x|^j}=2\pi {\left\{ \begin{array}{ll} \log 2 &  \text { if } j=2,\\ \frac{2^{2-j}-1}{2-j} &  \text { if } j\ne 2 \end{array}\right. } , \quad \zeta _{l}=\frac{1}{2}\sum _{k\in \mathbb {Z}\smallsetminus \{0\} }\frac{1}{|k|^l}<+\infty . \end{aligned}$$

In particular, by the approximation properties of Riemann sums, for each j≥0 it holds

$$\begin{aligned} \zeta _{H,j}^N= \zeta _{H,j}N^{2-j}+O(N^{1-j}). \end{aligned}$$

For two vector fields *X* and *Y* , we write $\mathcal {L}_X Y$ for the Lie derivative of *Y* with respect to *X*, i.e.

2.1 $$\begin{aligned} \mathcal {L}_X Y=X\cdot \nabla Y -Y\cdot \nabla X. \end{aligned}$$

We denote by $\{e_1,e_2,e_3\}$ the canonical basis of $\mathbb {R}^3$.

We denote by [*M*, *N*] the quadratic covariation process, which is defined for any couple M,N of square integrable real semimartingales and we extend it by bilinearity to the analogue complex valued processes.

Let *a*, *b* be two positive numbers, then we write a≲b if there exists a positive constant *C* such that a≤Cb and $a\lesssim _\xi b$ when we want to highlight the dependence of the constant *C* on a parameter ξ. If $x\in \mathbb {C}$ we denote by $x^*$ its complex conjugate. If $\mathcal {X}$ is a tensor we denote by $\left\| \mathcal {X}\right\| _{HS}$ its Hilbert-Schmidt norm. Lastly, if $x\in \mathbb {R}^2$ we denote by $x^{\perp }=(-x_2,x_1)^t.$

### Preliminaries

#### Function spaces in the periodic setting

Let us start setting some classical notation before describing the main contributions of this work. We refer to monographs [12, 20, 23, 29–32] for a complete discussion on the topics shortly recalled in this section.

Let $\left( H^{s,p}(\mathcal {D}),\left\| \cdot \right\| _{H^{s,p}(\mathcal {D})}\right) ,\ s\in \mathbb {R},\ p\in (1,+\infty )$ be the Bessel spaces of periodic functions. In case of p=2, we simply write $H^{s}(\mathcal {D})$ in place of $H^{s,2}(\mathcal {D})$ and we denote by $\langle \cdot ,\cdot \rangle _{H^s(\mathcal {D})}$ the corresponding scalar products. When dealing with functions having $\mathbb {T}^2$ as a domain we neglect the domain in the definition of norm and inner product, i.e. we write $\left\| \cdot \right\| _{H^{s,p}}$ in place of $\left\| \cdot \right\| _{H^{s,p}(\mathbb {T}^2)}$. In case of s=0, we write $L^2(\mathcal {D})$ instead of $H^0(\mathcal {D})$ and we neglect the subscript in the notation for the norm and the inner product when considering functions having $\mathbb {T}^2$ as a domain. Instead, in case of functions having $\mathbb {T}^3_\varepsilon$ as a domain we write $\left\| \cdot \right\| _{\varepsilon }$ (resp. $\langle \cdot ,\cdot \rangle _{\varepsilon }$) in place of $\left\| \cdot \right\| _{L^2(\mathbb {T}^3_\varepsilon )}$ (resp. $\langle \cdot ,\cdot \rangle _{L^2(\mathbb {T}^3_\varepsilon )}$). For s>0, we denote the duality between $H^{-s}(\mathcal {D})$ and $H^s(\mathcal {D})$ by $\langle \cdot ,\cdot \rangle _{L^2(\mathcal {D})}$.

We denote by $\dot{H}^{s,p}(\mathcal {D})$ (resp. $\dot{H}^{s}(\mathcal {D}),\ \dot{L^2}(\mathcal {D})$) the closed subspace of $H^{s,p}(\mathcal {D})$ (resp. $H^{s}(\mathcal {D}),\ L^2(\mathcal {D})$) made by zero mean functions with norm induced by $H^{s,p}(\mathcal {D})$ (resp. $H^{s}(\mathcal {D}),\ L^2(\mathcal {D})$). Where it creates no ambiguity, we denote the Laplacian with the same symbol, Δ, either when considering functions with domain $\mathbb {T}^2, \mathbb {T}^3$ or $\mathbb {T}^3_{\varepsilon }$, i.e. we denote by

$$\begin{aligned} \Delta : \textsf{D}(\Delta )\subseteq \dot{L^2}(\mathcal {D})\rightarrow \dot{L}^2(\mathcal {D}), \end{aligned}$$

where $\textsf{D}(\Delta )=\dot{H}^2(\mathcal {D})$. We follow the same notation when considering the orthogonal projection of $L^2(\mathcal {D})$ on $\dot{L}^2(\mathcal {D})$, which we denote by $Q:L^2(\mathcal {D})\rightarrow \dot{L}^2(\mathcal {D})$. The restriction (resp. extension) of *Q* to $H^s(\mathcal {D})$ (resp. $H^{-s}(\mathcal {D})$) for s>0 is the orthogonal projection on $\dot{H}^s(\mathcal {D})$ (resp. $\dot{H}^{-s}(\mathcal {D})$). It is well known that Δ is the infinitesimal generator of analytic semigroup of negative type that we denote by $e^{t\Delta }:\dot{L}^2(\mathcal {D})\rightarrow \dot{L}^2(\mathcal {D})$ and moreover for each $\alpha \in \mathbb {R}$, $\textsf{D}((-\Delta )^{\alpha })$ can be identified with $\dot{H}^{2\alpha }(\mathcal {D})$. It is well known that the family $\{\frac{1}{2\pi \sqrt{2\pi \varepsilon }}e^{ik\cdot x}\}_{k\in \mathbb {Z}^{3,\varepsilon ^{-1}}_0}$ (resp. $\{\frac{e^{ik\cdot x}}{2\pi }\}_{k\in \mathbb {Z}^2_0}$) is a complete orthogonal system of $\dot{H}^s(\mathbb {T}^3_{\varepsilon })$ (resp. $\dot{H}^s(\mathbb {T}^2)$) made by eigenfunctions of -Δ. Moreover it is orthonormal in $\dot{L}^2(\mathbb {T}^3_{\varepsilon })$ (resp. $\dot{L}^2(\mathbb {T}^2)$). We recall for the convenience of the reader some properties of the Heat Semigroup we will exploit in the following:

##### Lemma 2.1

Let $$q\in \dot{H}^{\alpha }(\mathcal {D}),\ \alpha \in \mathbb {R}$$. Then:

i) for any $\varphi \ge 0,$ it holds $\Vert e^{t {\Delta }}q\Vert _{\dot{H}^{\alpha +\varphi }}\le C_{\varphi }t^{-\varphi /2}\Vert q\Vert _{\dot{H}^{\alpha }}$ for some constant increasing in φ;
ii) for any $\varphi \in [0,2],$ it holds $\Vert \left( I-e^{t{\Delta }}\right) q\Vert _{\dot{H}^{\alpha -\varphi }}\lesssim _{\varphi } t^{\varphi /2}\Vert q\Vert _{\dot{H}^{\alpha }}$.

Similarly, for $d\in \{2,3\}$ we introduce the Bessel spaces of zero mean vector fields

$$\begin{aligned} {H}^{s,p}(\mathcal {D};\mathbb {R}^d)&= \{u=(u_1,\dots , u_d)^t:\ u_1,\dots , u_d\in H^{s,p}(\mathcal {D})\},\\ \langle u, v\rangle _{{H}^s(\mathcal {D};\mathbb {R}^{d})}&=\sum _{j=1}^d\langle u_j,v_j\rangle _{H^s}, \quad \text {for } s\in \mathbb {R}. \end{aligned}$$

Again, in case of s=0 we write ${L}^2(\mathcal {D};\mathbb {R}^d)$ instead of ${H}^0(\mathcal {D};\mathbb {R}^d)$ and we neglect the subscript in the notation for the norm and the scalar product. Similarly we denote by $\dot{H}^s(\mathcal {D};\mathbb {R}^d)$ (resp. $\dot{L}^2(\mathcal {D};\mathbb {R}^d)$) the closed subspace of $H^s(\mathcal {D};\mathbb {R}^d)$ (resp. $L^2(\mathcal {D};\mathbb {R}^d)$) made by zero mean functions. In case of $\mathcal {D}=\mathbb {T}^{3}_{\varepsilon },\ d=3$ we denote by $\textbf{H}_{\varepsilon }^{s}$ (resp. $\textbf{L}^2_{\varepsilon }$) the closed subspace of $H^s(\mathbb {T}^3_{\varepsilon };\mathbb {R}^3)$ (resp. $L^2(\mathbb {T}^3_{\varepsilon };\mathbb {R}^3)$) made by zero mean, divergence free vector fields with norm induced by $H^s(\mathbb {T}^3_{\varepsilon };\mathbb {R}^3)$ (resp. $L^2(\mathbb {T}^3_{\varepsilon };\mathbb {R}^3)$). With some abuse of notation we usually employ the same notation for norms and inner products that we introduced for scalar functions also for the corresponding vector fields, forgetting their range and we denote by

$$\begin{aligned} (-\Delta )^{\alpha } f=((-\Delta )^{\alpha } f_1,\dots , (-\Delta )^{\alpha } f_i)^t\quad \text {for }f\in \dot{H}^{2\alpha }(\mathcal {D};{\mathbb {R}}^d). \end{aligned}$$

Having this notation in mind we recall the definition and the regularizing property of the Leray Projection and the Biot-Savart operator. We denote by

$$\begin{aligned} P_{\varepsilon }:\dot{L}^2(\mathbb {T}^3_{\varepsilon };\mathbb {R}^3)\rightarrow \textbf{L}^2_{\varepsilon } \end{aligned}$$

the Leray projection which, in the periodic setting, is an orthogonal projection even when acting between $\dot{H}^s(\mathbb {T}^{3}_{\varepsilon };\mathbb {R}^3)$ and $\textbf{H}^s_{\varepsilon }.$

If $v\in \textbf{H}_{\varepsilon }^{s}$ there exists a unique vector field $K_{\varepsilon }[v]$ such that $\operatorname {curl}K_{\varepsilon }[v]=v,\ \operatorname {div}K_{\varepsilon }[v]=0$. Moreover the operator

$$\begin{aligned} K_{\varepsilon }:\textbf{H}_{\varepsilon }^{s}\rightarrow \textbf{H}_{\varepsilon }^{s+1} \end{aligned}$$

is linear and continuous for each $s\in \mathbb {R}$ and is given by $K_{\varepsilon }[v]=(-\Delta )^{-1}\operatorname {curl}v$. In particular, if $v:\mathbb {T}^3_{\varepsilon }\rightarrow \mathbb {R}^3,\ v=(v_1,v_2,v_3)^t,$ is independent of the third component, we have

2.2 $$\begin{aligned} K_{\varepsilon }[v]=(-\nabla _H^{\perp }(-\Delta )^{-1}v^3,(-\Delta )^{-1}\nabla ^{\perp }_H\cdot v^H)^t, \end{aligned}$$

where we denoted by $\nabla ^{\perp }_H=(-\partial _2,\partial _1)$.

We conclude this subsection introducing some classical notation when dealing with stochastic processes taking values in separable Hilbert spaces. Let *Z* be a separable Hilbert space, with associated norm $\Vert \cdot \Vert _{Z}$. We denote by $C_{\mathcal {F}}\left( \left[ 0,T\right] ;Z\right)$ the space of weakly continuous adapted processes $\left( X_{t}\right) _{t\in \left[ 0,T\right] }$ with values in *Z* such that

$$ \mathbb {E} \bigg [ \sup _{t\in \left[ 0,T\right] }\left\| X_{t}\right\| _{Z}^{2}\bigg ] <\infty $$

and by $L_{\mathcal {F}}^{p}\left( 0,T;Z\right) ,\ p\in [1,\infty ),$ the space of progressively measurable processes $\left( X_{t}\right) _{t\in \left[ 0,T\right] }$ with values in *Z* such that

$$ \mathbb {E} \bigg [ \int _{0}^{T}\left\| X_{t}\right\| _{Z}^{p}dt \bigg ] <\infty . $$

#### Preliminaries on the thin layer

For our purposes, it is convenient to introduce the mean operator, $M_{\varepsilon }$, and the fluctuations operator, $N_{\varepsilon }$, in order to exploit the properties of working with a thin layer. Therefore, let us recall their definitions and some properties which we will use in the following. We refer to [24, 25, 28] for the proof of these results and some more discussions on the topic.

##### Definition 2.2

We denote by $M_{\varepsilon }:\dot{L}^2(\mathbb {T}^3_{\varepsilon })\rightarrow \dot{L}^2(\mathbb {T}^3_{\varepsilon })$ the mean operator in the third variable, i.e.

$$\begin{aligned} M_{\varepsilon }\phi (x_1,x_2)=\frac{1}{2\pi \varepsilon }\int _0^{2\pi \varepsilon } \phi (x_1,x_2,x_3)dx_3. \end{aligned}$$

Similarly $$N_{\varepsilon }=I-M_{\varepsilon }$$, therefore $$\int _0^{2\pi \varepsilon } N_{\varepsilon }\phi (x_1,x_2,x_3)dx_3=0.$$

The operator $M_{\varepsilon }$ and $N_{\varepsilon }$ are projections either in $\dot{L}^2(\mathbb {T}^3_\varepsilon )$ and in $\dot{H}^1(\mathbb {T}^3_\varepsilon )$. More generally the following holds.

##### Lemma 2.3

i) The operators $M_{\varepsilon },\ N_{\varepsilon }$ are orthogonal projections in $\dot{H}^s(\mathbb {T}^3_{\varepsilon })$ for each $s\in \mathbb {R}.$ In particular:
  2.3 $$\begin{aligned} M_{\varepsilon }N_{\varepsilon }f=N_{\varepsilon }M_{\varepsilon }f=0\quad \forall f\in \dot{H}^s(\mathbb {T}^3_{\varepsilon }). \end{aligned}$$
ii) $M_{\varepsilon },\ N_{\varepsilon }$ commute with derivative and fractional powers of the Laplacian. In particular, $M_{\varepsilon },\ N_{\varepsilon }$ commute with $K_{\varepsilon }$.
iii) For each $u,\ v,\ w:\mathbb {T}^3_{\varepsilon }\rightarrow \mathbb {R}^3$ such that $\operatorname {div}u=\operatorname {div}v=\operatorname {div}w=0$ and $\langle u\cdot \nabla v,w\rangle _{\varepsilon }$ is well defined, then we have
  2.4 $$\begin{aligned} \langle u\cdot \nabla v,M_{\varepsilon }w\rangle _{\varepsilon }=\langle M_{\varepsilon }u\cdot \nabla M_{\varepsilon }v,M_{\varepsilon }w\rangle _{\varepsilon }+\langle N_{\varepsilon }u\cdot \nabla N_{\varepsilon }v,M_{\varepsilon }w\rangle _{\varepsilon }. \end{aligned}$$
  2.5 $$\begin{aligned} \langle u\cdot \nabla v,N_{\varepsilon }w\rangle _{\varepsilon }&=\langle M_{\varepsilon }u\cdot \nabla N_{\varepsilon }v,N_{\varepsilon }w\rangle _{\varepsilon }+\langle N_{\varepsilon }u\cdot \nabla M_{\varepsilon }v,N_{\varepsilon }w\rangle _{\varepsilon } \nonumber \\&\quad +\langle N_{\varepsilon }u\cdot \nabla N_{\varepsilon }v,N_{\varepsilon }w\rangle _{\varepsilon }. \end{aligned}$$
iv) For each $f\in \dot{H}^{1}(\mathbb {T}^3_{\varepsilon })$ the following Poincaré inequality holds
  2.6 $$\begin{aligned} \left\| N_{\varepsilon }v\right\| _{{\varepsilon }}\le \varepsilon \left\| \partial _{3}N_{\varepsilon }v\right\| _{{\varepsilon }}. \end{aligned}$$

##### Remark 2.4

In our periodic setting, the mean operator $M_{\varepsilon }$ preserves Fourier modes which are independent of the third variable. Instead, the fluctuations operator $N_{\varepsilon }$ preserves Fourier modes which depend on the third variable, i.e. if $f\in \dot{H}^s({\mathbb {T}^{3}_\varepsilon })$ then

$$\begin{aligned} M_{\varepsilon }f=\sum _{\begin{array}{c} {k\in \mathbb {Z}^{3,\varepsilon ^{-1}}_0}\\ k_3=0 \end{array}}\langle f, e^{-ik\cdot x}\rangle _{\varepsilon }\frac{e^{ik\cdot x}}{8\pi \varepsilon },\quad N_{\varepsilon }f=\sum _{\begin{array}{c} k\in \mathbb {Z}^{3,\varepsilon ^{-1}}_0\\ k_3\ne 0 \end{array}}\langle f, e^{-ik\cdot x}\rangle _{\varepsilon }\frac{e^{ik\cdot x}}{8\pi \varepsilon }. \end{aligned}$$

Exploiting Fourier decomposition, one can easily prove that, more generally, if $u,\ v,\ w:\mathbb {T}^3_{\varepsilon }\rightarrow \mathbb {R}$ are regular enough such that $uv\in H^s(\mathbb {T}^3),\ s\in \mathbb {R}$ is well defined (e.g. $s=0,\ u,v\in \dot{L}^4(\mathbb {T}^3_{\varepsilon })$), then we have

$$\begin{aligned} M_{\varepsilon }(Q[uv])&=Q[M_{\varepsilon }u M_{\varepsilon }v]+M_{\varepsilon }\left( Q[N_{\varepsilon }uN_{\varepsilon }v]\right) ,\quad N_{\varepsilon }(Q[uv])\\&=M_{\varepsilon }{u}N_{\varepsilon }v+N_{\varepsilon }uM_{\varepsilon }v+N_{\varepsilon }(Q[N_{\varepsilon }uN_{\varepsilon }v]). \end{aligned}$$

### Description of the stochastic system

We now move to the description of the random velocity field appearing in equation (1.1). We shall define it in a sort of expansion in the Fourier basis. For each $k\in \mathbb {Z}^{3,\varepsilon ^{-1}}_0,\ j\in \{1,2\}$ we denote by $\sigma _{k,j}^{\varepsilon }(x)=\theta _{k,j}^{\varepsilon }a_{k,j}e^{ik\cdot x}$, where $\{\frac{k}{|k|}, a_{k,1}, a_{k,2}\}$ is an orthonormal system of $\mathbb {R}^3$ for $k_3> 0$ and $a_{k,j}=a_{-k,j}$ if $k_3<0$, while the (complex) coefficients $\theta _{k,j}^\varepsilon$ will be fixed later. It is well known that the family $\{a_{k,j}\frac{1}{2\pi \sqrt{2\pi \varepsilon }}e^{ik\cdot x}\}, \ {{k\in \mathbb {Z}^{3,\varepsilon ^{-1}}_0, \ j\in \{1,2\}}}$ is a complete orthogonal system of $\textbf{H}^s_{\varepsilon }$ made by eigenfunctions of -Δ. Moreover it is orthonormal in $\textbf{L}^2_{\varepsilon }$. Without loss of generality, we make a choice that will simplify some of the computations: when $k_3=0$ we choose $a_{k,1}=(-k_2/|k|,k_1/|k|,0),\ a_{k,2}=(0,0,1)$ if $k\in \Gamma _{+}^{\varepsilon }$, $a_{k,j}=a_{-k,j}$ otherwise. Now we define the Brownian motions. Our construction aims at obtaining a non trivial space covariance structure, while retaining the description in the Fourier basis, since this choice will simplify many computations thanks to Remark 2.4. We begin with a correlation factor $\rho \in [-1,1]$. Then assume $\left( \Omega ,\mathcal {F},\mathcal {F}_t,\mathbb {P}\right)$ is a filtered probability space such that $(\Omega , \mathcal {F},\mathbb {P})$ is a complete probability space, $(\mathcal {F}_t)_{t\in [0,T]}$ is a right continuous filtration and $\mathcal {F}_0$ contains every $\mathbb {P}$ null subset of Ω. Now, for each $N\in \mathbb {N}$, $\{W_t^{k,j}\}, \ {{k\in \mathbb {Z}^{3,N}_0, \ j\in \{1,2\}}}$ is a sequence of complex-valued Brownian motions adapted to $\mathcal {F}_t$ such that $W^{-k,j}_t=(W^{k,j}_t)^*$ and

$$\begin{aligned}&\mathbb {E} \left[ W^{k,j}_1,{W^{l,m}_1}^*\right] ={\left\{ \begin{array}{ll} 2 &  \textit{if } k=l, \ m=j\\ 2\rho &  \textit{if } k=l,\ k_3=0,\ m\ne j; \end{array}\right. }\\&\left[ W^{k,j}_\cdot , W^{l,m}_\cdot \right] _t={\left\{ \begin{array}{ll} 2t &  \textit{if } k=-l, \ m=j\\ 2\rho t &  \textit{if } k=-l,\ k_3=0,\ m\ne j. \end{array}\right. } \end{aligned}$$

With the notation above in mind, we introduce the random field

$$\begin{aligned} W^{\varepsilon }_t:=\sum _{\begin{array}{c} k\in \mathbb {Z}^{3,\varepsilon ^{-1}}_0\\ j\in \{1,2\} \end{array}}\sigma _{k,j}^{\varepsilon }W^{k,j}_t, \end{aligned}$$

with $\sigma _{k,j}^{\varepsilon }=\theta _{k,j}^{\varepsilon }a_{k,j}e^{ik\cdot x}$. We will formally consider $v^{\varepsilon }=\partial _t W^{\varepsilon }$ in (1.1) and postpone to Section 2.4 for the rigorous definition of (1.1) for this choice of $v^{\varepsilon }.$ The physical intuition behind our construction is discussed in Remark 2.11 below. The role of the factor ρ in the definition above is to introduce some correlation between the vertical and horizontal components of the ‘2D’ modes (those who have $k_3=0$) of the random field. With our choices, to ensure that $W^\varepsilon$ takes values in a space of real valued functions, the coefficients must satisfy the condition

2.7 $$\begin{aligned} {\theta _{k,j}^{\varepsilon }}^{*}=\theta _{-k,j}^\varepsilon .\end{aligned}$$

#### Remark 2.5

The complex Brownian motions $W^{k,j}_t$ required can be constructed in the following way: let $$\{B_t^{k,j}\}_{\begin{array}{c} k\in \mathbb {Z}^{3,N}_0\\ j\in \{1,2\} \end{array}}$$ be a sequence of real, Brownian motions adapted to $\mathcal {F}_t$ such that

$$\begin{aligned} \mathbb {E} \left[ B^{k,j}_t B^{l,m}_s\right] =\left( t\wedge s\right) \left( \delta _{j,m}\delta _{k,l}+\rho \delta _{|j-m|=1}\delta _{k,l}\delta _{k_3,0}\right) \end{aligned}$$

and then set

$$\begin{aligned} W^{k,j}_t={\left\{ \begin{array}{ll} B^{k,j}_t+iB^{-k,j}_t&  \text {if } k\in \Gamma ^{\varepsilon }_+ \\ B^{-k,j}_t-iB^{k,j}_t &  \text {if } k\in \Gamma ^{\varepsilon }_-. \end{array}\right. } \end{aligned}$$

Finally, we make an explicit choice of the coefficients $\theta _{k,j}^{\varepsilon }$ appearing in the definition of our random velocity field $W^{\varepsilon }_t$:

2.8 $$\begin{aligned} \theta _{k,j}^{\varepsilon }&=\boldsymbol{1}_{\{N\le |k_H|\le 2N\} }{\left\{ \begin{array}{ll} \frac{i}{C_{1,H}|k|}&  \text {if } k_3=0, k\in \Gamma _{+}^{\varepsilon },\ j=1\\ \frac{-i}{C_{1,H}|k|}&  \text {if } k_3=0, k\in \Gamma _{-}^{\varepsilon },\ j=1\\ \frac{1}{C_{2,H}|k|^{\gamma /2}}&  \text {if } k_3=0,\ j=2\\ \frac{1}{C_{V}|k|^{\beta /2}}&  \text {if } k_3\ne 0. \end{array}\right. } \end{aligned}$$

The parameters $\varepsilon , N, C_{1,H}, C_{2,H}, C_{V},\beta ,\gamma$ cannot be arbitrary. Indeed we will always assume in the following even if not specified:

#### Hypothesis 2.6

- The width of the thin layer is $2\pi \varepsilon =\frac{2\pi }{N}$, i.e. $\varepsilon =\frac{1}{N}$ for $N\in \mathbb {N}$.
- The coefficients in (2.8) satisfy $\beta \ge 4,\ \gamma \ge 4$ and $C_{1,H}>\sqrt{\frac{\zeta _{H,0}}{\eta }},\ C_{2,H},\ C_V>0$.

More general choices for the phase and the sign of our coefficients are allowed, although the computations and the notation get heavier, while the decay rate ($\beta , \gamma \ge 4$) is optimal in our setting. For this reason we decide to avoid this level of generality.

#### Remark 2.7

Due to our choice of the coefficients $\theta _{k,j}^{\varepsilon }$ and the vectors $a_{k,j}$ it follows that for each *k* such that $k_3=0,\ N\le |k|\le 2N$

$$\begin{aligned} \theta _{k,1}^{\varepsilon } a_{k,1}=\frac{i}{C_{1,H}}\frac{k^{\perp }}{|k|^2},\ \theta _{k,2}^{\varepsilon } a_{k,2}=\frac{1}{C_{2,H}}\frac{e_3}{|k|^{\gamma /2}}. \end{aligned}$$

Therefore the 2D modes of our noise naturally split in horizontal and vertical components

$$\begin{aligned} \overline{W^{\varepsilon ,H}}:= \sum _{\begin{array}{c} k\in \mathbb {Z}_0^{3, N} \\ {k_3=0, j=1} \end{array}}\sigma _{k,j}^{\varepsilon }W^{k,j} \qquad \overline{W^{\varepsilon ,3}}:= \sum _{\begin{array}{c} k\in \mathbb {Z}_0^{3, N} \\ {k_3=0, j=2} \end{array}}\sigma _{k,j}^{\varepsilon }W^{k,j}. \end{aligned}$$

Moreover the components satisfy the relations

$$\begin{aligned} \overline{W^{\varepsilon ,H}}(-x)=-\overline{W^{\varepsilon ,H}}(x), \qquad \overline{W^{\varepsilon , 3}}(-x)=\overline{W^{\varepsilon , 3}}(x). \end{aligned}$$

As shown in [9], this anisotropy of the noise is the main responsible for the appearance of the alpha-term in this kind of models.

In the next few pages, we introduce several reformulations and properties of the spatial covariance of our noise. While all of them will be used later on, eager readers may wish to skip directly to Section 2.4 and return here if any notation or statements are unclear. The space covariance function associated to our noise is

2.9 $$\begin{aligned}&Q^{\varepsilon }(x,y)=\mathbb {E} \left[ W^{\varepsilon }_1(x) \otimes {W^{\varepsilon }_1(y)}^* \right] =Q^{\varepsilon }_0(x,y)+\overline{Q^{\varepsilon }_{\rho }}(x,y)\nonumber \\&\quad =\overline{Q^{\varepsilon }}(x,y)+\left( Q^{\varepsilon }\right) '(x,y)+\overline{Q^{\varepsilon }_{\rho }}(x,y) \end{aligned}$$

where we denoted by

$$\begin{aligned} \overline{Q^{\varepsilon }}(x,y)&=2\sum _{\begin{array}{c} k\in \mathbb {Z}^{3,N}_0\\ k_3=0,\quad j\in \{1,2\} \end{array}}\left\lvert \theta _{k,j}^{\varepsilon }\right\rvert ^2 a_{k,j}\otimes a_{k,j}e^{ik\cdot (x-y)},\quad \left( Q^{\varepsilon }\right) '(x,y)\\&\quad =2\sum _{\begin{array}{c} k\in \mathbb {Z}^{3,N}_0\\ k_3\ne 0,\quad j\in \{1,2\} \end{array}}\left\lvert \theta _{k,j}^{\varepsilon }\right\rvert ^2 a_{k,j}\otimes a_{k,j}e^{ik\cdot (x-y)},\\ \overline{Q^{\varepsilon }_{\rho }}(x,y)&=2\rho \sum _{\begin{array}{c} k\in \mathbb {Z}^{3,N}_0\\ k_3=0 \end{array}}\left( \theta _{k,1}^{\varepsilon }\left( \theta _{k,2}^{\varepsilon }\right) ^* a_{k,1}\otimes a_{k,2}+\theta _{k,2}^{\varepsilon }\left( \theta _{k,1}^{\varepsilon }\right) ^* a_{k,2}\otimes a_{k,1}\right) e^{ik\cdot (x-y)}. \end{aligned}$$

Due to our choice, we have that $\overline{Q^{\varepsilon }}(x,y),$ $\left( Q^{\varepsilon }\right) '(x,y)$ and $\overline{Q^{\varepsilon }_{\rho }}(x,y)$ are all translation invariant, therefore we simply write $Q^{\varepsilon }(x-y)$ (resp. $Q^{\varepsilon }_0(x-y),\ \overline{Q^{\varepsilon }}(x-y),\ \left( Q^{\varepsilon }\right) '(x-y),\ \overline{Q^{\varepsilon }_{\rho }}(x-y)$) in place of $Q^{\varepsilon }(x,y)$ (resp. $Q^{\varepsilon }_0(x,y),\ \overline{Q^{\varepsilon }}(x,y),\ \left( Q^{\varepsilon }\right) '(x,y),\ \overline{Q^{\varepsilon }_{\rho }}(x,y)$). In particular $Q^{\varepsilon }_0(x),\ \overline{Q^{\varepsilon }}(x),\ \left( Q^{\varepsilon }\right) '(x)$ are mirror symmetric, namely it holds

2.10 $$\begin{aligned} Q^{\varepsilon }_0(x)=Q^{\varepsilon }_0(-x),\ \overline{Q^{\varepsilon }}(x)=\overline{Q^{\varepsilon }}(-x),\ \left( Q^{\varepsilon }\right) '(x)=\left( Q^{\varepsilon }\right) '(-x). \end{aligned}$$

On the contrary $\overline{Q^{\varepsilon }_{\rho }}(x)$ is an odd function, in particular $\overline{Q^{\varepsilon }_{\rho }}(0)=0$. As a consequence, our noise satisfies the mirror symmetry property if and only if ρ=0. Let us understand better the behaviour of $Q^{\varepsilon }(0)$. Indeed, as discussed in [12, Chapter 3], it plays a crucial role in order to provide the dissipation properties of our limit object. Let us start considering $\left( Q^{\varepsilon }\right) '(0)$, we have:

$$\begin{aligned} \left( Q^{\varepsilon }\right) '(0)=2\sum _{\begin{array}{c} k\in \mathbb {Z}^{3,N}_0\\ N\le |k_H|\le 2N \end{array}}\frac{1}{C_V^2|k|^{\beta }}\left( I-\frac{k\otimes k}{|k|^2}\right) . \end{aligned}$$

Arguing as in [14, Section 2.3], it follows easily that $\left( Q^{\varepsilon }\right) '(0)$ is a diagonal matrix, moreover it holds

$$\begin{aligned} \left( Q^{\varepsilon }\right) '(0)=2(e_1\otimes e_1+e_2\otimes e_2)\eta ^{\varepsilon }_{V,T}+2(e_3\otimes e_3)\eta _{V,R}^\varepsilon , \end{aligned}$$

where we denoted by

$$\begin{aligned} \eta ^{\varepsilon }_{V,T}&=\sum _{\begin{array}{c} k\in \mathbb {Z}^{3,N}_0,\ k_3\ne 0\\ N\le |k_H|\le 2N \end{array}}\frac{1}{C_V^2|k|^{\beta }}\left( 1-\frac{|k_H|^2}{2|k|^2}\right) ,\quad \eta _{V,R}^\varepsilon \\&=\sum _{\begin{array}{c} k\in \mathbb {Z}^{3,N}_0,\ k_3\ne 0\\ N\le |k_H|\le 2N \end{array}}\frac{1}{C_V^2|k|^{\beta }}\left( 1-\frac{|k_3|^2}{|k|^2}\right) . \end{aligned}$$

In particular both $\eta ^{\varepsilon }_{V,T}, \ \eta ^{\varepsilon }_{V,R}$ go to 0 as $\varepsilon \rightarrow 0$. Indeed it holds

2.11 $$\begin{aligned} \eta ^{\varepsilon }_{V,T}+\eta ^{\varepsilon }_{V,R}&\lesssim \sum _{\begin{array}{c} k\in \mathbb {Z}^{3,N}_0, \ k_3\ne 0\\ N\le |k_H|\le 2N \end{array}}\frac{1}{\left( |k_H|^{2}+|k_3|^2\right) ^{\beta /2}}\nonumber \\  &\le \sum _{\begin{array}{c} k_H\in \mathbb {Z}^2_0\\ N\le |k_H|\le 2N \end{array}}\sum _{k_3\in \mathbb {Z},\ k_3\ne 0} \frac{1}{\left( 1+|k_3|^2\right) ^{\beta /2}N^{\beta }}\nonumber \\  &\le \zeta ^N_{H,0}\zeta _{\beta }\frac{1}{N^{\beta -2}}=O(N^{2-\beta }). \end{aligned}$$

In order to treat $\overline{Q^{\varepsilon }}(0)$ we introduce a further splitting of the sum. We write

$$\begin{aligned} \overline{Q^{\varepsilon }}(x)=\overline{Q_T^{\varepsilon }}(x)+\overline{Q_R^{\varepsilon }}(x), \end{aligned}$$

denoting by

$$\begin{aligned} \overline{Q^{\varepsilon }_T}(x)&=2\sum _{\begin{array}{c} k\in \mathbb {Z}^{3,N}_0\\ k_3=0,\quad j=1 \end{array}}\left\lvert \theta _{k,j}^{\varepsilon }\right\rvert ^2 a_{k,j}\otimes a_{k,j}e^{ik\cdot x},\quad \overline{Q^{\varepsilon }_R}(x)\\&=2\sum _{\begin{array}{c} k\in \mathbb {Z}^{3,N}_0\\ k_3=0,\quad j=2 \end{array}}\left\lvert \theta _{k,j}^{\varepsilon }\right\rvert ^2 a_{k,j}\otimes a_{k,j}e^{ik\cdot x}. \end{aligned}$$

Of course, $\overline{Q^{\varepsilon }_T}(x), \overline{Q^{\varepsilon }_R}(x)$ satisfy mirror symmetry property too. By definition it holds

$$\begin{aligned} \overline{Q^{\varepsilon }_R}(0)=2\left( e_3\otimes e_3\right) \eta ^{\varepsilon }_{H,R},\quad \eta ^{\varepsilon }_{H,R}=\sum _{\begin{array}{c} k_H\in \mathbb {Z}^2_0\\ N\le |k_H|\le 2N \end{array}}\frac{1}{C_{2,H}^2|k_H|^{\gamma }}. \end{aligned}$$

Therefore also $\eta ^{\varepsilon }_{H,R}$ goes to 0 as $\varepsilon \rightarrow 0$. Indeed, we have

2.12 $$\begin{aligned} \eta ^{\varepsilon }_{H,R}=\frac{\zeta ^N_{H,\gamma }N^{2-\gamma }}{C_{2,H}^2}=O(N^{2-\gamma }). \end{aligned}$$

Lastly we need to study the asymptotic behaviour of $\overline{Q^{\varepsilon }_T}(0)$ which is the one we will keep track in the scaling limit. Arguing as in [14, Section 2.3], it follows easily that also $\overline{Q^{\varepsilon }_T}(0)$ is a diagonal matrix, moreover it holds

$$\begin{aligned} \overline{Q^{\varepsilon }_T}(0)=\frac{\zeta _{H,2}^N}{C_{1,H}^2}(e_1\otimes e_1+e_2\otimes e_2)=\frac{\zeta _{H,2}}{C_{1,H}^2}(e_1\otimes e_1+e_2\otimes e_2)+O(N^{-1}). \end{aligned}$$

We set

$$\begin{aligned}&\eta ^{\varepsilon }_T=\eta ^{\varepsilon }_{V,T}+\frac{\zeta ^N_{H,2}}{C_{1,H}^2}=\frac{\zeta _{H,2}}{C_{1,H}^2}+O(N^{-1}), \quad \eta ^{\varepsilon }_R=\eta ^{\varepsilon }_{V,R}+\eta ^{\varepsilon }_{H,R}=O(N^{-4}), \\&\quad \quad \eta _T=\frac{\zeta _{H,2}}{C_{1,H}^2}. \end{aligned}$$

We state now some results on the covariance functions $Q^{\varepsilon }(x),$ $Q^{\varepsilon }_0(x),$ $\overline{Q_{\rho }^{\varepsilon }}(x),$ $\overline{Q^{\varepsilon }}(x),$ $\left( Q^{\varepsilon }\right) '(x),$ $\overline{Q_T^{\varepsilon }}(x),$ $\overline{Q_R^{\varepsilon }}(x)$. The first ones are closely related to the invariance by translation of the covariance functions, therefore they hold for all of them. We give the detailed statement and proof only for $Q^{\varepsilon }_0(x)$, the others being analogous.

#### Lemma 2.8

For each $l,m\in \{1,2,3\}$ it holds

2.13 $$\begin{aligned} \left( \partial _{l} Q^{\varepsilon }_0\right) (x-y)&=\partial _{x_l} Q^{\varepsilon }_0(x-y)=2i\sum _{\begin{array}{c} k\in \mathbb {Z}^{3,N}_0\\ j\in \{1,2\} \end{array}}\left\lvert \theta _{k,j}^{\varepsilon }\right\rvert ^2 k_l a_{k,j}\otimes a_{k,j}e^{ik\cdot (x-y)},\end{aligned}$$

2.14 $$\begin{aligned} \left( \partial _{l}\partial _m Q^{\varepsilon }_0\right) (x-y)&=-\partial _{x_l}\partial _{y_m}Q^{\varepsilon }_0(x-y)\nonumber \\&=-2\sum _{\begin{array}{c} k\in \mathbb {Z}^{3,N}_0\\ j\in \{1,2\} \end{array}}\left\lvert \theta _{k,j}^{\varepsilon }\right\rvert ^2 k_l k_m a_{k,j}\otimes a_{k,j}e^{ik\cdot (x-y)}. \end{aligned}$$

In particular

2.15 $$\begin{aligned} \left( \partial _{l} Q^{\varepsilon }_0\right) (0)&=2i\sum _{\begin{array}{c} k\in \mathbb {Z}^{3,N}_0\\ j\in \{1,2\} \end{array}}\left\lvert \theta _{k,j}^{\varepsilon }\right\rvert ^2 k_l a_{k,j}\otimes a_{k,j},\end{aligned}$$

2.16 $$\begin{aligned} \left( \partial _{l}\partial _m Q^{\varepsilon }_0\right) (0)&=-2\sum _{\begin{array}{c} k\in \mathbb {Z}^{3,N}_0\\ j\in \{1,2\} \end{array}}\left\lvert \theta _{k,j}^{\varepsilon }\right\rvert ^2 k_l k_m a_{k,j}\otimes a_{k,j}. \end{aligned}$$

#### Proof

$\left( \partial _{l} Q^{\varepsilon }_0\right) (x-y)=\partial _{x_l} Q^{\varepsilon }_0(x-y)$ and $\left( \partial _{l}\partial _m Q^{\varepsilon }_0\right) (x-y)=-\partial _{x_l}\partial _{y_m}Q^{\varepsilon }_0(x-y)$ by the chain rule. Then (2.13) and (2.14) follow from the definition of $Q^{\varepsilon }_0(x-y).$ Setting y=x in the right hand side of (2.13) (resp. (2.14)) we get immediately

$$\begin{aligned} \left( \partial _{l} Q^{\varepsilon }_0\right) (0)&=2i\sum _{\begin{array}{c} k\in \mathbb {Z}^{3,N}_0\\ j\in \{1,2\} \end{array}}\left\lvert \theta _{k,j}^{\varepsilon }\right\rvert ^2 k_l a_{k,j}\otimes a_{k,j}\\ \text {(resp. } \left( \partial _{l}\partial _m Q^{\varepsilon }_0\right) (0)&=-2\sum _{\begin{array}{c} k\in \mathbb {Z}^{3,N}_0\\ j\in \{1,2\} \end{array}}\left\lvert \theta _{k,j}^{\varepsilon }\right\rvert ^2 k_l k_m a_{k,j}\otimes a_{k,j}\text {)}. \end{aligned}$$

□

The other results we need on the covariance functions are strongly related to the mirror symmetry property, therefore they hold true only for $$Q^{\varepsilon }_0(x), \overline{Q^{\varepsilon }}(x),\ \left( Q^{\varepsilon }\right) '(x),\ \overline{Q_T^{\varepsilon }}(x), $$$$\ \overline{Q_R^{\varepsilon }}(x)$$. We give the detailed statement and proof only for $$Q^{\varepsilon }_0(x)$$, the others being analogous.

#### Lemma 2.9

It holds

2.17 $$\begin{aligned} \left( \partial _{l} Q^{\varepsilon }_0\right) (0)&=2i\sum _{\begin{array}{c} k\in \mathbb {Z}^{3,N}_0\\ j\in \{1,2\} \end{array}}\left\lvert \theta _{k,j}^{\varepsilon }\right\rvert ^2 k_l a_{k,j}\otimes a_{k,j}=0. \end{aligned}$$

As a consequence, for each $l,m,n\in \{1,2,3\}$ and $f\in L^2(\mathbb {T}^3_{\varepsilon }),\ g\in H^1(\mathbb {T}^3_{\varepsilon })$ it holds

2.18 $$\begin{aligned} \sum _{\begin{array}{c} k\in \mathbb {Z}^{3,N}_0\\ j\in \{1,2\} \end{array}}\langle \partial _l g\sigma _{k,j}^{l,\varepsilon }, f\partial _m \sigma _{-k,j}^{n,\varepsilon }\rangle _{\varepsilon }=0. \end{aligned}$$

#### Proof

Since by the mirror symmetry property of the covariance function (2.10), the function $Q^{\varepsilon }_0(x)$ is even and smooth, then $\left( \partial _{l} Q^{\varepsilon }_0\right) (0)=0$ and (2.17) follows. In order to complete the proof, let us expand the left hand side of (2.18), obtaining by definition of the $\sigma _{k,j}^{\varepsilon }$ above

$$\begin{aligned} \sum _{\begin{array}{c} k\in \mathbb {Z}^{3,N}_0\\ j\in \{1,2\} \end{array}}\langle \partial _l g\sigma _{k,j}^{l,\varepsilon }, f\partial _m \sigma _{-k,j}^{n,\varepsilon }\rangle _{\varepsilon }=\langle f ,\partial _l g\rangle _{\varepsilon } \sum _{\begin{array}{c} k\in \mathbb {Z}^{3,N}_0\\ j\in \{1,2\} \end{array}}\left\lvert \theta _{k,j}^{\varepsilon }\right\rvert ^2 k_m (a_{k,j})_l (a_{k,j})_n=0 \end{aligned}$$

due to (2.17). □

On the contrary, as discussed in [9], the asymptotic behaviour of $\nabla \overline{Q^{\varepsilon }_{\rho }}(0)$ plays a crucial role in order to understand the alpha-term of our model. Therefore, the last result we provide in this section is a convergence property of the matrix

2.19 $$\begin{aligned} \{{\mathcal {R}_{\varepsilon ,\gamma }}^{m,n}\}_{m,n\in \{1,2\}}=\partial _{n}\overline{Q^{\varepsilon ,3,m}_{\rho }}(0) \end{aligned}$$

and a uniform bound on $\nabla \overline{Q^{\varepsilon }_{\rho }}(0)$.

#### Lemma 2.10

It holds

$$\begin{aligned} \left\| \nabla \overline{Q^{\varepsilon }_{\rho }}(0)\right\| _{HS}\le \frac{2\sqrt{2}|\rho |\zeta ^N_{H,\gamma /2} N^{2-\gamma /2} }{C_{1,H}C_{2,H}}. \end{aligned}$$

In particular

$$\begin{aligned} \{{\mathcal {R}_{\varepsilon ,\gamma }}^{m,n}\}_{m,n\in \{1,2\}}=\partial _{n}\overline{Q^{\varepsilon ,3,m}_{\rho }}(0)= \frac{\pi \rho \zeta _{H,\gamma /2}}{C_{1,H}C_{2,H}}\begin{bmatrix} 0 &  -1\\ 1 &  0 \end{bmatrix} N^{2-\gamma /2}+O(N^{1-\gamma /2}). \end{aligned}$$

#### Proof

Thanks to (2.15) and Remark 2.7 it holds

2.20 $$\begin{aligned} \nabla \overline{Q^{\varepsilon }_{\rho }}(0)=\frac{2\rho }{C_{1,H}C_{2,H}}\sum _{\begin{array}{c} k_H\in \mathbb {Z}^2_0\\ N\le |k_H|\le 2N \end{array}}\frac{1}{|k_H|^{\gamma /2}} \left( e_3 \otimes \frac{k_H^{\perp }}{|k_H|}\otimes \frac{k_H}{|k_H|}-\frac{k_H^{\perp }}{|k_H|}\otimes e_3\otimes \frac{k_H}{|k_H|}\right) . \end{aligned}$$

Therefore we have

2.21 $$\begin{aligned} \left\| \nabla \overline{Q^{\varepsilon }_{\rho }}(0)\right\| _{HS}&\le \frac{2|\rho |}{C_{1,H} C_{2,H}}\sum _{\begin{array}{c} k_H\in \mathbb {Z}^2_0\\ N\le |k_H|\le 2N \end{array}}\frac{1}{|k_H|^{\gamma /2}}\left\| \left( e_3 \otimes \frac{k_H^{\perp }}{|k_H|}\otimes \frac{k_H}{|k_H|}-\frac{k_H^{\perp }}{|k_H|}\otimes e_3\otimes \frac{k_H}{|k_H|}\right) \right\| _{HS}\nonumber \\  &= \frac{2\sqrt{2}|\rho |\zeta ^N_{H,\gamma /2} N^{2-\gamma /2} }{C_{1,H}C_{2,H}}. \end{aligned}$$

Due to (2.20), it holds

2.22 $$\begin{aligned} \partial _{\cdot }\overline{Q^{\varepsilon ,3,\cdot }_{\rho }}(0)&=\frac{2\rho }{C_{1,H}C_{2,H}}\sum _{\begin{array}{c} k_H\in \mathbb {Z}^2_0\\ N\le |k_H|\le 2N \end{array}}\frac{1}{|k_H|^{\gamma /2}} \frac{k_H^{\perp }}{|k_H|} \otimes \frac{k_H}{|k_H|}\nonumber \\  &=\frac{2\rho }{C_{1,H}C_{2,H}N^{\gamma /2-2}}\left( \frac{1}{N^2}\sum _{\begin{array}{c} k_H\in \mathbb {Z}^2_0\\ 1\le \frac{|k_H|}{N}\le 2 \end{array}} \frac{N^{\gamma /2}}{|k_H|^{\gamma /2}} \frac{k_H^{\perp }}{|k_H|} \otimes \frac{k_H}{|k_H|}\right) . \end{aligned}$$

The function $f_{\gamma }(x)=\frac{1}{|x|^{\gamma /2+2}} (x^{\perp }\otimes x)$ is smooth for $1\le |x |\le 2$, hence the Riemann sums satisfy

2.23 $$\begin{aligned} \frac{1}{N^2}\sum _{\begin{array}{c} k_H\in \mathbb {Z}^2_0\\ 1\le \frac{|k_H|}{N}\le 2 \end{array}} \frac{N^{\gamma /2}}{|k_H|^{\gamma /2}} \frac{k_H^{\perp }}{|k_H|} \otimes \frac{k_H}{|k_H|}=\int _{1\le x\le 2} f_\gamma (x) dx+O(N^{-1}). \end{aligned}$$

Since we have

2.24 $$\begin{aligned} \int _{1\le x\le 2} f_\gamma (x) dx&=\int _1^2 \int _0^{2\pi } \frac{1}{r^{\gamma /2-1}}\begin{bmatrix} -\sin (\theta )\cos (\theta ) &  -\sin ^2(\theta ) \\ \cos ^2(\theta ) &  \sin (\theta )\cos (\theta ) \end{bmatrix} dr d\theta \nonumber \\&=\frac{\zeta _{H,\gamma /2}}{2}\begin{bmatrix} 0 &  -1\\ 1 &  0 \end{bmatrix} , \end{aligned}$$

combining (2.22), (2.23) and (2.24) the claim follows. □

#### Remark 2.11

We highlight that our noise is an adaptation of the one constructed in [9] for a 2D-3C model, where a certain helicity of the noise is imposed to achieve an alpha-term in the limit. In that work the authors constructed a random field based on the notion of a ‘cylinder of vorticity’. A cylinder of vorticity is for them a special 3D vector field *V*(*x*) on $\mathbb {T}^2$ that is decomposed as $V_H$, an horizontal component corresponding ideally to a point vortex, and $V_3 e_3$ a vertical component, with the property that $V_H$ is an odd function of *x*, while $V_3$ is even. By randomizing the position of this cylinder, they were able to use the covariance matrix of the field to define a Brownian motion. While we could not give the same description here, since we needed to express the noise in the Fourier basis to exploit crucial simplifications (*cf.* Remark 2.4), our noise retains all the properties of the one introduced in [9]. In particular, by splitting our noise as $W^\varepsilon (t,x)= \overline{W^\varepsilon }(t,x) + \left( W^\varepsilon (t,x)\right) '$, where the first part comprises all the wavenumbers with $k_3=0$, and recalling the further orthogonal decomposition $\overline{W^\varepsilon }(t,x) = \overline{W^{\varepsilon ,H}}(t,x) + \overline{W^{\varepsilon ,3}}(t,x)e_3$, we see that this part of the noise corresponds exactly to theirs. Indeed, in order to have a nonzero ‘off diagonal’ term $\overline{Q^\varepsilon _{\rho }}(0)$ in the covariance, we had to introduce a correlation between the horizontal and the vertical component, which was inherent in the structure of the cylinder, and breaks mirror symmetry for the horizontal component. In particular, due to the special choice of the coefficients we made, $\overline{W^{\varepsilon ,H}}$ is an odd function of *x*, while $\overline{W^{\varepsilon ,3}}$ is even, exactly like in the cylinder case of [9]. Finally, it is easy to check that our main assumption on the decay of the noise coefficients ($\beta , \gamma \ge 4$, *cf.* Hypothesis 2.6) is equivalent to the main assumption of [9], that is, a uniform control on the second derivatives of the covariance matrix, see [9, Hypothesis 3.3]. In Appendix B we compute the mean helicity of our noise and stress its relation with the strength of the alpha-term in the limit PDE.

### Main results

Fix $T\in (0,+\infty )$. Following the ideas introduced in Section 1, due to our choice of $v^{\varepsilon }$ as detailed in subsection 2.3, we are interested in studying the properties of the following stochastic model on $\mathbb {T}^3_{\varepsilon }$:

2.25 $$\begin{aligned} {\left\{ \begin{array}{ll} dB^{\varepsilon }_{t}& =\eta \Delta B^{\varepsilon }_{t} dt - \sum _{\begin{array}{c} k\in \mathbb {Z}^{3,N}_0\\ j\in \{1,2\} \end{array}}\mathcal {L}_{\sigma _{k,j}^{\varepsilon }}B^{\varepsilon }_t \circ dW^{k,j}_t, \\ \operatorname {div}B^{\varepsilon }_t& =0, \\ B^{\varepsilon }_t|_{t=0}& =B^{\varepsilon }_0. \end{array}\right. } \end{aligned}$$

We recall that the term $\mathcal {L}_{\sigma _{k,j}^{\varepsilon }}B^{\varepsilon }_t$ above is the Lie derivative of $B^{\varepsilon }_t$ with respect to $\sigma _{k,j}^{\varepsilon }$ introduced in (2.1). In order to study (2.25), we start rewriting it in Itô form. First, let us observe that for each $k\in \mathbb {Z}^{3,N}_0,\ j\in \{1,2\}$ we have due to the linearity of $\mathcal {L}_{\sigma _{k,j}^{\varepsilon }}$

$$\begin{aligned} \mathcal {L}_{\sigma _{k,j}^{\varepsilon }}B^{\varepsilon }_t \circ dW^{k,j}_t&=\mathcal {L}_{\sigma _{k,j}^{\varepsilon }}B^{\varepsilon }_t dW^{k,j}_t+\frac{1}{2}\left[ \mathcal {L}_{\sigma _{k,j}^{\varepsilon }}B^{\varepsilon }_\cdot , W^{k,j}_\cdot \right] _t \\  &= \mathcal {L}_{\sigma _{k,j}^{\varepsilon }}B^{\varepsilon }_t dW^{k,j}_t+\frac{1}{2}\mathcal {L}_{\sigma _{k,j}^{\varepsilon }}\left[ B^{\varepsilon }_\cdot , W^{k,j}_\cdot \right] _t\\  &=\mathcal {L}_{\sigma _{k,j}^{\varepsilon }}B^{\varepsilon }_t dW^{k,j}_t-\mathcal {L}_{\sigma _{k,j}^{\varepsilon }}\mathcal {L}_{\sigma _{-k,j}^{\varepsilon }}B^{\varepsilon }_t dt-\rho \boldsymbol{1}_{\{k_3=0,\ l\ne j\}} \mathcal {L}_{\sigma _{k,j}^{\varepsilon }}\mathcal {L}_{\sigma _{-k,l}^{\varepsilon }}B^{\varepsilon }_t dt. \end{aligned}$$

Therefore the equation for $B^{\varepsilon }_t$ can be rewritten as

$$\begin{aligned} {\left\{ \begin{array}{ll} dB^{\varepsilon }_{t}& =\left( \eta \Delta +\sum _{\begin{array}{c} k\in \mathbb {Z}^{3,N}_0\\ j\in \{1,2\} \end{array}}\mathcal {L}_{\sigma _{k,j}^{\varepsilon }}\mathcal {L}_{\sigma _{-k,j}^{\varepsilon }}+\rho \sum _{\begin{array}{c} k\in \mathbb {Z}^{3,N}_0\\ k_3=0 \end{array}}\mathcal {L}_{\sigma _{k,1}^{\varepsilon }}\mathcal {L}_{\sigma _{-k,2}^{\varepsilon }}+\mathcal {L}_{\sigma _{k,2}^{\varepsilon }}\mathcal {L}_{\sigma _{-k,1}^{\varepsilon }} \right) B^{\varepsilon }_{t} dt\\   &  - \sum _{\begin{array}{c} k\in \mathbb {Z}^{3,N}_0\\ j\in \{1,2\} \end{array}}\mathcal {L}_{\sigma _{k,j}^{\varepsilon }}B^{\varepsilon }_t dW^{k,j}_t, \\ \operatorname {div}B^{\varepsilon }_t& =0, \\ B^{\varepsilon }_t|_{t=0}& =B^{\varepsilon }_0. \end{array}\right. } \end{aligned}$$

We are left to study $$\sum _{\begin{array}{c} k\in \mathbb {Z}^{3,N}_0\\ j\in \{1,2\} \end{array}}\mathcal {L}_{\sigma _{k,j}^{\varepsilon }}\mathcal {L}_{\sigma _{-k,j}^{\varepsilon }}+\rho \sum _{\begin{array}{c} k\in \mathbb {Z}^{3,N}_0\\ k_3=0 \end{array}}\mathcal {L}_{\sigma _{k,1}^{\varepsilon }}\mathcal {L}_{\sigma _{-k,2}^{\varepsilon }}+\mathcal {L}_{\sigma _{k,2}^{\varepsilon }}\mathcal {L}_{\sigma _{-k,1}^{\varepsilon }} $$. Its analysis exploits strongly the properties of the covariance function of our noise, i.e. translation invariance and (almost) mirror symmetry and it is contained in the following Lemma. Even if its proof is quite standard, see [12, Section 3.4.1] we prefer to include it in Appendix A for the convenience of the reader.

#### Lemma 2.12

If $F:\mathbb {T}^3_{\varepsilon }\rightarrow \mathbb {R}^3$ is a smooth, zero mean divergence free vector field, then it holds

$$\begin{aligned} \sum _{\begin{array}{c} k\in \mathbb {Z}^{3,N}_0\\ j\in \{1,2\} \end{array}}\mathcal {L}_{\sigma _{k,j}^{\varepsilon }}\mathcal {L}_{\sigma _{-k,j}^{\varepsilon }} F+\rho \sum _{\begin{array}{c} k\in \mathbb {Z}^{3,N}_0\\ k_3=0 \end{array}}\left( \mathcal {L}_{\sigma _{k,1}^{\varepsilon }}\mathcal {L}_{\sigma _{-k,2}^{\varepsilon }}+\mathcal {L}_{\sigma _{k,2}^{\varepsilon }}\mathcal {L}_{\sigma _{-k,1}^{\varepsilon }}\right) F&=\Lambda ^{\varepsilon }F+ \Lambda _{\rho }^{\varepsilon }F,\quad \end{aligned}$$

where in the formula above we denoted by $\Lambda ^{\varepsilon }$ the differential operator

$$\begin{aligned} \Lambda ^{\varepsilon }F=\eta ^{\varepsilon }_T(\partial ^2_{11}+\partial ^2_{22})F+\eta ^{\varepsilon }_R\partial ^2_{33}F \end{aligned}$$

and by $\Lambda _{\rho }^{\varepsilon }$ the differential operator

$$\begin{aligned} \Lambda _{\rho }^{\varepsilon }F=-\sum _{l\in \{1,2\}} \partial _l \overline{Q^{\varepsilon }_{\rho }}(0)\cdot \nabla F_l=-\sum _{\begin{array}{c} j\in \{1,2,3\}\\ l\in \{1,2\} \end{array}} \partial _l \overline{Q^{\varepsilon ,\cdot , j}_{\rho }}(0)\partial _jF_l. \end{aligned}$$

Thanks to Lemma 2.12, equation (2.25) can be interpreted in Itô form as:

2.26 $$\begin{aligned} {\left\{ \begin{array}{ll} dB^{\varepsilon }_{t}& =\left( \eta \Delta +\Lambda ^{\varepsilon }+\Lambda ^{\varepsilon }_{\rho }\right) B^{\varepsilon }_{t} dt - \sum _{\begin{array}{c} k\in \mathbb {Z}^{3,N}_0\\ j\in \{1,2\} \end{array}}\mathcal {L}_{\sigma _{k,j}^{\varepsilon }}B^{\varepsilon }_t dW^{k,j}_t, \\ \operatorname {div}B^{\varepsilon }_t& =0, \\ B^{\varepsilon }_t|_{t=0}& =B^{\varepsilon }_0, \end{array}\right. } \end{aligned}$$

We give now the definition of weak solution for the Stochastic equation of the magnetic field (2.26).

#### Definition 2.13

A stochastic process

$$\begin{aligned} B^{\varepsilon }\in C_{\mathcal {F}}([0,T];\textbf{L}^2_{\varepsilon })\cap L^2_{\mathcal {F}}([0,T];\textbf{H}^1_{\varepsilon }) \end{aligned}$$

is a weak solution of equation (2.26) if, for every $\phi \in \textbf{H}^2_{\varepsilon }$, we have

2.27 $$\begin{aligned} \langle B^{\varepsilon }_t,\phi \rangle _{\varepsilon }&= \langle B^{\varepsilon }_0,\phi \rangle _{\varepsilon }+ \eta \int _0^t \langle B^{\varepsilon }_s,\Delta \phi \rangle _{\varepsilon } \, ds+\int _0^t \langle B^{\varepsilon }_s,\Lambda ^{\varepsilon }\phi \rangle _{\varepsilon }\, ds \nonumber \\&\quad +\int _0^t \langle \Lambda ^{\varepsilon }_{\rho }B^{\varepsilon }_s,\phi \rangle _{\varepsilon }\, ds-\sum _{\begin{array}{c} k\in \mathbb {Z}^{3,N}_0\\ j\in \{1,2\} \end{array}}\int _0^t\langle \mathcal {L}_{\sigma _{k,j}^{\varepsilon }}B^{\varepsilon }_s,\phi \rangle _{\varepsilon } dW^{k,j}_s. \end{aligned}$$

for every $$t\in [0,T],\ \mathbb {P}-a.s.$$

Due to the fact that equation (2.26) is linear, the existence and uniqueness of solutions in the sense of Definition 2.13 is a standard fact which follows by the abstract theory of [5, Chapters 3-5], see also [11, Section 3.1]. Indeed the following holds.

#### Theorem 2.14

For each $B^{\varepsilon }_0\in \textbf{L}^2_{\varepsilon }$ there exists a unique weak solution of system (2.26) in the sense of Definition 2.13.

We are now ready to introduce the mean and the fluctuation in the third component of the process $B^{\varepsilon }$ which are the main objects of our analysis. Therefore, let

$$\begin{aligned} \overline{B^{\varepsilon }_{t}}:=M_{\varepsilon }B^{\varepsilon }_t,\ {{B}^{\varepsilon }_{t}}^{\prime }:=N_{\varepsilon }B^{\varepsilon }_t. \end{aligned}$$

More generally, in order to simplify the notation, in the following we will denote the projection operator $$M_{\varepsilon }$$ (resp. $$N_{\varepsilon }$$) by $$\overline{\cdot }$$ (resp. $\cdot^{\prime }$) forgetting the dependence on ε. Thanks to Theorem 2.14 and the fact that $$M_{\varepsilon }$$ and $$N_{\varepsilon }$$ are projection operator and commute with the operator $$\operatorname {div}$$, see Lemma 2.3, we have in particular that

$$\begin{aligned} \overline{B^{\varepsilon }}\in L^2_{\mathcal {F}}(0,T;\textbf{H}^1_{\varepsilon })\cap C_{\mathcal {F}}(0,T;\textbf{L}^2_{\varepsilon }),\quad {{B}^{\varepsilon }}'\in L^2_{\mathcal {F}}(0,T;\textbf{H}^1_{\varepsilon })\cap C_{\mathcal {F}}(0,T;\textbf{L}^2_{\varepsilon }).\end{aligned}$$

For each $$\phi \in \textbf{H}^2_{\varepsilon }$$, considering $$\overline{\phi }$$ and $\phi^{\prime }$ as test function in (2.27) we obtain that $$\overline{B^{\varepsilon }_{t}}$$ and $${{B}^{\varepsilon }_{t}}^{\prime }$$ are weak solutions of the following SPDEs:

2.28 $$\begin{aligned} {\left\{ \begin{array}{ll} d\overline{B^{\varepsilon }_{t}}& =\left( \eta \Delta +\Lambda ^{\varepsilon }+\Lambda ^{\varepsilon }_{\rho }\right) \overline{B^{\varepsilon }_{t}} dt - \sum _{\begin{array}{c} k\in \mathbb {Z}^{3,N}_0\\ k_3=0,\quad j\in \{1,2\} \end{array}}\mathcal {L}_{\sigma _{k,j}^{\varepsilon }}\overline{B^{\varepsilon }_{t}} dW^{k,j}_t\\   &  -\sum _{\begin{array}{c} k\in \mathbb {Z}^{3,N}_0\\ k_3\ne 0,\quad j\in \{1,2\} \end{array}}\overline{\mathcal {L}_{\sigma _{k,j}^{\varepsilon }}{{B}^{\varepsilon }_{t}}^{\prime }} dW^{k,j}_t, \\ \operatorname {div}\overline{B^{\varepsilon }_{t}}& =0, \\ \overline{B^{\varepsilon }_{t}}|_{t=0}& =\overline{B^{\varepsilon }_{0}}, \end{array}\right. } \end{aligned}$$

2.29 $$\begin{aligned} {\left\{ \begin{array}{ll} d{{B}^{\varepsilon }_{t}}^{\prime }& =\left( \eta \Delta +\Lambda ^{\varepsilon }+\Lambda ^{\varepsilon }_{\rho }\right) {{B}^{\varepsilon }_{t}}^{\prime } dt - \sum _{\begin{array}{c} k\in \mathbb {Z}^{3,N}_0\\ k_3=0,\quad j\in \{1,2\} \end{array}}\mathcal {L}_{\sigma _{k,j}^{\varepsilon }}{{B}^{\varepsilon }_{t}}^{\prime } dW^{k,j}_t\\   & -\sum _{\begin{array}{c} k\in \mathbb {Z}^{3,N}_0\\ k_3\ne 0,\quad j\in \{1,2\} \end{array}}\mathcal {L}_{\sigma _{k,j}^{\varepsilon }}\overline{B^{\varepsilon }_{t}} dW^{k,j}_t-\sum _{\begin{array}{c} k\in \mathbb {Z}^{3,N}_0\\ k_3\ne 0,\quad j\in \{1,2\} \end{array}}\left( \mathcal {L}_{\sigma _{k,j}^{\varepsilon }}{{B}^{\varepsilon }_{t}}^{\prime }\right) ' dW^{k,j}_t, \\ \operatorname {div}{{B}^{\varepsilon }_{t}}^{\prime }& =0, \\ {{B}^{\varepsilon }_{t}}^{\prime }|_{t=0}& ={{B}^{\varepsilon }_{0}}^{\prime } \end{array}\right. } \end{aligned}$$

in a sense completely analogous to Definition 2.13, namely testing the equations with vector fields $\phi \in \textbf{H}^2_{\varepsilon }$. As discussed in Section 1, we study the convergence of $\overline{B^{\varepsilon }_{t}}$ in the simultaneous scaling limit of both separation of scales, i.e. considering a noise which concentrates on smaller and smaller scales as described in subsection 2.3, and of the width of our thin layer, namely considering $\varepsilon =\frac{1}{N}\rightarrow 0$ as discussed in [24, 25, 28]. In order to do so, we need to introduce some few objects: we denote by

$$\begin{aligned} \overline{A^{\varepsilon }_{t}}=K_{\varepsilon }[\overline{B^{\varepsilon }_{t}}],\quad {{A}^{\varepsilon }_{t}}^{\prime }=K_{\varepsilon }[{{B}^{\varepsilon }_{t}}^{\prime }]. \end{aligned}$$

Thanks to the regularity properties of the Biot-Savart operator, see Subsubsection 2.2.1, we have that

$$\begin{aligned} \overline{A^{\varepsilon }}\in L^2_{\mathcal {F}}(0,T;\textbf{H}^2_{\varepsilon })\cap C_{\mathcal {F}}(0,T;\textbf{H}^1_{\varepsilon }),\quad {{A}^{\varepsilon }}'\in L^2_{\mathcal {F}}(0,T;\textbf{H}^2_{\varepsilon })\cap C_{\mathcal {F}}(0,T;\textbf{H}^1_{\varepsilon }), \end{aligned}$$

moreover

$$\begin{aligned} \overline{A^{\varepsilon }}=(\overline{A^{H,\varepsilon }}, \overline{A^{3,\varepsilon }})^t=(-\nabla _H^{\perp }(-\Delta )^{-1}\overline{B^{3,\varepsilon }},(-\Delta )^{-1}\nabla ^{\perp }_H\cdot \overline{B^{H,\varepsilon }})^t. \end{aligned}$$

Lastly we introduce our limit objects. Following the idea first introduced in [14], we expect that our limit objects satisfy a PDE with an additional second order operator with intensity related to

$$\begin{aligned} \operatorname {lim}_{\varepsilon \rightarrow 0}\sum _{\begin{array}{c} k\in \mathbb {Z}^{3,N}_0\\ j\in \{1,2\} \end{array}}\left\| \sigma _{k,j}^{\varepsilon }\right\| _{\varepsilon }^2. \end{aligned}$$

Besides to the fact that the limit above is 0, in the simultaneous separation of scale, thin layer limit, according to the discussion in subsection 2.3, the final deterministic objects keep memory of the operators $\Lambda ^{\varepsilon },\ \Lambda ^{\varepsilon }_{\rho }$ defined above. Indeed, denoting by $\overline{A^3},\ \overline{B^3}$ the unique weak solutions of the following linear 2D PDEs

2.30 $$\begin{aligned}&{\left\{ \begin{array}{ll} \partial _t \overline{A^{3,\gamma }_t}& =(\eta +\eta _T)\Delta \overline{A^{3,\gamma }_t}\quad x\in \mathbb {T}^2,\ t\in (0,T)\\ \overline{A^{3,\gamma }_t}|_{t=0}& =\overline{A^3_0}, \end{array}\right. }\end{aligned}$$

2.31 $$\begin{aligned}&{\left\{ \begin{array}{ll} \partial _t \overline{B^{3,\gamma }_t}& =(\eta +\eta _T)\Delta \overline{B^{3,\gamma }_t}+\operatorname {div}\left( \mathcal {R}_{\gamma }\nabla ^{\perp }\overline{A^{3,\gamma }_t}\right) \quad x\in \mathbb {T}^2,\ t\in (0,T)\\ \overline{B^{3,\gamma }_t}|_{t=0}& =\overline{B^3_0}, \end{array}\right. } \end{aligned}$$

where $$\eta _T=\frac{\zeta _{H,2}}{C_{1,H}^2}$$ and

$$\begin{aligned} \mathcal {R}_{\gamma }={\left\{ \begin{array}{ll} \frac{2\pi \rho \log {2}}{C_{1,H}C_{2,H}}\begin{bmatrix} 0 &  -1\\ 1 &  0 \end{bmatrix} &  \text {if } \gamma =4\\ 0 &  \text {if } \gamma >4, \end{array}\right. } \end{aligned}$$

our main result reads as follows:

#### Theorem 2.15

Assuming Hypothesis 2.6 and

2.32 $$\begin{aligned}&\overline{B^{3,\varepsilon }_{0}}\rightharpoonup \overline{B^3_0} \text { in }\dot{L}^2(\mathbb {T}^2),\quad \overline{A^{3,\varepsilon }_{0}}\rightharpoonup \overline{A^3_0} \text { in }\dot{L}^2(\mathbb {T}^2),\end{aligned}$$

2.33 $$\begin{aligned}&\quad \sup _{\varepsilon \in (0,1)}\frac{\left\| {{B}^{3,\varepsilon }_{0}}^{\prime }\right\| _{\varepsilon }^2}{\varepsilon },\quad \sup _{\varepsilon \in (0,1)}\frac{\left\| {{A}^{\varepsilon }_{0}}^{\prime }\right\| _{\varepsilon }^2}{\varepsilon }<+\infty , \end{aligned}$$

there exists $N_0$ large enough such that for each $N\ge N_0$, for each $\theta _1\in (0,1],\ \theta _2\in (0,\theta _1),\ \delta \in (0,\theta _2)$ we have

2.34 $$\begin{aligned}&\operatorname {sup}_{t\in [0,T]}\mathbb {E} \left[ \left\| \overline{B^{3,\varepsilon }_{t}}- \overline{B^{3,\gamma }_t}\right\| _{\dot{H}^{-\theta _1}}^2\right] \nonumber \\&\lesssim {\left\{ \begin{array}{ll} \left\| \overline{B^3_0}-\overline{B^{3,\varepsilon }_{0}}\right\| ^2_{\dot{H}^{-\theta _1}}+\left\| \overline{A^3_0}-\overline{A^{3,\varepsilon }_{0}}\right\| ^2_{\dot{H}^{-\theta _2}}+\dfrac{1}{ N^{\dfrac{(\theta _2-\delta )(\theta _1-\theta _2)}{(1+\delta )}}}\\ \text {if }\gamma =4,\\ \left\| \overline{B^3_0}-\overline{B^{3,\varepsilon }_{0}}\right\| ^2_{\dot{H}^{-\theta _1}}+\left\| \overline{A^3_0}-\overline{A^{3,\varepsilon }_{0}}\right\| ^2_{\dot{H}^{-\theta _2}}+\dfrac{1}{N^{\gamma -4}}+\frac{1}{ N^{\dfrac{(\theta _2-\delta )(\theta _1-\theta _2)}{(1+\delta )}}}\\ \text {if }\gamma >4; \end{array}\right. } \end{aligned}$$

2.35 $$\begin{aligned}&\operatorname {sup}_{t\in [0,T]}\mathbb {E} \left[ \left\| \overline{A^{3,\varepsilon }_{t}}-\overline{A^{3,\gamma }_t}\right\| _{\dot{H}^{-\theta _2}}^2\right] \lesssim \left\| \overline{A^{3,\varepsilon }_{0}}-\overline{A^3_0}\right\| _{\dot{H}^{-\theta _2}}^2+\frac{1}{N^{\theta _2}}+\dfrac{1}{ N^{\dfrac{2(\theta _2-\delta )}{1+\delta }}}. \end{aligned}$$

The hidden constants in (2.34), (2.35) depend on η, β, γ, $C_V,$ $C_{1,H},$ $C_{2,H},$ |ρ|, *T*, $\operatorname {sup}_{\varepsilon \in (0,1)}{\left\| \overline{B^{3,\varepsilon }_{0}}\right\| ^2},$ $\operatorname {sup}_{\varepsilon \in (0,1)}{\left\| \overline{A^{3,\varepsilon }_{0}}\right\| ^2}$, $\operatorname {sup}_{\varepsilon \in (0,1)}\frac{{\left\| {{B}^{3,\varepsilon }_{0}}^{\prime }\right\| ^2_{\varepsilon }}}{{\varepsilon }},$ $\operatorname {sup}_{\varepsilon \in (0,1)}\frac{{\left\| {{A}^{3,\varepsilon }_{0}}^{\prime }\right\| ^2_{\varepsilon }}}{{\varepsilon }},$ $\theta _1,$ $\theta _2$, δ. In particular

$$\begin{aligned}&\operatorname {sup}_{t\in [0,T]}\mathbb {E} \left[ \left\| \overline{B^{3,\varepsilon }_{t}}-\overline{B^{3,\gamma }_t}\right\| _{\dot{H}^{-\theta _1}}^2\right] +\operatorname {sup}_{t\in [0,T]}\mathbb {E} \left[ \left\| \overline{A^{3,\varepsilon }_{t}}-\overline{A^{3,\gamma }_t}\right\| _{\dot{H}^{-\theta _2}}^2\right] \rightarrow 0\quad \\  &\text {as } N\rightarrow +\infty . \end{aligned}$$

#### Remark 2.16

The explicit dependence of the hidden constants in (2.34), (2.35) in terms of $\theta _1,\ \theta _2$ and δ can be found in Section 4, see Subsection 4.5 and Subsection 4.6.

#### Remark 2.17

Our assumption (2.32) is for example satisfied if $B^{\varepsilon }_0$ can be decomposed as

$$\begin{aligned} B^{\varepsilon }_0(x_1,x_2,x_3)=\overline{B}_0(x_1,x_2)+{B}'_0\left( x_1,x_2,\frac{x_3}{\varepsilon }\right) , \end{aligned}$$

where, calling ${B^{\varepsilon }_0}'=B_0'\left( \cdot ,\cdot ,\frac{\cdot }{\varepsilon }\right)$, we have

$$\begin{aligned}&\overline{B}_0\in \dot{L}^2(\mathbb {T}^2; \mathbb {R}^3),\quad {B}'_0\in \dot{L}^2(\mathbb {T}^3; \mathbb {R}^3),\quad \int _{\mathbb {T}} B'_0(x_1,x_2,x_3) dx_3=0\ a.e.\ (x_1,x_2)\in \mathbb {T}^2\\&\operatorname {div} \overline{B}_0=0,\quad \operatorname {div}{B^{\varepsilon }_0}'=0\quad \forall N\in \mathbb {N}. \end{aligned}$$

#### Remark 2.18

Assuming $\overline{A^3_0}\in \dot{H}^1_0$, simple energy estimates applied to equation (2.30) imply

$$\begin{aligned} \left\| \overline{A^{3,\gamma }_t}\right\| _{\dot{H}^1(\mathbb {T}^2)}&\le e^{-(\eta +\eta _T)t}\left\| \overline{A^3_0}\right\| _{\dot{H}^1(\mathbb {T}^2)}. \end{aligned}$$

Consequently energy estimates applied to equation (2.31) imply by Young’s inequality

$$\begin{aligned} \left\| \overline{B^{3,\gamma }_t}\right\| ^2+2(\eta +\eta _T)\int _0^t \left\| \nabla \overline{B^{3,\gamma }_s}\right\| ^2ds&\lesssim \left\| \overline{B^{3,\gamma }_0}\right\| ^2+\left\| \overline{A^3_0}\right\| _{\dot{H}^1(\mathbb {T}^2)}\int _0^t \left\| \overline{B^{3,\gamma }_s}\right\| e^{-(\eta +\eta _T)s}ds\\  &\le C\left( \left\| \overline{B^{3,\gamma }_0}\right\| ^2+\left\| \overline{A^3_0}\right\| _{\dot{H}^1(\mathbb {T}^2)}^2\right) \\  &\quad +(\eta +\eta _T)\int _0^t \left\| \nabla \overline{B^{3,\gamma }_s}\right\| ^2ds, \end{aligned}$$

for some constant *C* depending on the parameters of the model. Therefore Grönwall’s inequality implies

$$\begin{aligned} \left\| \overline{B^{3,\gamma }_t}\right\|&\lesssim e^{-\frac{1}{2}(\eta +\eta _T)t}\left\| \overline{B^3_0}\right\| . \end{aligned}$$

In conclusion, calling $\overline{B^{H,\gamma }}=-\nabla ^{\perp }\overline{A^{3,\gamma }}$, the full magnetic field associated to the limit mean field model, $\overline{B^\gamma }=(\overline{B^{H,\gamma }},\overline{B^{3,\gamma }})$ decays exponentially, i.e.

$$\begin{aligned} \left\| \overline{B^{\gamma }_t}\right\|&\lesssim e^{-\frac{1}{2}(\eta +\eta _T)t}\left\| \overline{B^{\gamma }_0}\right\| . \end{aligned}$$

In this sense our main result, Theorem 2.15, corresponds to a result of no-dynamo.

#### Remark 2.19

Due to Hypothesis 2.6, $\eta _T$ cannot be arbitrarily large in our model. Indeed the following holds

$$\begin{aligned} \eta _T=\frac{\zeta _{H,2}}{C_{1,H}^2}<\frac{\zeta _{H,2}}{\zeta _{H,0}}\eta \approx 0.462 \eta . \end{aligned}$$

#### Remark 2.20

Contrary to other results related to ours, see [7, 9, 10, 14, 28], we do not argue by compactness but we provide a quantitative result in the original probability space. Besides the fact that this makes the result stronger, we are forced to argue in this way. Indeed, we can obtain uniform estimates in a common topology only for $\overline{B^{3,\varepsilon }_{\cdot }},\ \overline{A^{3,\varepsilon }_{\cdot }}$ exploiting the fact that they are independent of the third variable and we can see them as functions from $\mathbb {T}^2$ to $\mathbb {R}^3$. This seems not possible for quantities related to ${{B}^{\varepsilon }_{\cdot }}^{\prime }$. Due to the lack of compactness for quantities related to ${{B}^{\varepsilon }_{\cdot }}^{\prime }$, we cannot find by Skorokhod’s representation theorem an auxiliary probability space where either (2.28), (2.29) hold and $(\overline{B^{3,\varepsilon }_{\cdot }},\ \overline{A^{3,\varepsilon }_{\cdot }})$ converge a.s. to some limit objects. Therefore we cannot pass to the limit in equation (2.28) showing that the limit objects satisfy (2.30), (2.31).

### Strategy of the proof

The proof of Theorem 2.15 relies on rewriting in mild form $\overline{B^{3,\varepsilon }_{t}},\ \overline{A^{3,\varepsilon }_{t}},\ \overline{M^3_t},\ \overline{A^3_t}$, see Lemma 4.1, showing, in a quantitative way, that the stochastic convolutions approach 0 when considering negative Sobolev norms in $\mathbb {T}^2$ and having a good control on the pieces associated to the alpha-term in (2.28), (2.31). This is the object of Corollary 4.4, Subsection 4.5 and Subsection 4.6. These results (and actually also stronger ones) would be easily true if the following holds: If $\left\| B_0^{\varepsilon }\right\| _{\varepsilon }^2=O(\varepsilon )$, then also

2.36 $$\begin{aligned} \left\| {B^{\varepsilon }}\right\| _{C_{\mathcal {F}}(0,T;\textbf{L}^2_{\varepsilon })}^2+\left\| {B^{\varepsilon }}\right\| _{L^2_{\mathcal {F}}(0,T;\textbf{H}^1_{\varepsilon })}^2=O(\varepsilon ). \end{aligned}$$

Unfortunately, due to the stochastic stretching terms, equation (2.36) seems completely out of reach if one is interested in having a non-trivial operator in the limit $\operatorname {lim}_{\varepsilon \rightarrow 0}\Lambda ^{\varepsilon }$, see [8, Appendix 2]. Even if the operator $\Lambda ^{\varepsilon }_{\rho }$ and the correlation between the components of the noise $W^{\varepsilon }_t$ makes system (2.26) more complicated, they do not increase the difficulties in order to obtain (2.36). We rely on the following idea, which we inherited by [25, 28]: since the equation for $\overline{B^{\varepsilon }_{t}}$ is a perturbation of a 2D-3C system, we can work, as in [9], considering differently the horizontal and the vertical component of the magnetic field $\overline{B^{\varepsilon }_{t}}$. Since the third component $\overline{B^{3,\varepsilon }_{t}}$ corresponds, in a vague sense, to the unique non null component associated to the $\operatorname {curl}$ of a 2D divergence free vector field, according to [7, 6, 10], we expect that, if we were able to control the perturbation with respect to the 2D-3C model, it behaves well in terms of (2.36), see [9]. This is not true when considering $\overline{B^{H,\varepsilon }_{t}}$. Indeed, having a good control on the stochastic stretching terms appearing in the equation of $\overline{B^{H,\varepsilon }_{t}}$ is as difficult as proving (2.36) and keeping a non-trivial operator in the limit due to the fact that, without exploiting some geometric features of the model, the term

2.37 $$\begin{aligned} \sum _{\begin{array}{c} k\in \mathbb {Z}^{3,N}_0\\ k_3=0,\quad j\in \{1,2\} \end{array}}\int _0^T \left\| \overline{B^{\varepsilon }_{s}}\cdot \nabla \sigma _{k,j}^{H,\varepsilon }\right\| _{\varepsilon }^2 ds, \end{aligned}$$

appearing in the energy estimates, seems to blow-up. The idea is, therefore, of considering the potential field $\overline{A^{\varepsilon }_{t}}$ associated to $\overline{B^{\varepsilon }_{t}}$. Thanks to (2.2), $\overline{B^{H,\varepsilon }_{t}}=-\nabla ^{\perp }_H \overline{A^{3,\varepsilon }_{t}}$ and, formally, due to Proposition 3.2 below, it solves

$$\begin{aligned} d\overline{A^{3,\varepsilon }_{t}}=\,&\left( \eta \Delta +\Lambda ^{\varepsilon }\right) \overline{A^{3,\varepsilon }_{t}} dt - \sum _{\begin{array}{c} k\in \mathbb {Z}^{3,N}_0\\ k_3=0,\quad j\in \{1,2\} \end{array}}\sigma _{k,j}^{\varepsilon }\cdot \nabla \overline{A^{3,\varepsilon }_{t}} dW^{k,j}_t\\  &-\sum _{\begin{array}{c} k\in \mathbb {Z}^{3,N}_0\\ k_3\ne 0,\quad j\in \{1,2\} \end{array}}Q\left[ \overline{\sigma _{k,j}^{\varepsilon }\cdot \nabla {{A}^{3,\varepsilon }_{t}}^{\prime }+\partial _3\sigma _{k,j}^{\varepsilon }\cdot {{A}^{3,\varepsilon }_{t}}^{\prime }} \right] dW^{k,j}_t. \end{aligned}$$

Notice that, due to Remark A.1, $\Lambda ^{\varepsilon }_{\rho }\overline{B^{\varepsilon }_{t}}$ is a vector having the first two components null, therefore it does not affect the equation for $\overline{B^{H,\varepsilon }_{t}}$. Now, the bad stretching term (2.37) has disappeared at this level of regularity, and to show that $\overline{A^{3,\varepsilon }_{t}}$ behaves well in term of (2.36) we are left to analyse this perturbation of the 2D-3C model. In order to show that the equations for $\overline{A^{3,\varepsilon }_{t}},\ \overline{B^{3,\varepsilon }_{t}}$ are just a regular perturbation of the 2D-3C model we need either some smallness assumptions on the coefficient $\sigma _{k,j}^{\varepsilon },\ k_3\ne 0$ in term of the derivative of the covariance function associated to the noise, see subsection 2.3, and some bounds on ${{B}^{\varepsilon }_{t}}^{\prime }$. Also in this case, the analysis of ${{B}^{3,\varepsilon }_{t}}^{\prime }$ is relatively easy thanks to our assumptions on the noise which allow us to control the stochastic stretching terms, see subsection 2.3. Lastly we need a good control on ${{B}^{H,\varepsilon }_{t}}^{\prime }$. This term seems again critical for what concerns the stochastic stretching, indeed a term of the form

2.38 $$\begin{aligned} \sum _{\begin{array}{c} k\in \mathbb {Z}^{3,N}_0\\ k_3=0,\quad j\in \{1,2\} \end{array}}\int _0^T \left\| {{B}^{\varepsilon }_{s}}^{\prime }\cdot \nabla \sigma _{k,j}^{H,\varepsilon }\right\| _{\varepsilon }^2 ds \end{aligned}$$

appears when computing energy estimates. Besides its form analogous to (2.37), the term (2.38) needs a different treatment than the others. For this term, we are able to exploit the special geometry of the thin layer, namely the Poincaré inequality (2.6) (with constant ε) holds when considering ${{B}^{\varepsilon }_{t}}^{\prime }$, counterbalancing the gradients of the coefficients. Indeed, applying Hölder’s inequality to relation (2.38), the explosive behaviour of $\left\| \nabla \sigma _{k,j}^{H,\varepsilon }\right\| _{L^{\infty }(\mathbb {T}^3_{\varepsilon })}$ is absorbed thanks to (2.6) and we obtain

$$\begin{aligned} \sum _{\begin{array}{c} k\in \mathbb {Z}^{3,N}_0\\ k_3=0,\quad j\in \{1,2\} \end{array}}\int _0^T \left\| {{B}^{\varepsilon }_{s}}^{\prime }\cdot \nabla \sigma _{k,j}^{H,\varepsilon }\right\| _{\varepsilon }^2 ds&\le \left( \frac{\zeta _{H,0}}{C_{1,H}^2}+o(1)\right) \int _0^T \left\| \nabla {{B}^{\varepsilon }_{s}}^{\prime }\right\| ^2_{\varepsilon }ds. \end{aligned}$$

We refer to the analysis of $I^2_t$ in Lemma 3.3 below for details. Since we already have good estimates on $\int _0^T\left\| \nabla {{B}^{3,\varepsilon }_{s}}^{\prime }\right\| ^2_{\varepsilon }ds$ and Hypothesis 2.6 holds, (2.38) can be absorbed thanks to the dissipation properties of (2.29) obtaining a good control on the stochastic stretching term. Unfortunately this is not enough: energy estimates for ${{B}^{H,\varepsilon }_{t}}^{\prime }$ involves also terms related to $\int _0^T \left\| \nabla \overline{B^{H,\varepsilon }_{t}}\right\| _{\varepsilon }^2 ds$ which are out of reach as discussed before. For this reason, we replace the analysis of ${{B}^{H,\varepsilon }_{t}}^{\prime }$ with that of a different term, involving at most $\int _0^T\left\| \nabla \overline{B^{3,\varepsilon }_{t}}\right\| _{\varepsilon }^2 ds$ and $\int _0^T\left\| \nabla \overline{A^{3,\varepsilon }_{t}}\right\| _{\varepsilon }^2 ds$, when computing its energy estimates. This term, combined with ${{B}^{3,\varepsilon }_{t}}^{\prime }$, must be strong enough to ensure that $\overline{B^{3,\varepsilon }_{t}}$ and $\overline{A^{3,\varepsilon }_{t}}$ are close to satisfying the 2D-3C model. The appropriate choice for our purposes is ${{A}^{\varepsilon }_{t}}^{\prime }=K_{\varepsilon }[{{B}^{\varepsilon }_{t}}^{\prime }]$. The stochastic stretching term appearing in this case is not identically zero as for $\overline{A^{3,\varepsilon }_{t}}$, but can be treated as for (2.38).

Compared to [9], the equations appearing in our system are more complicated since, even if linear, none of them is closed and they are interconnected in a not trivial way when computing a priori estimates. This is only partially due to the presence of the operator $\Lambda ^{\varepsilon }_{\rho }$ and the correlation between the components of the noise $W^{\varepsilon }_t$. We hope that this subsection could help the reader in order to understand the reasons behind the long computations of Section 3 and the heuristics for Theorem 2.15.

## A priori estimates

Recalling that

$$\begin{aligned} \overline{B}^{\varepsilon }\in L^2_{\mathcal {F}}(0,T;\textbf{H}^1_{\varepsilon })\cap C_{\mathcal {F}}(0,T;\textbf{L}^2_{\varepsilon })\quad \text {(resp. }{B^{\varepsilon }}'\in L^2_{\mathcal {F}}(0,T;\textbf{H}^1_{\varepsilon })\cap C_{\mathcal {F}}(0,T;\textbf{L}^2_{\varepsilon })\text {),} \end{aligned}$$

since $\overline{B}^{\varepsilon }$ is independent of the third variable we have

$$\begin{aligned} \overline{B}^{\varepsilon }\in L^2_{\mathcal {F}}(0,T;\dot{H}^1(\mathbb {T}^2;\mathbb {R}^3))\cap C_{\mathcal {F}}(0,T;\dot{L}^2(\mathbb {T}^2;\mathbb {R}^3)).\end{aligned}$$

The goal of Corollary 3.6 is to prove some bounds uniform in ε>0 for quantities related to $\overline{B}^{\varepsilon }$ in a common topology independent of ε. In order to do so, we start obtaining in Lemma 3.3 and Lemma 3.4 some pathwise estimates for quantities related to $\overline{B}^{\varepsilon }$ and ${B^{\varepsilon }}'$. Thanks to these we are able to gain in Proposition 3.5 some intermediate a priori bounds in topologies depending on ε. These results will imply Corollary 3.6.

As discussed in Section 2.5 we will work with the potential fields associated to $\overline{B^{\varepsilon }_{t}}$ and ${{B}^{\varepsilon }_{t}}^{\prime }$ at a certain point. In order to do so we employ the following classical relations:

### Proposition 3.1

Let $$X\in \textbf{L}^2_{\varepsilon },\ Y\in \textbf{H}^1_{\varepsilon }$$. Then

3.1 $$\begin{aligned} \left\| X\right\| ^2_{\varepsilon }&=\left\| (-\Delta )^{-1/2}\operatorname {curl}X\right\| _{\varepsilon }^2,\quad \left\| \nabla Y\right\| ^2_{\varepsilon }=\left\| \operatorname {curl}Y\right\| _{\varepsilon }^2. \end{aligned}$$

In particular if either *X* and *Y* are independent of the third variable, it holds

3.2 $$\begin{aligned} \left\| X^3\right\| ^2_{\varepsilon }&=\left\| (-\Delta )^{-1/2}\nabla _{H}^{\perp } X^3\right\| _{\varepsilon }^2,\quad \left\| \nabla Y^3\right\| ^2_{\varepsilon }=\left\| \nabla _H^{\perp }Y^3\right\| _{\varepsilon }^2. \end{aligned}$$

### Proposition 3.2

Let $X:\mathbb {T}^3_{\varepsilon }\rightarrow \mathbb {R}^3$ a smooth, zero mean, divergence free vector field, $Y\in \textbf{H}^1_{\varepsilon }$. Then

3.3 $$\begin{aligned} X\cdot \nabla Y-Y\cdot \nabla X\in \textbf{L}^2_{\varepsilon }. \end{aligned}$$

Moreover it holds

3.4 $$\begin{aligned} X\cdot \nabla Y-Y\cdot \nabla X&=\operatorname {curl}\left( X\cdot \nabla K_{\varepsilon }[Y]+(DX)K_{\varepsilon }[Y]\right) \nonumber \\  &= \operatorname {curl}\left( Q\left[ X\cdot \nabla K_{\varepsilon }[Y]+(DX)K_{\varepsilon }[Y]\right] \right) \nonumber \\  &= \operatorname {curl}\left( P_{\varepsilon }\left[ Q\left[ X\cdot \nabla K_{\varepsilon }[Y]+(DX)K_{\varepsilon }[Y]\right] \right] \right) . \end{aligned}$$

In particular if both *X* and *Y* are independent of the third variable, it holds

3.5 $$\begin{aligned} X\cdot \nabla Y^H-Y\cdot \nabla X^H=-\nabla ^{\perp }_H\left( X\cdot \nabla K^3_{\varepsilon }[Y]\right) , \end{aligned}$$

where we denoted by $K^3_{\varepsilon }[Y]$ the third component of $K_{\varepsilon }[Y]$.

Now we have all the elements to provide the necessary a priori estimates.

### Lemma 3.3

Assuming Hypothesis 2.6, for each $\xi _1,\ \xi _2>0$ we have

3.6 $$\begin{aligned} d\left\| {{A}^{\varepsilon }_{t}}^{\prime }\right\| _{\varepsilon }^2+2\eta \left\| \nabla {{A}^{\varepsilon }_{t}}^{\prime }\right\| ^2_{\varepsilon }dt&\le -2\sum _{\begin{array}{c} k\in \mathbb {Z}^{3,N}_0\\ j\in \{1,2\} \end{array}}\langle D\sigma _{k,j}^{\varepsilon }{{A}^{\varepsilon }_{t}}^{\prime },{{A}^{\varepsilon }_{t}}^{\prime } \rangle _{\varepsilon }dW^{k,j}_t \nonumber \\  &\quad -2\sum _{\begin{array}{c} k\in \mathbb {Z}^{3,N}_0\\ k_3\ne 0,\quad j\in \{1,2\} \end{array}}\langle \sigma _{k,j}^{\varepsilon }\cdot \nabla \overline{A^{\varepsilon }_{t}}+D\sigma _{k,j}^{\varepsilon }\overline{A^{\varepsilon }_{t}},{{A}^{\varepsilon }_{t}}^{\prime } \rangle _{\varepsilon }dW^{k,j}_t \nonumber \\  &\quad {+}2\left( \frac{\zeta _{H,0}}{C_{1,H}^2}+\frac{|\rho |}{\xi _1}\frac{\left( \zeta _{H,0}{+}\zeta _{H,2}\right) }{C_{1,H}^2}+O(N^{-1})\right) \left\| \nabla {{A}^{\varepsilon }_{t}}^{\prime }\right\| ^2_{\varepsilon }dt\nonumber \\  &\quad +\left( 6\frac{\zeta _{\beta }\zeta _{H,0} }{N^{\beta -2}C_V^2}+O(N^{1-\beta })\right) \Vert \nabla \overline{A^{\varepsilon }_{t}}\Vert _{\varepsilon }^2 dt \nonumber \\  &\quad +\left( 6\frac{\zeta _{\beta -2}\zeta _{H,0}}{N^{\beta -4}C_V^2}+O(N^{3-\beta })\right) \left\| \overline{A^{\varepsilon }_{t}}\right\| _{\varepsilon }^2 dt, \end{aligned}$$

3.7 $$\begin{aligned}&d\left\| {{B}^{3,\varepsilon }_{t}}^{\prime }\right\| ^2_{\varepsilon }+2\eta \left\| \nabla {{B}^{3,\varepsilon }_{t}}^{\prime }\right\| ^2_{\varepsilon }dt\nonumber \\  &\le 2\sum _{\begin{array}{c} k\in \mathbb {Z}^{3,N}_0\\ j\in \{1,2\} \end{array}} \langle {{B}^{\varepsilon }_{t}}^{\prime }\cdot \nabla \sigma _{k,j}^{3,\varepsilon },{{B}^{3,\varepsilon }_{t}}^{\prime } \rangle _{\varepsilon }dW^{k,j}_t \nonumber \\  &\quad -2\sum _{\begin{array}{c} k\in \mathbb {Z}^{3,N}_0\\ k_3\ne 0,\quad j\in \{1,2\} \end{array}}\langle \sigma _{k,j}^{\varepsilon }\cdot \nabla \overline{B^{3,\varepsilon }_{t}}-\overline{B^{\varepsilon }_{t}}\cdot \nabla \sigma _{k,j}^{3,\varepsilon },{{B}^{3,\varepsilon }_{t}}^{\prime } \rangle _{\varepsilon }dW^{k,j}_t\nonumber \\  &\quad +2\frac{|\rho |}{\xi _2}\left( \frac{\zeta _{H,2}}{ C_{1,H}^2}+\sqrt{2}\frac{\zeta _{H,\gamma /2}}{C_{1,H}C_{2,H}}+O(N^{-1})\right) \left\| \nabla {{B}^{3,\varepsilon }_{t}}^{\prime }\right\| _{\varepsilon }^2 dt \nonumber \\  &\quad +2\left( \frac{\zeta _{H,\gamma -2}}{N^{\gamma -4}C_{2,H}^2}+2\frac{\zeta _{\beta -2}\zeta _{H,0}}{N^{\beta -4}C_V^2}+\frac{|\rho |\xi _2\zeta _{H,\gamma -2}}{N^{\gamma -4}C_{2,H}^2}+\sqrt{2}|\rho |\xi _2\frac{\zeta _{H,\gamma /2}}{N^{\gamma -4}C_{1,H}C_{2,H}}\right. \nonumber \\  &\left. \quad +O(N^{3-\gamma }+N^{3-\beta })\right) \left\| \nabla {{A}^{\varepsilon }_{t}}^{\prime }\right\| ^2_{\varepsilon }dt\nonumber \\  &\quad +2\sum _{\begin{array}{c} k\in \mathbb {Z}^{3,N}_0\\ k_3\ne 0,\quad j\in \{1,2\} \end{array}}\left\| \sigma _{k,j}^{\varepsilon }\cdot \nabla \overline{B^{3,\varepsilon }_{t}}\right\| ^2_{\varepsilon }dt+\left( 4\frac{\zeta _{\beta -2}\zeta _{H,0}}{N^{\beta -4}C_{V}^2}+O(N^{3-\beta })\right) \left\| \overline{B^{\varepsilon }_{t}}\right\| ^2_{\varepsilon }dt\nonumber \\  &\quad -2\sum _{\begin{array}{c} k\in \mathbb {Z}^{3,N}_0\\ k_3\ne 0,\quad j\in \{1,2\} \end{array}}\left\| \overline{\sigma _{k,j}^{\varepsilon }\cdot \nabla {{B}^{3,\varepsilon }_{t}}^{\prime }-{{B}^{\varepsilon }_{t}}^{\prime }\cdot \nabla \sigma _{k,j}^{3,\varepsilon }}\right\| _{\varepsilon }^2 dt. \end{aligned}$$

### Proof

Since by Proposition 3.1 it holds3.8we apply Itô formula to  obtaining

$$\begin{aligned} d\left\| {{A}^{\varepsilon }_{t}}^{\prime }\right\| ^2_{\varepsilon } = -dW^{\prime }_t+2(-I_t^1+I_t^2+I_t^3+I_t^4+I^5_t+I^6_t)dt, \end{aligned}$$

where we denote by

$$\begin{aligned} dW'_t&=2\sum _{\begin{array}{c} k\in \mathbb {Z}^{3,N}_0\\ k_3=0,\quad j\in \{1,2\} \end{array}}\langle \mathcal {L}_{\sigma _{k,j}^{\varepsilon }} {{B}^{\varepsilon }_{t}}^{\prime },(-\Delta )^{-1}{{B}^{\varepsilon }_{t}}^{\prime } \rangle _{\varepsilon }dW^{k,j}_t \\  &\quad +2\sum _{\begin{array}{c} k\in \mathbb {Z}^{3,N}_0\\ k_3\ne 0,\quad j\in \{1,2\} \end{array}}\langle \mathcal {L}_{\sigma _{k,j}^{\varepsilon }} \overline{B^{\varepsilon }_{t}},(-\Delta )^{-1}{{B}^{\varepsilon }_{t}}^{\prime } \rangle _{\varepsilon }dW^{k,j}_t \\&\quad +2\sum _{\begin{array}{c} k\in \mathbb {Z}^{3,N}_0\\ k_3\ne 0,\quad j\in \{1,2\} \end{array}}\langle \left( \mathcal {L}_{\sigma _{k,j}^{\varepsilon }}{{B}^{\varepsilon }_{t}}^{\prime }\right) ',(-\Delta )^{-1}{{B}^{\varepsilon }_{t}}^{\prime } \rangle _{\varepsilon }dW^{k,j}_t,\\ I^1_t&=\eta \langle \nabla {{B}^{\varepsilon }_{t}}^{\prime },(-\Delta )^{-1}\nabla {{B}^{\varepsilon }_{t}}^{\prime }\rangle _{\varepsilon }+\sum _{\begin{array}{c} k\in \mathbb {Z}^{3,N}_0\\ j\in \{1,2\} \end{array}}\langle {{B}^{\varepsilon }_{t}}^{\prime }, (-\Delta )^{-1}\operatorname {div}(\sigma _{k,j}^{\varepsilon }\otimes \sigma _{-k,j}^{\varepsilon }\nabla {{B}^{\varepsilon }_{t}}^{\prime })\rangle _{\varepsilon },\\ I^2_t&=\sum _{\begin{array}{c} k\in \mathbb {Z}^{3,N}_0\\ k_3=0,\quad j\in \{1,2\} \end{array}}\langle \mathcal {L}_{\sigma _{k,j}^{\varepsilon }}{{B}^{\varepsilon }_{t}}^{\prime }, (-\Delta )^{-1}\mathcal {L}_{\sigma _{-k,j}^{\varepsilon }}{{B}^{\varepsilon }_{t}}^{\prime }\rangle _{\varepsilon }, \\ I^3_t&= \sum _{\begin{array}{c} k\in \mathbb {Z}^{3,N}_0\\ k_3\ne 0,\quad j\in \{1,2\} \end{array}}\langle \mathcal {L}_{\sigma _{k,j}^{\varepsilon }} \overline{B^{\varepsilon }_{t}}, (-\Delta )^{-1}\mathcal {L}_{\sigma _{-k,j}^{\varepsilon }}\overline{B^{\varepsilon }_{t}}\rangle _{\varepsilon }, \\ I^4_t&=\sum _{\begin{array}{c} k\in \mathbb {Z}^{3,N}_0\\ k_3\ne 0,\quad j\in \{1,2\} \end{array}}\langle \left( \mathcal {L}_{\sigma _{k,j}^{\varepsilon }}{{B}^{\varepsilon }_{t}}^{\prime }\right) ', (-\Delta )^{-1}\left( \mathcal {L}_{\sigma _{-k,j}^{\varepsilon }}{{B}^{\varepsilon }_{t}}^{\prime }\right) '\rangle _{\varepsilon }, \\ I^5_t&=\sum _{\begin{array}{c} k\in \mathbb {Z}^{3,N}_0\\ k_3\ne 0,\quad j\in \{1,2\} \end{array}}\langle \mathcal {L}_{\sigma _{k,j}^{\varepsilon }} \overline{B^{\varepsilon }_{t}}, (-\Delta )^{-1}\left( \mathcal {L}_{\sigma _{-k,j}^{\varepsilon }}{{B}^{\varepsilon }_{t}}^{\prime }\right) '\rangle _{\varepsilon },\\ I_t^6&=\langle \Lambda ^{\varepsilon }_{\rho }{{B}^{\varepsilon }_{t}}^{\prime },(-\Delta )^{-1}{{B}^{\varepsilon }_{t}}^{\prime }\rangle _{\varepsilon }+\rho \sum _{\begin{array}{c} k\in \mathbb {Z}^{3,N}_0\\ k_3=0 \end{array}}\langle \mathcal {L}_{\sigma _{k,1}^{\varepsilon }}{{B}^{\varepsilon }_{t}}^{\prime },(-\Delta )^{-1}\mathcal {L}_{\sigma _{-k,2}^{\varepsilon }} {{B}^{\varepsilon }_{t}}^{\prime }\rangle _{\varepsilon }\\  &\quad +\langle \mathcal {L}_{\sigma _{k,2}^{\varepsilon }}{{B}^{\varepsilon }_{t}}^{\prime },(-\Delta )^{-1}\mathcal {L}_{\sigma _{-k,1}^{\varepsilon }}{{B}^{\varepsilon }_{t}}^{\prime }\rangle _{\varepsilon }. \end{aligned}$$

In order to study the terms involved in previous relation we recall that by Proposition 3.2

$$\begin{aligned} \operatorname {curl}(\sigma _{k,j}^{\varepsilon }\cdot \nabla {{A}^{\varepsilon }_{t}}^{\prime }+D\sigma _{k,j}^{\varepsilon }{{A}^{\varepsilon }_{t}}^{\prime })=\sigma _{k,j}^{\varepsilon }\cdot \nabla {{B}^{\varepsilon }_{t}}^{\prime }-{{B}^{\varepsilon }_{t}}^{\prime }\cdot \nabla \sigma _{k,j}^{\varepsilon }\end{aligned}$$

and analogously for the others. First we start rewriting the martingale term. By previous relation, exploiting the fact that $\operatorname {curl}\operatorname {curl}f=-\Delta f$ if *f* is a divergence free vector field and derivatives and the operator $(-\Delta )^{-1}$ commute in the periodic framework we get

3.9 $$\begin{aligned} dW'_t&= 2\sum _{\begin{array}{c} k\in \mathbb {Z}^{3,N}_0\\ j\in \{1,2\} \end{array}}\langle \mathcal {L}_{\sigma _{k,j}^{\varepsilon }}{{B}^{\varepsilon }_{t}}^{\prime },(-\Delta )^{-1}{{B}^{\varepsilon }_{t}}^{\prime } \rangle _{\varepsilon }dW^{k,j}_t \nonumber \\  &\quad +2\sum _{\begin{array}{c} k\in \mathbb {Z}^{3,N}_0\\ k_3\ne 0,\quad j\in \{1,2\} \end{array}}\langle \mathcal {L}_{\sigma _{k,j}^{\varepsilon }} \overline{B^{\varepsilon }_{t}},(-\Delta )^{-1}{{B}^{\varepsilon }_{t}}^{\prime } \rangle _{\varepsilon }dW^{k,j}_t \nonumber \\  &= 2\sum _{\begin{array}{c} k\in \mathbb {Z}^{3,N}_0\\ j\in \{1,2\} \end{array}}\langle \operatorname {curl}\left( \sigma _{k,j}^{\varepsilon }\cdot \nabla {{A}^{\varepsilon }_{t}}^{\prime }+D\sigma _{k,j}^{\varepsilon }{{A}^{\varepsilon }_{t}}^{\prime }\right) ,(-\Delta )^{-1}\operatorname {curl}{{A}^{\varepsilon }_{t}}^{\prime } \rangle _{\varepsilon }dW^{k,j}_t \nonumber \\&\quad +2\sum _{\begin{array}{c} k\in \mathbb {Z}^{3,N}_0\\ k_3\ne 0,\quad j\in \{1,2\} \end{array}}\langle \operatorname {curl}\left( \sigma _{k,j}^{\varepsilon }\cdot \nabla \overline{A^{\varepsilon }_{t}}+D\sigma _{k,j}^{\varepsilon }\overline{A^{\varepsilon }_{t}}\right) ,(-\Delta )^{-1}\operatorname {curl}{{A}^{\varepsilon }_{t}}^{\prime } \rangle _{\varepsilon }dW^{k,j}_t \nonumber \\  &=2\left( \sum _{\begin{array}{c} k\in \mathbb {Z}^{3,N}_0\\ j\in \{1,2\} \end{array}}\langle \sigma _{k,j}^{\varepsilon }\cdot \nabla {{A}^{\varepsilon }_{t}}^{\prime }+D\sigma _{k,j}^{\varepsilon }{{A}^{\varepsilon }_{t}}^{\prime },{{A}^{\varepsilon }_{t}}^{\prime } \rangle _{\varepsilon }dW^{k,j}_t \right. \nonumber \\  &\quad \left. +\sum _{\begin{array}{c} k\in \mathbb {Z}^{3,N}_0\\ k_3\ne 0,\quad j\in \{1,2\} \end{array}}\langle \sigma _{k,j}^{\varepsilon }\cdot \nabla \overline{A^{\varepsilon }_{t}}+D\sigma _{k,j}^{\varepsilon }\overline{A^{\varepsilon }_{t}},{{A}^{\varepsilon }_{t}}^{\prime } \rangle _{\varepsilon }dW^{k,j}_t\right) \nonumber \\  &=2\left( \sum _{\begin{array}{c} k\in \mathbb {Z}^{3,N}_0\\ j\in \{1,2\} \end{array}}\langle D\sigma _{k,j}^{\varepsilon }{{A}^{\varepsilon }_{t}}^{\prime },{{A}^{\varepsilon }_{t}}^{\prime } \rangle _{\varepsilon }dW^{k,j}_t \right. \nonumber \\  &\quad \left. +\sum _{\begin{array}{c} k\in \mathbb {Z}^{3,N}_0\\ k_3\ne 0,\quad j\in \{1,2\} \end{array}}\langle \sigma _{k,j}^{\varepsilon }\cdot \nabla \overline{A^{\varepsilon }_{t}}+D\sigma _{k,j}^{\varepsilon }\overline{A^{\varepsilon }_{t}},{{A}^{\varepsilon }_{t}}^{\prime } \rangle _{\varepsilon }dW^{k,j}_t\right) . \end{aligned}$$

Now we can analyse the terms $I^1_t,\ I^2_t,\ I^3_t,\ I^4_t,\ I^5_t,\ I^6_t$. First we observe that due to the fact that derivatives and the operator $(-\Delta )^{-1}$ commute in the periodic framework we easily obtain

3.10 $$\begin{aligned} I^1_t&= \eta \left\| \nabla {{A}^{\varepsilon }_{t}}^{\prime }\right\| _{\varepsilon }^2+\sum _{\begin{array}{c} k\in \mathbb {Z}^{3,N}_0\\ j\in \{1,2\} \end{array}}\left\| \sigma _{k,j}^{\varepsilon }\cdot \nabla {{A}^{\varepsilon }_{t}}^{\prime }\right\| ^2_{\varepsilon }. \end{aligned}$$

Exploiting the fact that $\operatorname {curl}\operatorname {curl}f=-\Delta f$ if *f* is a divergence free vector field we get

$$\begin{aligned} I^2_t&=\sum _{\begin{array}{c} k\in \mathbb {Z}^{3,N}_0\\ k_3=0,\quad j\in \{1,2\} \end{array}}\langle \operatorname {curl}(\sigma _{k,j}^{\varepsilon }\cdot \nabla {{A}^{\varepsilon }_{t}}^{\prime }+D\sigma _{k,j}^{\varepsilon }{{A}^{\varepsilon }_{t}}^{\prime }), (-\Delta )^{-1}\\  &\quad \left( \operatorname {curl}(\sigma _{-k,j}^{\varepsilon }\cdot \nabla {{A}^{\varepsilon }_{t}}^{\prime }+D\sigma _{-k,j}^{\varepsilon }{{A}^{\varepsilon }_{t}}^{\prime })\right) \rangle _{\varepsilon }\\  &=\sum _{\begin{array}{c} k\in \mathbb {Z}^{3,N}_0\\ k_3=0,\quad j\in \{1,2\} \end{array}}\langle \operatorname {curl}\left( P_{\varepsilon }\left[ Q\left[ \sigma _{k,j}^{\varepsilon }\cdot \nabla {{A}^{\varepsilon }_{t}}^{\prime }+D\sigma _{k,j}^{\varepsilon }{{A}^{\varepsilon }_{t}}^{\prime })\right] \right] \right) , \\  &\quad (-\Delta )^{-1}\operatorname {curl}\left( P_{\varepsilon }\left[ Q\left[ \sigma _{k,j}^{\varepsilon }\cdot \nabla {{A}^{\varepsilon }_{t}}^{\prime }+D\sigma _{k,j}^{\varepsilon }{{A}^{\varepsilon }_{t}}^{\prime })\right] \right] \right) \rangle _{\varepsilon }\\  &= \sum _{\begin{array}{c} k\in \mathbb {Z}^{3,N}_0\\ k_3=0,\quad j\in \{1,2\} \end{array}}\left\| P_{\varepsilon }\left[ Q\left[ \sigma _{k,j}^{\varepsilon }\cdot \nabla {{A}^{\varepsilon }_{t}}^{\prime }+D\sigma _{k,j}^{\varepsilon }{{A}^{\varepsilon }_{t}}^{\prime }\right] \right] \right\| ^2_{\varepsilon }\\  &\le \sum _{\begin{array}{c} k\in \mathbb {Z}^{3,N}_0\\ k_3=0,\quad j\in \{1,2\} \end{array}}\left\| \sigma _{k,j}^{\varepsilon }\cdot \nabla {{A}^{\varepsilon }_{t}}^{\prime }+D\sigma _{k,j}^{\varepsilon }{{A}^{\varepsilon }_{t}}^{\prime }\right\| ^2_{\varepsilon }\\  &= \sum _{\begin{array}{c} k\in \mathbb {Z}^{3,N}_0\\ k_3=0,\quad j\in \{1,2\} \end{array}}\left\| \sigma _{k,j}^{\varepsilon }\cdot \nabla {{A}^{\varepsilon }_{t}}^{\prime }\right\| ^2_{\varepsilon }+\sum _{\begin{array}{c} k\in \mathbb {Z}^{3,N}_0\\ k_3=0,\quad j\in \{1,2\} \end{array}}\left\| D\sigma _{k,j}^{\varepsilon }{{A}^{\varepsilon }_{t}}^{\prime }\right\| ^2_{\varepsilon }, \end{aligned}$$

where in the last step we exploited (2.18). By Hölder’s inequality and Poincaré inequality (2.6) the last term in the relation above can be estimated by

$$\begin{aligned}&\sum _{\begin{array}{c} k\in \mathbb {Z}^{3,N}_0\\ k_3=0,\quad j\in \{1,2\} \end{array}}\left\| D\sigma _{k,j}^{\varepsilon }{{A}^{\varepsilon }_{t}}^{\prime }\right\| ^2_{\varepsilon } \le \sum _{\begin{array}{c} k\in \mathbb {Z}^{3,N}_0\\ k_3=0,\quad j\in \{1,2\} \end{array}}(\theta _{k,j}^{\varepsilon })^2|k|^2\left\| {{A}^{\varepsilon }_{t}}^{\prime }\right\| ^2_{\varepsilon }\\  &\quad \le \varepsilon ^2\left\| \partial _3{{A}^{\varepsilon }_{t}}^{\prime }\right\| ^2_{\varepsilon }\left( \sum _{\begin{array}{c} k\in \mathbb {Z}^{3,N}_0\\ k_3=0,\quad j=1 \end{array}}(\theta _{k,j}^{\varepsilon })^2|k|^2+\sum _{\begin{array}{c} k\in \mathbb {Z}^{3,N}_0\\ k_3=0,\quad j=2 \end{array}}(\theta _{k,j}^{\varepsilon })^2|k|^2\right) \\  &\quad \le \varepsilon ^2\left\| \nabla {{A}^{\varepsilon }_{t}}^{\prime }\right\| ^2_{\varepsilon }\left( \sum _{\begin{array}{c} k_H\in \mathbb {Z}^2_0\\ N\le |k_H|\le 2N \end{array}}\frac{1}{C^2_{1,H}}+\sum _{\begin{array}{c} k_H\in \mathbb {Z}^2_0\\ N\le |k_H|\le 2N \end{array}}\frac{1}{C^2_{2,H}|k_H|^{\gamma -2}}\right) \\  &\quad = \left\| \nabla {{A}^{\varepsilon }_{t}}^{\prime }\right\| ^2_{\varepsilon }\left( \frac{\zeta ^N_{H,0}}{C_{1,H}^2}+\frac{\zeta ^N_{H,\gamma -2}}{N^{\gamma -2}C_{2,H}^2}\right) =\left\| \nabla {{A}^{\varepsilon }_{t}}^{\prime }\right\| ^2_{\varepsilon }\left( \frac{\zeta _{H,0}}{C_{1,H}^2}+O(N^{-1})\right) . \end{aligned}$$

Therefore

3.11 $$\begin{aligned} I^2_t \le \sum _{\begin{array}{c} k\in \mathbb {Z}^{3,N}_0\\ k_3=0,\quad j\in \{1,2\} \end{array}}\left\| \sigma _{k,j}^{\varepsilon }\cdot \nabla {{A}^{\varepsilon }_{t}}^{\prime }\right\| ^2_{\varepsilon }+\left\| \nabla {{A}^{\varepsilon }_{t}}^{\prime }\right\| ^2_{\varepsilon }\left( \frac{\zeta _{H,0}}{C_{1,H}^2}+O(N^{-1})\right) . \end{aligned}$$

Analogously we can show that

3.12 $$\begin{aligned} I^3_t&\le \sum _{\begin{array}{c} k\in \mathbb {Z}^{3,N}_0\\ k_3\ne 0,\quad j\in \{1,2\} \end{array}}\left\| \sigma _{k,j}^{\varepsilon }\cdot \nabla \overline{A^{\varepsilon }_{t}}\right\| ^2_{\varepsilon }+\sum _{\begin{array}{c} k\in \mathbb {Z}^{3,N}_0\\ k_3\ne 0,\quad j\in \{1,2\} \end{array}}\left\| D\sigma _{k,j}^{\varepsilon }\overline{A^{\varepsilon }_{t}}\right\| ^2_{\varepsilon }\nonumber \\  &\le \sum _{\begin{array}{c} k\in \mathbb {Z}^{3,N}_0\\ k_3\ne 0,\quad j\in \{1,2\} \end{array}}\left\lvert \theta _{k,j}^{\varepsilon }\right\rvert ^2 \left\| \nabla \overline{A^{\varepsilon }_{t}}\right\| ^2_{\varepsilon }+\sum _{\begin{array}{c} k\in \mathbb {Z}^{3,N}_0\\ k_3\ne 0,\quad j\in \{1,2\} \end{array}}\left\lvert \theta _{k,j}^{\varepsilon }\right\rvert ^2 |k|^2 \left\| \overline{A^{\varepsilon }_{t}}\right\| ^2_{\varepsilon }\nonumber \\  &\le \frac{(\zeta _{H,0} N^2+O(N))\Vert \nabla \overline{A^{\varepsilon }_{t}}\Vert _{\varepsilon }^2}{C_V^2} \sum _{k_3\in N\mathbb {Z}\smallsetminus \{0\}}\frac{1}{|k_3|^{\beta }}+\frac{(\zeta _{H,0}N^2+O(N))\left\| \overline{A^{\varepsilon }_{t}}\right\| _{\varepsilon }^2}{C_V^2} \nonumber \\  &\quad \sum _{k_3\in N\mathbb {Z}\smallsetminus \{0\}}\frac{1}{|k_3|^{\beta -2}}\nonumber \\  &=\left( 2\frac{\zeta _{\beta }\zeta _{H,0} }{N^{\beta -2}C_V^2}+O(N^{1-\beta })\right) \Vert \nabla \overline{A^{\varepsilon }_{t}}\Vert _{\varepsilon }^2+\left( 2\frac{\zeta _{\beta -2}\zeta _{H,0}}{N^{\beta -4}C_V^2}+O(N^{3-\beta })\right) \left\| \overline{A^{\varepsilon }_{t}}\right\| _{\varepsilon }^2. \end{aligned}$$

The analysis of $I^4_t$ is similar recalling that by Lemma 2.3 the operator $N_{\varepsilon }$ and the $\operatorname {curl}$ commute. Therefore we have, arguing as above,

$$\begin{aligned} I^4_t&=\sum _{\begin{array}{c} k\in \mathbb {Z}^{3,N}_0\\ k_3\ne 0,\quad j\in \{1,2\} \end{array}}\left\| P_{\varepsilon }\left[ Q\left[ \left( \sigma _{k,j}^{\varepsilon }\cdot \nabla {{A}^{\varepsilon }_{t}}^{\prime }+D\sigma _{k,j}^{\varepsilon }{{A}^{\varepsilon }_{t}}^{\prime }\right) '\right] \right] \right\| ^2_{\varepsilon }\\  &\le \sum _{\begin{array}{c} k\in \mathbb {Z}^{3,N}_0\\ k_3\ne 0,\quad j\in \{1,2\} \end{array}}\left\| \left( \sigma _{k,j}^{\varepsilon }\cdot \nabla {{A}^{\varepsilon }_{t}}^{\prime }+D\sigma _{k,j}^{\varepsilon }{{A}^{\varepsilon }_{t}}^{\prime }\right) '\right\| ^2_{\varepsilon }\\  &=\sum _{\begin{array}{c} k\in \mathbb {Z}^{3,N}_0\\ k_3\ne 0,\quad j\in \{1,2\} \end{array}}\left\| \sigma _{k,j}^{\varepsilon }\cdot \nabla {{A}^{\varepsilon }_{t}}^{\prime }+D\sigma _{k,j}^{\varepsilon }{{A}^{\varepsilon }_{t}}^{\prime }\right\| ^2_{\varepsilon }\\  &\quad -\sum _{\begin{array}{c} k\in \mathbb {Z}^{3,N}_0\\ k_3\ne 0,\quad j\in \{1,2\} \end{array}}\left\| \overline{\sigma _{k,j}^{\varepsilon }\cdot \nabla {{A}^{\varepsilon }_{t}}^{\prime }+D\sigma _{k,j}^{\varepsilon }{{A}^{\varepsilon }_{t}}^{\prime }}\right\| ^2_{\varepsilon }\\  &\le \sum _{\begin{array}{c} k\in \mathbb {Z}^{3,N}_0\\ k_3\ne 0,\quad j\in \{1,2\} \end{array}}\left\| \sigma _{k,j}^{\varepsilon }\cdot \nabla {{A}^{\varepsilon }_{t}}^{\prime }\right\| ^2_{\varepsilon }+\sum _{\begin{array}{c} k\in \mathbb {Z}^{3,N}_0\\ k_3\ne 0,\quad j\in \{1,2\} \end{array}}\left\| D\sigma _{k,j}^{\varepsilon }{{A}^{\varepsilon }_{t}}^{\prime }\right\| ^2_{\varepsilon }. \end{aligned}$$

In the last step we simply neglected the negative term and exploited (2.18). Arguing as when treating $I^2_t$, we can show by Hölder’s and Poincaré inequality in the thin layer (2.6) that

$$\begin{aligned} \sum _{\begin{array}{c} k\in \mathbb {Z}^{3,N}_0\\ k_3\ne 0,\quad j\in \{1,2\} \end{array}}\left\| D\sigma _{k,j}^{\varepsilon }{{A}^{\varepsilon }_{t}}^{\prime }\right\| ^2_{\varepsilon }&\le \varepsilon ^2\sum _{\begin{array}{c} k\in \mathbb {Z}^{3,N}_0\\ k_3\ne 0,\quad j\in \{1,2\}\\ N\le |k_H|\le 2N \end{array}} \frac{1}{C_V^2 |k|^{\beta -2}}\left\| \nabla {{A}^{\varepsilon }_{t}}^{\prime }\right\| _{\varepsilon }^2\\  &\le \frac{\left( \zeta _{H,0}+O(N^{-1})\right) \left\| \nabla {{A}^{\varepsilon }_{t}}^{\prime }\right\| _{\varepsilon }^2}{C_V^2}\sum _{k_3\in N\mathbb {Z}\smallsetminus \{0\}}\frac{1}{|k_3|^{\beta -2}}\\  &=\left( 2\frac{\zeta _{\beta -2}\zeta _{H,0}}{N^{\beta -2}C_V^2}+O(N^{1-\beta })\right) \left\| \nabla {{A}^{\varepsilon }_{t}}^{\prime }\right\| _{\varepsilon }^2. \end{aligned}$$

In particular, it holds

3.13 $$\begin{aligned} I^4_t \le \sum _{\begin{array}{c} k\in \mathbb {Z}^{3,N}_0\\ k_3\ne 0,\quad j\in \{1,2\} \end{array}}\left\| \sigma _{k,j}^{\varepsilon }\cdot \nabla {{A}^{\varepsilon }_{t}}^{\prime }\right\| ^2_{\varepsilon }+\left( 2\frac{\zeta _{\beta -2}\zeta _{H,0}}{N^{\beta -2}C_V^2}+O(N^{1-\beta })\right) \left\| \nabla {{A}^{\varepsilon }_{t}}^{\prime }\right\| _{\varepsilon }^2. \end{aligned}$$

For what concerns $I^5_t$, applying Cauchy-Schwarz and Young’s inequalities we get

$$\begin{aligned} I^5_t&=\sum _{\begin{array}{c} k\in \mathbb {Z}^{3,N}_0\\ k_3\ne 0,\quad j\in \{1,2\} \end{array}}\langle P_{\varepsilon }\left[ Q\left[ \sigma _{k,j}^{\varepsilon }\cdot \nabla \overline{A^{\varepsilon }_{t}}+D\sigma _{k,j}^{\varepsilon }\overline{A^{\varepsilon }_{t}}\right] \right] ,\\  &\quad P_{\varepsilon }\left[ Q\left[ \left( \sigma _{-k,j}^{\varepsilon }\cdot \nabla {{A}^{\varepsilon }_{t}}^{\prime }+D\sigma _{-k,j}^{\varepsilon }{{A}^{\varepsilon }_{t}}^{\prime }\right) '\right] \right] \rangle _{\varepsilon }\\  &\le \frac{I^3_t+I^4_t}{2}\\  &\le \left( \frac{\zeta _{\beta }\zeta _{H,0} }{N^{\beta -2}C_V^2}+O(N^{1-\beta })\right) \Vert \nabla \overline{A^{\varepsilon }_{t}}\Vert _{\varepsilon }^2+\left( \frac{\zeta _{\beta -2}\zeta _{H,0}}{N^{\beta -4}C_V^2}+O(N^{3-\beta })\right) \left\| \overline{A^{\varepsilon }_{t}}\right\| _{\varepsilon }^2\\  &\quad +\left( \frac{\zeta _{\beta -2}\zeta _{H,0}}{N^{\beta -2}C_V^2}+O(N^{1-\beta })\right) \left\| \nabla {{A}^{\varepsilon }_{t}}^{\prime }\right\| _{\varepsilon }^2+\frac{1}{2}\sum _{\begin{array}{c} k\in \mathbb {Z}^{3,N}_0\\ k_3\ne 0,\quad j\in \{1,2\} \end{array}}\left\| \sigma _{k,j}^{\varepsilon }\cdot \nabla {{A}^{\varepsilon }_{t}}^{\prime }\right\| ^2_{\varepsilon }. \end{aligned}$$

Let us analyse the last term. It holds

$$\begin{aligned} \frac{1}{2}\sum _{\begin{array}{c} k\in \mathbb {Z}^{3,N}_0\\ k_3\ne 0,\quad j\in \{1,2\} \end{array}}\left\| \sigma _{k,j}^{\varepsilon }\cdot \nabla {{A}^{\varepsilon }_{t}}^{\prime }\right\| ^2_{\varepsilon }&\le \frac{\left\| \nabla {{A}^{\varepsilon }_{t}}^{\prime }\right\| _\varepsilon ^2}{2C_V^2}\sum _{\begin{array}{c} k\in \mathbb {Z}^{3,N}_0\\ k_3\ne 0,\quad j\in \{1,2\}\\ N\le |k_H|\le 2N \end{array}}\frac{1}{|k|^{\beta }}\\  &\le \frac{\left( \zeta _{H,0}N^2+O(N)\right) \left\| \nabla {{A}^{\varepsilon }_{t}}^{\prime }\right\| _\varepsilon ^2}{2C_V^2}\sum _{k_3\in N\mathbb {Z}\smallsetminus \{0\}}\frac{1}{|k_3|^{\beta }}\\  &=\left( \frac{\zeta _{\beta }\zeta _{H,0}}{N^{\beta -2}C_V^2}+O(N^{1-\beta })\right) \left\| \nabla {{A}^{\varepsilon }_{t}}^{\prime }\right\| _\varepsilon ^2. \end{aligned}$$

In conclusion it holds

3.14 $$\begin{aligned} I^5_t&\le \left( \frac{\zeta _{\beta }\zeta _{H,0} }{N^{\beta -2}C_V^2}+O(N^{1-\beta })\right) \Vert \nabla \overline{A^{\varepsilon }_{t}}\Vert _{\varepsilon }^2+\left( \frac{\zeta _{\beta -2}\zeta _{H,0}}{N^{\beta -4}C_V^2}+O(N^{3-\beta })\right) \left\| \overline{A^{\varepsilon }_{t}}\right\| _{\varepsilon }^2\nonumber \\  &\quad +\left( 2\frac{\zeta _{\beta }\zeta _{H,0}}{N^{\beta -2}C_V^2}+O(N^{1-\beta })\right) \left\| \nabla {{A}^{\varepsilon }_{t}}^{\prime }\right\| _{\varepsilon }^2. \end{aligned}$$

Lastly we consider $I^6_t$: $\langle \Lambda ^{\varepsilon }_{\rho }{{B}^{\varepsilon }_{t}}^{\prime },(-\Delta )^{-1}{{B}^{\varepsilon }_{t}}^{\prime }\rangle _{\varepsilon }$ can be estimated easily due to Lemma 2.10 and (3.1), obtaining by Cauchy-Schwarz inequality

$$\begin{aligned} \langle \Lambda ^{\varepsilon }_{\rho }{{B}^{\varepsilon }_{t}}^{\prime },(-\Delta )^{-1}{{B}^{\varepsilon }_{t}}^{\prime }\rangle _{\varepsilon }&\le \frac{2\sqrt{2}|\rho |\zeta ^N_{H,\gamma /2} N^{2-\gamma /2} }{C_{1,H}C_{2,H}}\left\| (-\Delta )^{-1/2} {{B}^{\varepsilon }_{t}}^{\prime }\right\| _{\varepsilon }\left\| \nabla {{B}^{\varepsilon }_{t}}^{\prime }\right\| _{H^{-1}(\mathbb {T}^3_{\varepsilon })}\\  &=\frac{2\sqrt{2}|\rho |\zeta ^N_{H,\gamma /2} N^{2-\gamma /2} }{C_{1,H}C_{2,H}}\left\| {{A}^{\varepsilon }_{t}}^{\prime }\right\| _{\varepsilon }\left\| \nabla {{A}^{\varepsilon }_{t}}^{\prime }\right\| _{\varepsilon }. \end{aligned}$$

The other terms can be treated easily by Cauchy-Schwartz inequality and Proposition 3.2. Indeed for each $\xi _1>0$ and $k\in \mathbb {Z}_0^{3,N}$ such that $k_3=0$ we have

$$\begin{aligned}&\langle \mathcal {L}_{\sigma _{k,1}^{\varepsilon }}{{B}^{\varepsilon }_{t}}^{\prime },(-\Delta )^{-1}\mathcal {L}_{\sigma _{-k,2}^{\varepsilon }}{{B}^{\varepsilon }_{t}}^{\prime }\rangle _{\varepsilon }\\  &\quad =\langle \operatorname {curl}\left( \sigma _{k,1}^{\varepsilon }\cdot \nabla {{A}^{\varepsilon }_{t}}^{\prime }+D\sigma _{k,1}^{\varepsilon }{{A}^{\varepsilon }_{t}}^{\prime }\right) ,(-\Delta )^{-1}\operatorname {curl}\left( \sigma _{-k,2}^{\varepsilon }\cdot \nabla {{A}^{\varepsilon }_{t}}^{\prime }+D\sigma _{-k,2}^{\varepsilon }{{A}^{\varepsilon }_{t}}^{\prime }\right) \rangle _{\varepsilon }\\  &\quad =\langle \operatorname {curl}\left( P_\varepsilon [Q[\sigma _{k,1}^{\varepsilon }\cdot \nabla {{A}^{\varepsilon }_{t}}^{\prime }+D\sigma _{k,1}^{\varepsilon }{{A}^{\varepsilon }_{t}}^{\prime }]]\right) ,(-\Delta )^{-1}\\  &\quad \quad \operatorname {curl}\left( P_\varepsilon [Q[\sigma _{-k,2}^{\varepsilon }\cdot \nabla {{A}^{\varepsilon }_{t}}^{\prime }+D\sigma _{-k,2}^{\varepsilon }{{A}^{\varepsilon }_{t}}^{\prime }]]\right) \rangle _{\varepsilon }\\  &\quad =\langle \left( P_\varepsilon [Q[\sigma _{k,1}^{\varepsilon }\cdot \nabla {{A}^{\varepsilon }_{t}}^{\prime }+D\sigma _{k,1}^{\varepsilon }{{A}^{\varepsilon }_{t}}^{\prime }]]\right) ,\left( P_\varepsilon [Q[\sigma _{-k,2}^{\varepsilon }\cdot \nabla {{A}^{\varepsilon }_{t}}^{\prime }+D\sigma _{-k,2}^{\varepsilon }{{A}^{\varepsilon }_{t}}^{\prime }]]\right) \rangle _{\varepsilon }\\  &\quad \le \frac{\left\| \sigma _{k,1}^{\varepsilon }\cdot \nabla {{A}^{\varepsilon }_{t}}^{\prime }+D\sigma _{k,1}^{\varepsilon }{{A}^{\varepsilon }_{t}}^{\prime }\right\| _{\varepsilon }^2}{2\xi _1}+ \frac{\xi _1 \left\| \sigma _{k,2}^{\varepsilon }\cdot \nabla {{A}^{\varepsilon }_{t}}^{\prime }+D\sigma _{k,2}^{\varepsilon }{{A}^{\varepsilon }_{t}}^{\prime }\right\| _{\varepsilon }^2}{2}. \end{aligned}$$

So far, arguing when treating $I^2_t$, we proved that for each $\xi _1>0$

3.15 $$\begin{aligned} I^6_t&\le \frac{2\sqrt{2}|\rho |\zeta ^N_{H,\gamma /2} }{N^{\gamma /2-2}C_{1,H}C_{2,H}}\left\| {{A}^{\varepsilon }_{t}}^{\prime }\right\| _{\varepsilon }\left\| \nabla {{A}^{\varepsilon }_{t}}^{\prime }\right\| _{\varepsilon }+\frac{|\rho |}{\xi _1} \sum _{\begin{array}{c} k\in \mathbb {Z}^{3,N}_0\\ k_3=0,\quad j=1 \end{array}}\left\| \sigma _{k,j}^{\varepsilon }\cdot \nabla {{A}^{\varepsilon }_{t}}^{\prime }\right\| ^2_{\varepsilon }\nonumber \\  &\quad + |\rho |\xi _1 \sum _{\begin{array}{c} k\in \mathbb {Z}^{3,N}_0\\ k_3=0,\quad j=2 \end{array}}\left\| \sigma _{k,j}^{\varepsilon }\cdot \nabla {{A}^{\varepsilon }_{t}}^{\prime }\right\| ^2_{\varepsilon }+\left\| \nabla {{A}^{\varepsilon }_{t}}^{\prime }\right\| _{\varepsilon }^2\left( \frac{|\rho |\zeta _{H,0}}{\xi _1 C_{1,H}^2}+O(N^{-1})\right) . \end{aligned}$$

Let us analyse $$\sum _{\begin{array}{c} k\in \mathbb {Z}^{3,N}_0\\ k_3=0,\quad j=1 \end{array}}\left\| \sigma _{k,j}^{\varepsilon }\cdot \nabla {{A}^{\varepsilon }_{t}}^{\prime }\right\| ^2_{\varepsilon }$$ and $$\sum _{\begin{array}{c} k\in \mathbb {Z}^{3,N}_0\\ k_3=0,\quad j=2 \end{array}}\left\| \sigma _{k,j}^{\varepsilon }\cdot \nabla {{A}^{\varepsilon }_{t}}^{\prime }\right\| ^2_{\varepsilon }$$. We have

$$\begin{aligned} \sum _{\begin{array}{c} k\in \mathbb {Z}^{3,N}_0\\ k_3=0,\quad j=1 \end{array}}\left\| \sigma _{k,j}^{\varepsilon }\cdot \nabla {{A}^{\varepsilon }_{t}}^{\prime }\right\| ^2_{\varepsilon }&\le \frac{\left\| \nabla {{A}^{\varepsilon }_{t}}^{\prime }\right\| _{\varepsilon }^2}{C_{1,H}^2}\sum _{\begin{array}{c} k_H\in \mathbb {Z}^2_0\\ N\le |k_H|\le 2N \end{array}}\frac{1}{|k_H|^2}\\  &=\left( \frac{\zeta _{H,2}}{C_{1,H}^2}+O(N^{-1})\right) \left\| \nabla {{A}^{\varepsilon }_{t}}^{\prime }\right\| _{\varepsilon }^2 \end{aligned}$$

and analogously

$$\begin{aligned} \sum _{\begin{array}{c} k\in \mathbb {Z}^{3,N}_0\\ k_3=0,\quad j=2 \end{array}}\left\| \sigma _{k,j}^{\varepsilon }\cdot \nabla {{A}^{\varepsilon }_{t}}^{\prime }\right\| ^2_{\varepsilon }&\le \left( \frac{\zeta _{H,\gamma }}{N^{\gamma -2}C_{1,H}^2}+O(N^{1-\gamma })\right) \left\| \nabla {{A}^{\varepsilon }_{t}}^{\prime }\right\| _{\varepsilon }^2. \end{aligned}$$

In conclusion, applying Poincaré inequality (2.6) to (3.15), it holds

3.16 $$\begin{aligned} I^6_t&\le \frac{|\rho |}{\xi _1}\left( \frac{\zeta _{H,0}+\zeta _{H,2}}{C_{1,H}^2}+O(N^{-1})\right) \left\| \nabla {{A}^{\varepsilon }_{t}}^{\prime }\right\| _{\varepsilon }^2. \end{aligned}$$

Combining (3.9), (3.10), (3.11), (3.12), (3.13), (3.14), (3.16) we get relation (3.6).

Now we move on to the analysis of $\left\| {{B}^{3,\varepsilon }_{t}}^{\prime }\right\| ^2_{\varepsilon }$ which is similar, actually easier, than the previous one. Indeed, by Itô formula it holds:

$$\begin{aligned} d\left\| {{B}^{3,\varepsilon }_{t}}^{\prime }\right\| ^2_{\varepsilon }=-d\tilde{W}'_t+2(-J^1_t+J^2_t+J^3_t+J^4_t+J^5_t+J^6_t)dt, \end{aligned}$$

where we denote by

$$\begin{aligned} d\tilde{W}'_t&=2\sum _{\begin{array}{c} k\in \mathbb {Z}^{3,N}_0\\ k_3=0,\quad j\in \{1,2\} \end{array}}\langle \sigma _{k,j}^{\varepsilon }\cdot \nabla {{B}^{3,\varepsilon }_{t}}^{\prime }-{{B}^{\varepsilon }_{t}}^{\prime }\cdot \nabla \sigma _{k,j}^{3,\varepsilon },{{B}^{3,\varepsilon }_{t}}^{\prime } \rangle _{\varepsilon }dW^{k,j}_t \\&\quad +2\sum _{\begin{array}{c} k\in \mathbb {Z}^{3,N}_0\\ k_3\ne 0,\quad j\in \{1,2\} \end{array}}\langle \sigma _{k,j}^{\varepsilon }\cdot \nabla \overline{B^{3,\varepsilon }_{t}}-\overline{B^{\varepsilon }_{t}}\cdot \nabla \sigma _{k,j}^{3,\varepsilon },{{B}^{3,\varepsilon }_{t}}^{\prime } \rangle _{\varepsilon }dW^{k,j}_t \\&\quad +2\sum _{\begin{array}{c} k\in \mathbb {Z}^{3,N}_0\\ k_3\ne 0,\quad j\in \{1,2\} \end{array}}\langle \left( \sigma _{k,j}^{\varepsilon }\cdot \nabla {{B}^{3,\varepsilon }_{t}}^{\prime }-{{B}^{\varepsilon }_{t}}^{\prime }\cdot \nabla \sigma _{k,j}^{3,\varepsilon }\right) ',{{B}^{3,\varepsilon }_{t}}^{\prime } \rangle _{\varepsilon }dW^{k,j}_t,\\ J^1_t&=\eta \left\| \nabla {{B}^{3,\varepsilon }_{t}}^{\prime }\right\| _{\varepsilon }^2+\sum _{\begin{array}{c} k\in \mathbb {Z}^{3,N}_0\\ j\in \{1,2\} \end{array}}\left\| \sigma _{k,j}^{\varepsilon }\cdot \nabla {{B}^{3,\varepsilon }_{t}}^{\prime }\right\| _{\varepsilon }^2,\\ J^2_t&=\sum _{\begin{array}{c} k\in \mathbb {Z}^{3,N}_0\\ k_3=0,\quad j\in \{1,2\} \end{array}}\langle \sigma _{k,j}^{\varepsilon }\cdot \nabla {{B}^{3,\varepsilon }_{t}}^{\prime }-{{B}^{\varepsilon }_{t}}^{\prime }\cdot \nabla \sigma _{k,j}^{3,\varepsilon }, \sigma _{-k,j}^{\varepsilon }\cdot \nabla {{B}^{3,\varepsilon }_{t}}^{\prime }-{{B}^{\varepsilon }_{t}}^{\prime }\cdot \nabla \sigma _{-k,j}^{3,\varepsilon }\rangle _{\varepsilon },\\J^3_t&= \sum _{\begin{array}{c} k\in \mathbb {Z}^{3,N}_0\\ k_3\ne 0,\quad j\in \{1,2\} \end{array}}\langle \sigma _{k,j}^{\varepsilon }\cdot \nabla \overline{B^{3,\varepsilon }_{t}}-\overline{B^{\varepsilon }_{t}}\cdot \nabla \sigma _{k,j}^{3,\varepsilon }, \sigma _{-k,j}^{\varepsilon }\cdot \nabla \overline{B^{3,\varepsilon }_{t}}-\overline{B^{\varepsilon }_{t}}\cdot \nabla \sigma _{-k,j}^{3,\varepsilon }\rangle _{\varepsilon },\\ J^4_t&=\sum _{\begin{array}{c} k\in \mathbb {Z}^{3,N}_0\\ k_3\ne 0,\quad j\in \{1,2\} \end{array}}\langle \left( \sigma _{k,j}^{\varepsilon }\cdot \nabla {{B}^{3,\varepsilon }_{t}}^{\prime }-{{B}^{\varepsilon }_{t}}^{\prime }\cdot \nabla \sigma _{k,j}^{3,\varepsilon }\right) ', \\  &\quad \left( \sigma _{-k,j}^{\varepsilon }\cdot \nabla {{B}^{3,\varepsilon }_{t}}^{\prime }-{{B}^{\varepsilon }_{t}}^{\prime }\cdot \nabla \sigma _{-k,j}^{3,\varepsilon }\right) '\rangle _{\varepsilon }, \\ J^5_t&=\sum _{\begin{array}{c} k\in \mathbb {Z}^{3,N}_0\\ k_3\ne 0,\quad j\in \{1,2\} \end{array}}\langle \sigma _{k,j}^{\varepsilon }\cdot \nabla \overline{B^{3,\varepsilon }_{t}}-\overline{B^{\varepsilon }_{t}}\cdot \nabla \sigma _{k,j}^{3,\varepsilon },\left( \sigma _{-k,j}^{\varepsilon }\cdot \nabla {{B}^{3,\varepsilon }_{t}}^{\prime }-{{B}^{\varepsilon }_{t}}^{\prime }\cdot \nabla \sigma _{-k,j}^{3,\varepsilon }\right) '\rangle _{\varepsilon },\\ J^6_t&=-\langle \operatorname {div}_H\left( \mathcal {R}_{\varepsilon ,\gamma } {{B}^{H,\varepsilon }_{t}}^{\prime }\right) ,{{B}^{3,\varepsilon }_{t}}^{\prime }\rangle _{\varepsilon }\\  &\quad +\rho \sum _{\begin{array}{c} k\in \mathbb {Z}^{3,N}_0\\ k_3=0 \end{array}}\langle \sigma _{k,1}^{\varepsilon }\cdot \nabla {{B}^{3,\varepsilon }_{t}}^{\prime },\sigma _{-k,2}^{\varepsilon }\cdot \nabla {{B}^{3,\varepsilon }_{t}}^{\prime }\\  &\quad -{{B}^{\varepsilon }_{t}}^{\prime }\cdot \nabla \sigma _{-k,2}^{3,\varepsilon }\rangle _{\varepsilon }+\langle \sigma _{k,2}^{\varepsilon }\cdot \nabla {{B}^{3,\varepsilon }_{t}}^{\prime }-{{B}^{\varepsilon }_{t}}^{\prime }\cdot \nabla \sigma _{k,2}^{3,\varepsilon } ,\sigma _{-k,1}^{\varepsilon }\cdot \nabla {{B}^{3,\varepsilon }_{t}}^{\prime }\rangle _{\varepsilon }. \end{aligned}$$

Since the $\sigma _{k,j}^{\varepsilon }$ are divergence free, the martingale term is equal to

3.17 $$\begin{aligned} d\tilde{W}'_t&=-2\sum _{\begin{array}{c} k\in \mathbb {Z}^{3,N}_0\\ j\in \{1,2\} \end{array}} \langle {{B}^{\varepsilon }_{t}}^{\prime }\cdot \nabla \sigma _{k,j}^{3,\varepsilon },{{B}^{3,\varepsilon }_{t}}^{\prime } \rangle _{\varepsilon }dW^{k,j}_t \nonumber \\  &\quad +2\sum _{\begin{array}{c} k\in \mathbb {Z}^{3,N}_0\\ k_3\ne 0,\quad j\in \{1,2\} \end{array}}\langle \sigma _{k,j}^{\varepsilon }\cdot \nabla \overline{B^{3,\varepsilon }_{t}}-\overline{B^{\varepsilon }_{t}}\cdot \nabla \sigma _{k,j}^{3,\varepsilon },{{B}^{3,\varepsilon }_{t}}^{\prime } \rangle _{\varepsilon }dW^{k,j}_t. \end{aligned}$$

The analysis of $J^2_t,\ J^3_t,\ J^4_t,\ J^5_t$ is similar to the corresponding terms when estimating $\left\| {{A}^{\varepsilon }_{t}}^{\prime }\right\| _{\varepsilon }^2$ and is based on the fact that the mixed products in each inner product are equal to 0 thanks to (2.18). In order to treat $J_t^2$ let us preliminarily observe that if $k_3=0$, then $\sigma _{k,j}^{3,\varepsilon }\ne 0$ if and only if j=2. Therefore we get

3.18 $$\begin{aligned} J_t^2&=\sum _{\begin{array}{c} k\in \mathbb {Z}^{3,N}_0\\ k_3=0,\quad j\in \{1,2\} \end{array}}\left\| \sigma _{k,j}^{\varepsilon }\cdot \nabla {{B}^{3,\varepsilon }_{t}}^{\prime }-{{B}^{\varepsilon }_{t}}^{\prime }\cdot \nabla \sigma _{k,j}^{3,\varepsilon }\right\| ^2_{\varepsilon }\nonumber \\  &= \sum _{\begin{array}{c} k\in \mathbb {Z}^{3,N}_0\\ k_3=0,\quad j\in \{1,2\} \end{array}}\left\| \sigma _{k,j}^{\varepsilon }\cdot \nabla {{B}^{3,\varepsilon }_{t}}^{\prime }\right\| ^2_{\varepsilon }+\sum _{\begin{array}{c} k\in \mathbb {Z}^{3,N}_0\\ k_3=0,\quad j=2 \end{array}}\left\| {{B}^{\varepsilon }_{t}}^{\prime }\cdot \nabla \sigma _{k,j}^{3,\varepsilon }\right\| ^2_{\varepsilon }\nonumber \\  &\le \sum _{\begin{array}{c} k\in \mathbb {Z}^{3,N}_0\\ k_3=0,\quad j\in \{1,2\} \end{array}}\left\| \sigma _{k,j}^{\varepsilon }\cdot \nabla {{B}^{3,\varepsilon }_{t}}^{\prime }\right\| ^2_{\varepsilon }+\sum _{\begin{array}{c} k_H\in \mathbb {Z}^2_0\\ N\le |k_H|\le 2N \end{array}}\frac{1}{C_{2,H}^2|k_H|^{\gamma -2}}\left\| {{B}^{\varepsilon }_{t}}^{\prime }\right\| ^2_{\varepsilon }\nonumber \\&\le \sum _{\begin{array}{c} k\in \mathbb {Z}^{3,N}_0\\ k_3=0,\quad j\in \{1,2\} \end{array}}\left\| \sigma _{k,j}^{\varepsilon }\cdot \nabla {{B}^{3,\varepsilon }_{t}}^{\prime }\right\| ^2_{\varepsilon }+\left( \frac{\zeta _{H,\gamma -2}}{N^{\gamma -4}C_{2,H}^2}+O(N^{3-\gamma })\right) \left\| {{B}^{\varepsilon }_{t}}^{\prime }\right\| ^2_{\varepsilon }\nonumber \\  &=\sum _{\begin{array}{c} k\in \mathbb {Z}^{3,N}_0\\ k_3=0,\quad j\in \{1,2\} \end{array}}\left\| \sigma _{k,j}^{\varepsilon }\cdot \nabla {{B}^{3,\varepsilon }_{t}}^{\prime }\right\| ^2_{\varepsilon }+\left( \frac{\zeta _{H,\gamma -2}}{N^{\gamma -4}C_{2,H}^2}+O(N^{3-\gamma })\right) \left\| \nabla {{A}^{\varepsilon }_{t}}^{\prime }\right\| ^2_{\varepsilon }, \end{aligned}$$

exploiting relation (3.1) in the last equality. The analysis of $J_t^3$ is analogous to that of $J_t^2$ and lead us to

3.19 $$\begin{aligned} J^3_t&\le \sum _{\begin{array}{c} k\in \mathbb {Z}^{3,N}_0\\ k_3\ne 0,\quad j\in \{1,2\} \end{array}}\left\| \sigma _{k,j}^{\varepsilon }\cdot \nabla \overline{B^{3,\varepsilon }_{t}}\right\| ^2_{\varepsilon }+\left( 2\frac{\zeta _{\beta -2}\zeta _{H,0}}{N^{\beta -4}C_{V}^2}+O(N^{3-\beta })\right) \left\| \overline{B^{\varepsilon }_{t}}\right\| ^2_{\varepsilon }. \end{aligned}$$

For what concerns $J^4_t$, exploiting the fact that $N_{\varepsilon }$ is a projection on $\dot{L}^2(\mathbb {T}^3_{\varepsilon })$ we get easily

$$\begin{aligned} J_t^4&=\sum _{\begin{array}{c} k\in \mathbb {Z}^{3,N}_0\\ k_3\ne 0,\quad j\in \{1,2\} \end{array}}\left\| \sigma _{k,j}^{\varepsilon }\cdot \nabla {{B}^{3,\varepsilon }_{t}}^{\prime }-{{B}^{\varepsilon }_{t}}^{\prime }\cdot \nabla \sigma _{k,j}^{3,\varepsilon }\right\| _{\varepsilon }^2\\  &\quad -\sum _{\begin{array}{c} k\in \mathbb {Z}^{3,N}_0\\ k_3\ne 0,\quad j\in \{1,2\} \end{array}}\left\| \overline{\sigma _{k,j}^{\varepsilon }\cdot \nabla {{B}^{3,\varepsilon }_{t}}^{\prime }-{{B}^{\varepsilon }_{t}}^{\prime }\cdot \nabla \sigma _{k,j}^{3,\varepsilon }}\right\| _{\varepsilon }^2 \\  &=\sum _{\begin{array}{c} k\in \mathbb {Z}^{3,N}_0\\ k_3\ne 0,\quad j\in \{1,2\} \end{array}}\left( \left\| \sigma _{k,j}^{\varepsilon }\cdot \nabla {{B}^{3,\varepsilon }_{t}}^{\prime }\right\| _{\varepsilon }^2+\left\| {{B}^{\varepsilon }_{t}}^{\prime }\cdot \nabla \sigma _{k,j}^{3,\varepsilon }\right\| _{\varepsilon }^2-\left\| \overline{\sigma _{k,j}^{\varepsilon }\cdot \nabla {{B}^{3,\varepsilon }_{t}}^{\prime } -{{B}^{\varepsilon }_{t}}^{\prime }\cdot \nabla \sigma _{k,j}^{3,\varepsilon }}\right\| _{\varepsilon }^2\right) . \end{aligned}$$

Let us analyse the second term in the last sum. We have by Hölder’s inequality and exploiting our definition of the $\sigma _{k,j}^{\varepsilon }$

$$\begin{aligned} \sum _{\begin{array}{c} k\in \mathbb {Z}^{3,N}_0\\ k_3\ne 0,\quad j\in \{1,2\} \end{array}}\left\| {{B}^{\varepsilon }_{t}}^{\prime }\cdot \nabla \sigma _{k,j}^{3,\varepsilon }\right\| _{\varepsilon }^2&\le \sum _{\begin{array}{c} k\in \mathbb {Z}^{3,N}_0\\ k_3\ne 0,\quad j\in \{1,2\}\\ N\le |k_H|\le 2N \end{array}} \frac{1}{C_V^2 |k|^{\beta -2}}\left\| {{B}^{\varepsilon }_{t}}^{\prime }\right\| _{\varepsilon }^2\\  &\le \left( 2\frac{\zeta _{\beta -2}\zeta _{H,0}}{N^{\beta -4}C_V^2}+O(N^{3-\beta })\right) \Vert \nabla {{A}^{\varepsilon }_{t}}^{\prime }\Vert _{\varepsilon }^2. \end{aligned}$$

In conclusion we get

3.20 $$\begin{aligned} J_t^4&\le \sum _{\begin{array}{c} k\in \mathbb {Z}^{3,N}_0\\ k_3\ne 0,\quad j\in \{1,2\} \end{array}}\left( \left\| \sigma _{k,j}^{\varepsilon }\cdot \nabla {{B}^{3,\varepsilon }_{t}}^{\prime }\right\| _{\varepsilon }^2-\left\| \overline{\sigma _{k,j}^{\varepsilon }\cdot \nabla {{B}^{3,\varepsilon }_{t}}^{\prime }-{{B}^{\varepsilon }_{t}}^{\prime }\cdot \nabla \sigma _{k,j}^{3,\varepsilon }}\right\| _{\varepsilon }^2\right) \nonumber \\  &\quad +\left( 2\frac{\zeta _{\beta -2}\zeta _{H,0}}{N^{\beta -4}C_V^2}+O(N^{3-\beta })\right) \Vert \nabla {{A}^{\varepsilon }_{t}}^{\prime }\Vert _{\varepsilon }^2. \end{aligned}$$

Exploiting the fact that for each $f,g\in L^2(\mathbb {T}^3_{\varepsilon })$ we have $\langle \overline{f},g'\rangle _{\varepsilon }=0$ we get $J^5_t=0$. Indeed, thanks to the mirror symmetry property of the covariance function (2.18) and the definition of the $\sigma _{k,j}^{\varepsilon }$ we have

$$\begin{aligned} J^5_t&=\sum _{\begin{array}{c} k\in \mathbb {Z}^{3,N}_0\\ k_3\ne 0,\quad j\in \{1,2\} \end{array}}\langle \sigma _{k,j}^{\varepsilon }\cdot \nabla \overline{B^{3,\varepsilon }_{t}}-\overline{B^{\varepsilon }_{t}}\cdot \nabla \sigma _{k,j}^{3,\varepsilon },\sigma _{-k,j}^{\varepsilon }\cdot \nabla {{B}^{3,\varepsilon }_{t}}^{\prime }-{{B}^{\varepsilon }_{t}}^{\prime }\cdot \nabla \sigma _{-k,j}^{3,\varepsilon }\rangle _{\varepsilon }\\  &=\sum _{\begin{array}{c} k\in \mathbb {Z}^{3,N}_0\\ k_3\ne 0,\quad j\in \{1,2\} \end{array}}\langle \sigma _{k,j}^{\varepsilon }\cdot \nabla \overline{B^{3,\varepsilon }_{t}},\sigma _{-k,j}^{\varepsilon }\cdot \nabla {{B}^{3,\varepsilon }_{t}}^{\prime }\rangle _{\varepsilon }\\  &\quad +\sum _{\begin{array}{c} k\in \mathbb {Z}^{3,N}_0\\ k_3\ne 0,\quad j\in \{1,2\} \end{array}}\langle \overline{B^{\varepsilon }_{t}}\cdot \nabla \sigma _{k,j}^{3,\varepsilon },{{B}^{\varepsilon }_{t}}^{\prime }\cdot \nabla \sigma _{-k,j}^{3,\varepsilon }\rangle _{\varepsilon }\\  &=\sum _{\begin{array}{c} k\in \mathbb {Z}^{3,N}_0\\ k_3\ne 0,\quad j\in \{1,2\} \end{array}}\left\lvert \theta _{k,j}^{\varepsilon }\right\rvert ^2\langle a_{k,j}\cdot \nabla \overline{B^{3,\varepsilon }_{t}},a_{-k,j}\cdot \nabla {{B}^{3,\varepsilon }_{t}}^{\prime }\rangle _{\varepsilon }\\  &\quad +\sum _{\begin{array}{c} k\in \mathbb {Z}^{3,N}_0\\ k_3\ne 0,\quad j\in \{1,2\} \end{array}}\left\lvert \theta _{k,j}^{\varepsilon }\right\rvert ^2 \left( a_{k,j}^3\right) ^2\langle \overline{B^{\varepsilon }_{t}}\cdot k, {{B}^{\varepsilon }_{t}}^{\prime }\cdot k\rangle \\  &=0. \end{aligned}$$

Lastly let us treat $J^6_t$: the term $-\langle \operatorname {div}_H\left( \mathcal {R}_{\varepsilon ,\gamma } {{B}^{H,\varepsilon }_{t}}^{\prime }\right) ,{{B}^{3,\varepsilon }_{t}}^{\prime }\rangle _{\varepsilon }$ can be estimated easily due to Lemma 2.10 and (3.1) getting by Cauchy-Schwarz inequality

$$\begin{aligned} -\langle \operatorname {div}_H\left( \mathcal {R}_{\varepsilon ,\gamma } {{B}^{H,\varepsilon }_{t}}^{\prime }\right) ,{{B}^{3,\varepsilon }_{t}}^{\prime }\rangle _{\varepsilon }&= \langle \mathcal {R}_{\varepsilon ,\gamma } {{B}^{H,\varepsilon }_{t}}^{\prime },\nabla _H{{B}^{3,\varepsilon }_{t}}^{\prime }\rangle _{\varepsilon }\\  &\le \frac{2\sqrt{2}|\rho |\zeta ^N_{H,\gamma /2} }{N^{\gamma /2-2}C_{1,H}C_{2,H}}\left\| \nabla {{B}^{3,\varepsilon }_{t}}^{\prime }\right\| _\varepsilon \left\| \nabla {{A}^{\varepsilon }_{t}}^{\prime }\right\| _\varepsilon . \end{aligned}$$

The other terms can be treated easily by Cauchy-Schwartz inequality obtaining for each $\xi _2>0$ and $k\in \mathbb {Z}_0^{3,N}$ such that $k_3=0$

$$\begin{aligned}&\langle \sigma _{k,1}^{\varepsilon }\cdot \nabla {{B}^{3,\varepsilon }_{t}}^{\prime },\sigma _{-k,2}^{\varepsilon }\cdot \nabla {{B}^{3,\varepsilon }_{t}}^{\prime }-{{B}^{\varepsilon }_{t}}^{\prime }\cdot \nabla \sigma _{-k,2}^{3,\varepsilon }\rangle _{\varepsilon }\\  &\qquad \le \frac{\left\| \sigma _{k,1}^{\varepsilon }\cdot \nabla {{B}^{3,\varepsilon }_{t}}^{\prime }\right\| _{\varepsilon }^2}{2\xi _2}+\frac{\xi _2 \left\| \sigma _{k,2}^{\varepsilon }\cdot \nabla {{B}^{3,\varepsilon }_{t}}^{\prime }-{{B}^{\varepsilon }_{t}}^{\prime }\cdot \nabla \sigma _{k,2}^{3,\varepsilon }\right\| _{\varepsilon }^2}{2}. \end{aligned}$$

So far, arguing when treating $J^2_t$, we proved that for each $\xi _2>0$

3.21 $$\begin{aligned} J^6_t&\le \frac{2\sqrt{2}|\rho |\zeta ^N_{H,\gamma /2} }{N^{\gamma /2-2} C_{1,H}C_{2,H}}\left\| \nabla {{B}^{3,\varepsilon }_{t}}^{\prime }\right\| _{\varepsilon }\left\| \nabla {{A}^{\varepsilon }_{t}}^{\prime }\right\| _{\varepsilon }+\frac{|\rho |}{\xi _2} \sum _{\begin{array}{c} k\in \mathbb {Z}^{3,N}_0\\ k_3=0,\quad j=1 \end{array}}\left\| \sigma _{k,j}^{\varepsilon }\cdot \nabla {{B}^{3,\varepsilon }_{t}}^{\prime }\right\| ^2_{\varepsilon }\nonumber \\  &\quad +|\rho |\xi _2 \sum _{\begin{array}{c} k\in \mathbb {Z}^{3,N}_0\\ k_3=0,\quad j=2 \end{array}}\left\| \sigma _{k,j}^{\varepsilon }\cdot \nabla {{B}^{3,\varepsilon }_{t}}^{\prime }\right\| ^2_{\varepsilon }+\left( \frac{|\rho |\xi _2\zeta _{H,\gamma -2}}{N^{\gamma -4}C_{2,H}^2}+O(N^{3-\gamma })\right) \left\| \nabla {{A}^{\varepsilon }_{t}}^{\prime }\right\| ^2_{\varepsilon }. \end{aligned}$$

Let us analyse $$\sum _{\begin{array}{c} k\in \mathbb {Z}^{3,N}_0\\ k_3=0,\quad j=1 \end{array}}\left\| \sigma _{k,j}^{\varepsilon }\cdot \nabla {{B}^{3,\varepsilon }_{t}}^{\prime }\right\| ^2_{\varepsilon }$$ and $$\sum _{\begin{array}{c} k\in \mathbb {Z}^{3,N}_0\\ k_3=0,\quad j=2 \end{array}}\left\| \sigma _{k,j}^{\varepsilon }\cdot \nabla {{B}^{3,\varepsilon }_{t}}^{\prime }\right\| ^2_{\varepsilon }$$. Arguing when treating $I^6_t$ we have

$$\begin{aligned} \sum _{\begin{array}{c} k\in \mathbb {Z}^{3,N}_0\\ k_3=0,\quad j=1 \end{array}}\left\| \sigma _{k,j}^{\varepsilon }\cdot \nabla {{B}^{3,\varepsilon }_{t}}^{\prime }\right\| ^2_{\varepsilon }&\le \left( \frac{\zeta _{H,2}}{C_{1,H}^2}+O(N^{-1})\right) \left\| \nabla {{B}^{3,\varepsilon }_{t}}^{\prime }\right\| _{\varepsilon }^2,\\ \sum _{\begin{array}{c} k\in \mathbb {Z}^{3,N}_0\\ k_3=0,\quad j=2 \end{array}}\left\| \sigma _{k,j}^{\varepsilon }\cdot \nabla {{B}^{3,\varepsilon }_{t}}^{\prime }\right\| ^2_{\varepsilon }&\le \left( \frac{\zeta _{H,\gamma }}{N^{\gamma -2}C_{1,H}^2}+O(N^{1-\gamma })\right) \left\| \nabla {{B}^{3,\varepsilon }_{t}}^{\prime }\right\| _{\varepsilon }^2. \end{aligned}$$

In conclusion, applying Young’s inequality to the first term in (3.21), it holds

3.22 $$\begin{aligned} J^6_t&\le \frac{|\rho |}{\xi _2}\left( \frac{\zeta _{H,2}}{ C_{1,H}^2}+\sqrt{2}\frac{\zeta _{H,\gamma /2}}{C_{1,H}C_{2,H}}+O(N^{-1})\right) \left\| \nabla {{B}^{3,\varepsilon }_{t}}^{\prime }\right\| _{\varepsilon }^2\nonumber \\  &\quad +|\rho |\xi _2\left( \frac{\zeta _{H,\gamma -2}}{N^{\gamma -4}C_{2,H}^2}+\frac{\zeta _{H,\gamma /2}}{N^{\gamma -4}C_{1,H}C_{2,H}}+O(N^{3-\gamma })\right) \left\| \nabla {{A}^{\varepsilon }_{t}}^{\prime }\right\| ^2_{\varepsilon }. \end{aligned}$$

Combining (3.17), (3.18), (3.19), (3.20), (3.22), relation (3.7) follows. □

### Lemma 3.4

Assuming Hypothesis 2.6, for each $\xi _3>0$ we have

3.23 $$\begin{aligned}&d\left\| \overline{A^{3,\varepsilon }_{t}}\right\| _{\varepsilon }^2+2\eta \left\| \nabla \overline{A^{3,\varepsilon }_{t}}\right\| _{\varepsilon }^2 dt\le -2\sum _{\begin{array}{c} k\in \mathbb {Z}^{3,N}_0\\ k_3\ne 0,\quad j\in \{1,2\} \end{array}}\langle \sigma _{k,j}^{\varepsilon }\cdot \nabla {{A}^{3,\varepsilon }_{t}}^{\prime }+\partial _3\sigma _{k,j}^{\varepsilon }\cdot {{A}^{\varepsilon }_{t}}^{\prime },\overline{A^{3,\varepsilon }_{t}}\rangle _{\varepsilon }dW^{k,j}_t\nonumber \\  &\qquad +\left( 4\frac{\zeta _{\beta }\zeta _{H,0}}{N^{\beta -2}C_V^2}+O(N^{1-\beta })\right) \left\| \nabla {{A}^{3,\varepsilon }_{t}}^{\prime }\right\| _{\varepsilon }^2dt+\left( 4\frac{\zeta _{\beta -2}\zeta _{H,0}}{N^{\beta -4}C_V^2}+O(N^{3-\beta })\right) \left\| {{A}^{\varepsilon }_{t}}^{\prime }\right\| _{\varepsilon }^2dt, \end{aligned}$$

3.24 $$\begin{aligned}&d\left\| \overline{B^{3,\varepsilon }_{t}}\right\| _{\varepsilon }^2+2\eta \left\| \nabla \overline{B^{3,\varepsilon }_{t}}\right\| _{\varepsilon }^2+2\sum _{\begin{array}{c} k\in \mathbb {Z}^{3,N}_0\\ k_3\ne 0,\quad j\in \{1,2\} \end{array}}\left\| \sigma _{k,j}^{\varepsilon }\cdot \nabla \overline{B^{3,\varepsilon }_{t}} \right\| _{\varepsilon }^2 dt\nonumber \\  &\quad \le 2\sum _{\begin{array}{c} k\in \mathbb {Z}^{3,N}_0\\ k_3=0,\quad j\in \{1,2\} \end{array}}\langle \overline{B^{H,\varepsilon }_{t}}\cdot \nabla _H\sigma _{k,j}^{3,\varepsilon },\overline{B^{H,\varepsilon }_{3}}\rangle _{\varepsilon }dW^{k,j}_t\nonumber \\  &\quad \quad -2\sum _{\begin{array}{c} k\in \mathbb {Z}^{3,N}_0\\ k_3\ne 0,\quad j\in \{1,2\} \end{array}}\langle \sigma _{k,j}^{\varepsilon }\cdot \nabla {{B}^{3,\varepsilon }_{t}}^{\prime }-{{B}^{\varepsilon }_{t}}^{\prime }\cdot \nabla \sigma _{k,j}^{3,\varepsilon },\overline{B^{3,\varepsilon }_{t}}\rangle _{\varepsilon }dW^{k,j}_t\nonumber \\  &\quad \quad +2\left( \frac{\zeta _{H,\gamma -2}}{N^{\gamma -4}C_{2,H}^2}+|\rho |\xi _3 \frac{\zeta _{H,\gamma -2}}{N^{\gamma -4}C_{2,H}^2}+\sqrt{2}|\rho |\xi _3\frac{\zeta _{H,\gamma /2}}{N^{\gamma -4}C_{1,H}C_{2,H}}+O(N^{3-\gamma })\right) \nonumber \\  &\qquad \left\| \nabla \overline{A^{3,\varepsilon }_{t}}\right\| _{\varepsilon }^2 dt\nonumber \\  &\quad \quad +2\sum _{\begin{array}{c} k\in \mathbb {Z}^{3,N}_0\\ k_3\ne 0,\quad j\in \{1,2\} \end{array}}\left\| \overline{\sigma _{k,j}^{\varepsilon }\cdot \nabla {{B}^{3,\varepsilon }_{t}}^{\prime }-{{B}^{\varepsilon }_{t}}^{\prime }\cdot \nabla \sigma _{k,j}^{3,\varepsilon }}\right\| _{\varepsilon }^2dt \nonumber \\  &\quad \quad +2\frac{|\rho |}{\xi _3} \left( \frac{\zeta _{H,2}}{C_{1,H}^2}+\sqrt{2}\frac{\zeta _{H,\gamma /2}}{C_{1,H}C_{2,H}}+O(N^{-1})\right) \left\| \nabla \overline{B^{3,\varepsilon }_{t}}\right\| _{\varepsilon }^2 dt. \end{aligned}$$

### Proof

First let us observe that thanks to (2.2) and Proposition 3.1

3.25 $$\begin{aligned} \overline{A^{3,\varepsilon }_{t}}&=-\nabla _{H}^{\perp }(-\Delta )^{-1}\overline{B^{H,\varepsilon }_{t}},\quad \left\| \overline{A^{3,\varepsilon }_{t}}\right\| _{\varepsilon }^2= \left\| \left( -\Delta \right) ^{-1/2}\overline{B^{H,\varepsilon }_{t}}\right\| _{\varepsilon }^2,\quad \nonumber \\ \left\| \nabla _H\overline{A^{3,\varepsilon }_{t}}\right\| _{\varepsilon }^2&= \left\| \overline{B^{H,\varepsilon }_{t}}\right\| _{\varepsilon }^2. \end{aligned}$$

Thus we apply Itô formula to $\left\| \left( -\Delta \right) ^{-1/2}\overline{B^{H,\varepsilon }_{t}}\right\| _{\varepsilon }$ obtaining due to Remark A.1

$$\begin{aligned} d\left\| \overline{A^{3,\varepsilon }_{t}}\right\| _{\varepsilon }^2=-d\overline{W}_t+2(-L^1_t+L^2_t+L^3_t+L^4_t)dt, \end{aligned}$$

where we denote by

$$\begin{aligned} d\overline{W}_t&=2\sum _{\begin{array}{c} k\in \mathbb {Z}^{3,N}_0\\ k_3=0,\quad j\in \{1,2\} \end{array}}\langle \sigma _{k,j}^{H,\varepsilon }\cdot \nabla _H\overline{B^{H,\varepsilon }_{t}}-\overline{B^{H,\varepsilon }_{t}}\cdot \nabla _H\sigma _{k,j}^{H,\varepsilon },(-\Delta )^{-1}\overline{B^{H,\varepsilon }_{t}}\rangle _{\varepsilon }dW^{k,j}_t\\  &\quad +2\sum _{\begin{array}{c} k\in \mathbb {Z}^{3,N}_0\\ k_3\ne 0,\quad j\in \{1,2\} \end{array}}\langle \sigma _{k,j}^{\varepsilon }\cdot \nabla {{B}^{H,\varepsilon }_{t}}^{\prime }-{{B}^{\varepsilon }_{t}}^{\prime }\cdot \nabla \sigma _{k,j}^{H,\varepsilon },(-\Delta )^{-1}\overline{B^{H,\varepsilon }_{t}}\rangle _{\varepsilon }dW^{k,j}_t,\\ L^1_t&=\eta \langle \nabla _H\overline{B^{H,\varepsilon }_{t}},(-\Delta )^{-1}\nabla _H\overline{B^{H,\varepsilon }_{t}}\rangle _{\varepsilon }\\  &\quad +\sum _{\begin{array}{c} k\in \mathbb {Z}^{3,N}_0\\ j\in \{1,2\} \end{array}}\langle \overline{B^{H,\varepsilon }_{t}}, (-\Delta )^{-1}\operatorname {div}_H(\sigma _{k,j}^{H,\varepsilon }\otimes \sigma _{-k,j}^{H,\varepsilon } \nabla _H \overline{B^{H,\varepsilon }_{t}})\rangle _{\varepsilon },\\ L^2_t&=\sum _{\begin{array}{c} k\in \mathbb {Z}^{3,N}_0\\ k_3=0,\quad j\in \{1,2\} \end{array}}\left\langle \sigma _{k,j}^{H,\varepsilon }\cdot \nabla _H\overline{B^{H,\varepsilon }_{t}}-\overline{B^{H,\varepsilon }_{t}}\cdot \nabla _H\sigma _{k,j}^{H,\varepsilon },\right. \\  &\left. \quad (-\Delta )^{-1}\left( \sigma _{-k,j}^{H,\varepsilon }\cdot \nabla _H\overline{B^{H,\varepsilon }_{t}}-\overline{B^{H,\varepsilon }_{t}}\cdot \nabla _H\sigma _{-k,j}^{H,\varepsilon }\right) \right\rangle _{\varepsilon }\\ L^3_t&=\sum _{\begin{array}{c} k\in \mathbb {Z}^{3,N}_0\\ k_3\ne 0,\quad j\in \{1,2\} \end{array}}\left\langle \overline{\sigma _{k,j}^{\varepsilon }\cdot \nabla {{B}^{H,\varepsilon }_{t}}^{\prime }-{{B}^{\varepsilon }_{t}}^{\prime }\cdot \nabla \sigma _{k,j}^{H,\varepsilon }} ,\right. \\  &\left. \quad (-\Delta )^{-1}\overline{\sigma _{-k,j}^{\varepsilon }\cdot \nabla {{B}^{H,\varepsilon }_{t}}^{\prime }-{{B}^{\varepsilon }_{t}}^{\prime }\cdot \nabla \sigma _{-k,j}^{H,\varepsilon }} \right\rangle _{\varepsilon }\\ L^4_t&=\rho \sum _{\begin{array}{c} k\in \mathbb {Z}^{3,N}_0\\ k_3=0 \end{array}}\left\langle \sigma _{k,H}^{1,\varepsilon }\cdot \nabla \overline{B^{H,\varepsilon }_{t}}-\overline{B^{H,\varepsilon }_{t}}\cdot \nabla \sigma _{k,1}^{\varepsilon },\right. \\  &\left. \quad (-\Delta )^{-1}\left( \sigma _{-k,H}^{2,\varepsilon }\cdot \nabla \overline{B^{H,\varepsilon }_{t}}-\overline{B^{H,\varepsilon }_{t}}\cdot \nabla \sigma _{-k,2}^{\varepsilon }\right) \right\rangle _{\varepsilon }\\  &\quad +\rho \sum _{\begin{array}{c} k\in \mathbb {Z}^{3,N}_0\\ k_3=0 \end{array}}\left\langle \sigma _{k,H}^{2,\varepsilon }\cdot \nabla \overline{B^{H,\varepsilon }_{t}}-\overline{B^{H,\varepsilon }_{t}}\cdot \nabla \sigma _{k,2}^{\varepsilon },\right. \\  &\left. \quad (-\Delta )^{-1}\left( \sigma _{-k,H}^{1,\varepsilon }\cdot \nabla \overline{B^{H,\varepsilon }_{t}}-\overline{B^{H,\varepsilon }_{t}}\cdot \nabla \sigma _{-k,1}^{\varepsilon }\right) \right\rangle _{\varepsilon }. \end{aligned}$$

Arguing analogously to the first part of Lemma 3.3 we can treat all the terms above. Thanks to (3.4) and (3.5) it holds

3.26 $$\begin{aligned} \sigma _{k,j}^{H,\varepsilon }\cdot \nabla _H\overline{B^{H,\varepsilon }_{t}}-\overline{B^{H,\varepsilon }_{t}}\cdot \nabla \sigma _{k,j}^{H,\varepsilon }&=\nabla _H^{\perp }(\sigma _{k,j}^{H,\varepsilon }\cdot \nabla _H \overline{A^{3,\varepsilon }_{t}})&\text {if } k_3=0,\end{aligned}$$

3.27 $$\begin{aligned} \sigma _{k,j}^{\varepsilon }\cdot \nabla {{B}^{H,\varepsilon }_{t}}^{\prime }-{{B}^{\varepsilon }_{t}}^{\prime }\cdot \nabla \sigma _{k,j}^{H,\varepsilon }&=\left( \operatorname {curl}\left( \sigma _{k,j}^{\varepsilon }\cdot \nabla {{A}^{\varepsilon }_{t}}^{\prime }+D\sigma _{k,j}^{\varepsilon }{{A}^{\varepsilon }_{t}}^{\prime }\right) \right) ^H&\text {if } k_3\ne 0 . \end{aligned}$$

In particular, considering the mean on the third direction of $\sigma _{k,j}^{\varepsilon }\cdot \nabla {{B}^{H,\varepsilon }_{t}}^{\prime }-{{B}^{\varepsilon }_{t}}^{\prime }\cdot \nabla \sigma _{k,j}^{H,\varepsilon }$, we have

3.28 $$\begin{aligned} \overline{\sigma _{k,j}^{\varepsilon }\cdot \nabla {{B}^{H,\varepsilon }_{t}}^{\prime }-{{B}^{\varepsilon }_{t}}^{\prime }\cdot \nabla \sigma _{k,j}^{H,\varepsilon }}&=-\nabla _H^{\perp }\left( \overline{\sigma _{k,j}^{\varepsilon }\cdot \nabla {{A}^{3,\varepsilon }_{t}}^{\prime }+\partial _3\sigma _{k,j}^{\varepsilon }\cdot {{A}^{\varepsilon }_{t}}^{\prime }} \right)&\text {if } k_3\ne 0. \end{aligned}$$

Exploiting these relations we can obtain the required estimates. Let us start analysing $d\overline{W}_t$.

3.29 $$\begin{aligned} d\overline{W}_t&= 2\sum _{\begin{array}{c} k\in \mathbb {Z}^{3,N}_0\\ k_3=0,\quad j\in \{1,2\} \end{array}}\langle \nabla _H^{\perp }(\sigma _{k,j}^{H,\varepsilon }\cdot \nabla _H \overline{A^{3,\varepsilon }_{t}}),(-\Delta )^{-1}\nabla _H^{\perp }\overline{A^{3,\varepsilon }_{t}}\rangle _{\varepsilon }dW^{k,j}_t\nonumber \\  &\quad +2\sum _{\begin{array}{c} k\in \mathbb {Z}^{3,N}_0\\ k_3\ne 0,\quad j\in \{1,2\} \end{array}}\langle \left( \operatorname {curl}\left( \sigma _{k,j}^{\varepsilon }\cdot \nabla {{A}^{\varepsilon }_{t}}^{\prime }+D\sigma _{k,j}^{\varepsilon }{{A}^{\varepsilon }_{t}}^{\prime }\right) \right) ^H,(-\Delta )^{-1}\nabla _H^{\perp }\overline{A^{3,\varepsilon }_{t}}\rangle _{\varepsilon }dW^{k,j}_t \nonumber \\  &=2 \sum _{\begin{array}{c} k\in \mathbb {Z}^{3,N}_0\\ k_3=0,\quad j\in \{1,2\} \end{array}}\langle \sigma _{k,j}^{H,\varepsilon }\cdot \nabla _H \overline{A^{3,\varepsilon }_{t}},\overline{A^{3,\varepsilon }_{t}}\rangle _{\varepsilon }dW^{k,j}_t\nonumber \\  &\quad +2\sum _{\begin{array}{c} k\in \mathbb {Z}^{3,N}_0\\ k_3\ne 0,\quad j\in \{1,2\} \end{array}}\langle \sigma _{k,j}^{\varepsilon }\cdot \nabla {{A}^{3,\varepsilon }_{t}}^{\prime }+\partial _3\sigma _{k,j}^{\varepsilon }\cdot {{A}^{\varepsilon }_{t}}^{\prime },\overline{A^{3,\varepsilon }_{t}}\rangle _{\varepsilon }dW^{k,j}_t\nonumber \\  &=2\sum _{\begin{array}{c} k\in \mathbb {Z}^{3,N}_0\\ k_3\ne 0,\quad j\in \{1,2\} \end{array}}\langle \sigma _{k,j}^{\varepsilon }\cdot \nabla {{A}^{3,\varepsilon }_{t}}^{\prime }+\partial _3\sigma _{k,j}^{\varepsilon }\cdot {{A}^{\varepsilon }_{t}}^{\prime },\overline{A^{3,\varepsilon }_{t}}\rangle _{\varepsilon }dW^{k,j}_t. \end{aligned}$$

Moving to $L^1_t$ we get easily, integrating by parts, that it holds

3.30 $$\begin{aligned} L^1_t&=\eta \left\| \nabla \overline{A^{3,\varepsilon }_{t}}\right\| _{\varepsilon }^2+\sum _{\begin{array}{c} k\in \mathbb {Z}^{3,N}_0\\ j\in \{1,2\} \end{array}}\left\| \sigma _{k,j}^{H,\varepsilon }\cdot \nabla _H\overline{A^{3,\varepsilon }_{t}} \right\| _{\varepsilon }^2. \end{aligned}$$

Concerning $L^2_t$ we obtain by (3.26)

3.31 $$\begin{aligned} L^2_t&=\sum _{\begin{array}{c} k\in \mathbb {Z}^{3,N}_0\\ k_3=0,\quad j\in \{1,2\} \end{array}}\langle \nabla _H^{\perp }(\sigma _{k,j}^{H,\varepsilon }\cdot \nabla _H \overline{A^{3,\varepsilon }_{t}}),(-\Delta )^{-1}\nabla _H^{\perp }(\sigma _{-k,j}^{H,\varepsilon }\cdot \nabla _H \overline{A^{3,\varepsilon }_{t}}) \rangle _{\varepsilon }\nonumber \\  &=\sum _{\begin{array}{c} k\in \mathbb {Z}^{3,N}_0\\ k_3=0,\quad j\in \{1,2\} \end{array}}\left\| \sigma _{k,j}^{H,\varepsilon }\cdot \nabla _H \overline{A^{3,\varepsilon }_{t}}\right\| _{\varepsilon }^2. \end{aligned}$$

Thanks to (3.28) and computations analogous to the ones of Lemma 3.3, we can treat $L^3_t$ obtaining

3.32 $$\begin{aligned} L^3_t&= \sum _{\begin{array}{c} k\in \mathbb {Z}^{3,N}_0\\ k_3\ne 0,\quad j\in \{1,2\} \end{array}}\left\| \overline{\sigma _{k,j}^{\varepsilon }\cdot \nabla {{A}^{3,\varepsilon }_{t}}^{\prime }+\partial _3\sigma _{k,j}^{\varepsilon }\cdot {{A}^{\varepsilon }_{t}}^{\prime }}\right\| _{\varepsilon }^2\nonumber \\  &\le \sum _{\begin{array}{c} k\in \mathbb {Z}^{3,N}_0\\ k_3\ne 0,\quad j\in \{1,2\} \end{array}}\left\| \sigma _{k,j}^{\varepsilon }\cdot \nabla {{A}^{3,\varepsilon }_{t}}^{\prime }+\partial _3\sigma _{k,j}^{\varepsilon }\cdot {{A}^{\varepsilon }_{t}}^{\prime }\right\| _{\varepsilon }^2\nonumber \\  &\le \left( 2\frac{\zeta _{\beta }\zeta _{H,0}}{N^{\beta -2}C_V^2}+O(N^{1- \beta })\right) \left\| \nabla {{A}^{3,\varepsilon }_{t}}^{\prime }\right\| _{\varepsilon }^2+\left( 2\frac{\zeta _{\beta -2}\zeta _{H,0}}{N^{\beta -4}C_V^2}+O(N^{3-\beta })\right) \left\| {{A}^{\varepsilon }_{t}}^{\prime }\right\| _{\varepsilon }^2. \end{aligned}$$

Lastly $L^4_t=0$ thanks to Proposition 3.2. Indeed for each $k\in \mathbb {Z}_0^{3,N}$ such that $k_3=0$ it holds

$$\begin{aligned}&\langle \sigma _{k,H}^{1,\varepsilon }\cdot \nabla \overline{B^{H,\varepsilon }_{t}}-\overline{B^{H,\varepsilon }_{t}}\cdot \nabla \sigma _{k,1}^{\varepsilon },(-\Delta )^{-1}\left( \sigma _{-k,H}^{2,\varepsilon }\cdot \nabla \overline{B^{H,\varepsilon }_{t}}-\overline{B^{H,\varepsilon }_{t}}\cdot \nabla \sigma _{-k,2}^{\varepsilon }\right) \rangle _{\varepsilon }\\  &=\langle \operatorname {curl}\left( \sigma _{k,1}^{\varepsilon }\cdot \nabla \overline{A^{3,\varepsilon }_{t}}\right) ,(-\Delta )^{-1}\operatorname {curl}\left( \sigma _{-k,2}^{\varepsilon }\cdot \nabla \overline{A^{3,\varepsilon }_{t}}\right) \rangle _{\varepsilon }\\  &=\langle \sigma _{k,1}^{\varepsilon }\cdot \nabla \overline{A^{3,\varepsilon }_{t}},\sigma _{-k,2}^{\varepsilon }\cdot \nabla \overline{A^{3,\varepsilon }_{t}}\rangle _{\varepsilon }=0. \end{aligned}$$

Relation (3.23) then follows combining (3.29), (3.30), (3.31) and (3.32).

Now we move to the analysis of $\left\| \overline{B^{3,\varepsilon }_{t}}\right\| _{\varepsilon }^2$ which is similar and easier to the previous one. First by Itô formula we have

$$\begin{aligned} d\left\| \overline{B^{3,\varepsilon }_{t}}\right\| _{\varepsilon }^2=-d\hat{W}_t+2(-H^1_t+H^2_t+H^3_t+H^4_t)dt, \end{aligned}$$

where we denote by

$$\begin{aligned} d\hat{W}_t&=2\sum _{\begin{array}{c} k\in \mathbb {Z}^{3,N}_0\\ k_3=0,\quad j\in \{1,2\} \end{array}}\langle \sigma _{k,j}^{H,\varepsilon }\cdot \nabla _H\overline{B^{3,\varepsilon }_{t}}-\overline{B^{H,\varepsilon }_{t}}\cdot \nabla _H\sigma _{k,j}^{3,\varepsilon },\overline{B^{3,\varepsilon }_{t}}\rangle _{\varepsilon }dW^{k,j}_t\\  &\quad +2\sum _{\begin{array}{c} k\in \mathbb {Z}^{3,N}_0\\ k_3\ne 0,\quad j\in \{1,2\} \end{array}}\langle \sigma _{k,j}^{\varepsilon }\cdot \nabla {{B}^{3,\varepsilon }_{t}}^{\prime }-{{B}^{\varepsilon }_{t}}^{\prime }\cdot \nabla \sigma _{k,j}^{3,\varepsilon },\overline{B^{3,\varepsilon }_{t}}\rangle _{\varepsilon }dW^{k,j}_t,\\ H^1_t&=\eta \left\| \nabla _H\overline{B^{3,\varepsilon }_{t}}\right\| _{\varepsilon }^2+\sum _{\begin{array}{c} k\in \mathbb {Z}^{3,N}_0\\ j\in \{1,2\} \end{array}}\left\| \sigma _{k,j}^{H,\varepsilon }\cdot \nabla _H\overline{B^{3,\varepsilon }_{t}}\right\| _{\varepsilon }^2,\\ H^2_t&=\sum _{\begin{array}{c} k\in \mathbb {Z}^{3,N}_0\\ k_3=0,\quad j\in \{1,2\} \end{array}}\langle \sigma _{k,j}^{H,\varepsilon }\cdot \nabla _H\overline{B^{3,\varepsilon }_{t}}-\overline{B^{H,\varepsilon }_{t}}\cdot \nabla _H\sigma _{k,j}^{3,\varepsilon },\sigma _{-k,j}^{H,\varepsilon }\cdot \\  &\quad \nabla _H\overline{B^{3,\varepsilon }_{t}}-\overline{B^{H,\varepsilon }_{t}}\cdot \nabla _H\sigma _{-k,j}^{3,\varepsilon }\rangle _{\varepsilon },\\ H^3_t&=\sum _{\begin{array}{c} k\in \mathbb {Z}^{3,N}_0\\ k_3\ne 0,\quad j\in \{1,2\} \end{array}}\langle \overline{\sigma _{k,j}^{\varepsilon }\cdot \nabla {{B}^{3,\varepsilon }_{t}}^{\prime }-{{B}^{\varepsilon }_{t}}^{\prime }\cdot \nabla \sigma _{k,j}^{3,\varepsilon }} ,\overline{\sigma _{-k,j}^{\varepsilon }\cdot \nabla {{B}^{3,\varepsilon }_{t}}^{\prime }-{{B}^{\varepsilon }_{t}}^{\prime }\cdot \nabla \sigma _{-k,j}^{3,\varepsilon }} \rangle _{\varepsilon },\\ H^4_t&=-\langle \operatorname {div}_H\left( \mathcal {R}_{\varepsilon ,\gamma } \overline{B^{H,\varepsilon }_{t}}\right) ,\overline{B^{3,\varepsilon }_{t}}\rangle _{\varepsilon } -\rho \sum _{\begin{array}{c} k\in \mathbb {Z}^{3,N}_0\\ k_3=0 \end{array}}\langle \sigma _{k,1}^{\varepsilon }\cdot \nabla \overline{B^{3,\varepsilon }_{t}},\overline{B^{\varepsilon }_{t}}\cdot \nabla \sigma _{-k,2}^{3,\varepsilon }\rangle _{\varepsilon }\\  &\quad +\langle \overline{B^{\varepsilon }_{t}}\cdot \nabla \sigma _{k,2}^{3,\varepsilon } ,\sigma _{-k,1}^{\varepsilon }\cdot \nabla \overline{B^{3,\varepsilon }_{t}}\rangle _{\varepsilon }. \end{aligned}$$

Analogously to the second part of Lemma 3.3 we can treat all the terms above. Let us start with $d\hat{W}_t$. Since the $\sigma _{k,j}^{\varepsilon }$ are divergence free we get immediately

3.33 $$\begin{aligned} d\hat{W}_t&=-2\sum _{\begin{array}{c} k\in \mathbb {Z}^{3,N}_0\\ k_3=0,\quad j\in \{1,2\} \end{array}}\langle \overline{B^{H,\varepsilon }_{t}}\cdot \nabla _H\sigma _{k,j}^{3,\varepsilon },\overline{B^{3,\varepsilon }_{t}}\rangle _{\varepsilon }dW^{k,j}_t \nonumber \\  &\quad +2\sum _{\begin{array}{c} k\in \mathbb {Z}^{3,N}_0\\ k_3\ne 0,\quad j\in \{1,2\} \end{array}}\langle \sigma _{k,j}^{\varepsilon }\cdot \nabla {{B}^{3,\varepsilon }_{t}}^{\prime }-{{B}^{\varepsilon }_{t}}^{\prime }\cdot \nabla \sigma _{k,j}^{3,\varepsilon },\overline{B^{3,\varepsilon }_{t}}\rangle _{\varepsilon }dW^{k,j}_t. \end{aligned}$$

Thanks to (2.18) we get easily by Hölder’s inequality

3.34 $$\begin{aligned} H^2_t&= \sum _{\begin{array}{c} k\in \mathbb {Z}^{3,N}_0\\ k_3=0,\quad j\in \{1,2\} \end{array}}\left\| \sigma _{k,j}^{H,\varepsilon }\cdot \nabla _H\overline{B^{3,\varepsilon }_{t}}\right\| _{\varepsilon }^2+\sum _{\begin{array}{c} k\in \mathbb {Z}^{3,N}_0\\ k_3=0,\quad j\in \{1,2\} \end{array}}\left\| \overline{B^{H,\varepsilon }_{t}}\cdot \nabla _H\sigma _{k,j}^{3,\varepsilon }\right\| _{\varepsilon }^2\nonumber \\  &\le \sum _{\begin{array}{c} k\in \mathbb {Z}^{3,N}_0\\ k_3=0,\quad j\in \{1,2\} \end{array}}\left\| \sigma _{k,j}^{H,\varepsilon }\cdot \nabla _H\overline{B^{3,\varepsilon }_{t}}\right\| _{\varepsilon }^2+\sum _{\begin{array}{c} k\in \mathbb {Z}^{3,N}_0\\ k_3=0,\quad j=2 \end{array}}\left\lvert \theta _{k,j}^{\varepsilon }\right\rvert ^2|k|^2 \left\| \overline{B^{H,\varepsilon }_{t}}\right\| _{\varepsilon }^2\nonumber \\  &\le \sum _{\begin{array}{c} k\in \mathbb {Z}^{3,N}_0\\ k_3=0,\quad j\in \{1,2\} \end{array}}\left\| \sigma _{k,j}^{H,\varepsilon }\cdot \nabla _H\overline{B^{3,\varepsilon }_{t}}\right\| _{\varepsilon }^2+\sum _{\begin{array}{c} k_H\in \mathbb {Z}^2_0\\ N\le |k_H|\le 2N \end{array}}\frac{1}{C_{2,H}^2|k|^{\gamma -2}}\left\| \nabla \overline{A^{3,\varepsilon }_{t}}\right\| _{\varepsilon }^2 \nonumber \\  &\le \sum _{\begin{array}{c} k\in \mathbb {Z}^{3,N}_0\\ k_3=0,\quad j\in \{1,2\} \end{array}}\left\| \sigma _{k,j}^{H,\varepsilon }\cdot \nabla _H\overline{B^{3,\varepsilon }_{t}}\right\| _{\varepsilon }^2+\left( \frac{\zeta _{H,\gamma -2}}{N^{\gamma -4}C_{2,H}^2}+O(N^{3-\gamma })\right) \left\| \nabla \overline{A^{3,\varepsilon }_{t}}\right\| _{\varepsilon }^2. \end{aligned}$$

We rewrite $H_t^3$ as

3.35 $$\begin{aligned} H^3_t&=\sum _{\begin{array}{c} k\in \mathbb {Z}^{3,N}_0\\ k_3\ne 0,\quad j\in \{1,2\} \end{array}}\left\| \overline{\sigma _{k,j}^{\varepsilon }\cdot \nabla {{B}^{3,\varepsilon }_{t}}^{\prime }-{{B}^{\varepsilon }_{t}}^{\prime }\cdot \nabla \sigma _{k,j}^{3,\varepsilon }}\right\| _{\varepsilon }^2. \end{aligned}$$

Lastly let us treat $H^4_t$: the term $-\langle \operatorname {div}_H\left( \mathcal {R}_{\varepsilon ,\gamma } \overline{B^{H,\varepsilon }_{t}}\right) ,\overline{B^{3,\varepsilon }_{t}}\rangle _{\varepsilon }$ can be estimated easily due to Lemma 2.10 and (3.2) obtaining by Cauchy-Schwarz inequality

$$\begin{aligned} -\langle \operatorname {div}_H\left( \mathcal {R}_{\varepsilon ,\gamma } {{B}^{H,\varepsilon }_{t}}^{\prime }\right) ,{{B}^{3,\varepsilon }_{t}}^{\prime }\rangle _{\varepsilon }&= -\langle \mathcal {R}_{\varepsilon ,\gamma }\cdot \nabla ^{\perp }_H\overline{A^{3,\varepsilon }_{t}},\nabla _H\overline{B^{3,\varepsilon }_{t}}\rangle _{\varepsilon }\\  &\le \frac{2\sqrt{2}|\rho |\zeta ^N_{H,\gamma /2} }{N^{\gamma /2-2}C_{1,H}C_{2,H}}\left\| \nabla \overline{B^{3,\varepsilon }_{t}}\right\| _\varepsilon \left\| \nabla \overline{A^{3,\varepsilon }_{t}}\right\| _\varepsilon . \end{aligned}$$

The other terms can be treated easily by Cauchy-Schwartz inequality. Indeed, for each $\xi _3>0$ and $k\in \mathbb {Z}_0^{3,N}$ such that $k_3=0$ it holds

$$\begin{aligned} \langle \sigma _{k,1}^{\varepsilon }\cdot \nabla \overline{B^{3,\varepsilon }_{t}},\overline{B^{\varepsilon }_{t}}\cdot \nabla \sigma _{-k,2}^{3,\varepsilon }\rangle _{\varepsilon }\le \frac{\left\| \sigma _{k,1}^{\varepsilon }\cdot \nabla \overline{B^{3,\varepsilon }_{t}}\right\| _{\varepsilon }^2}{2\xi _3}+\frac{\xi _3 \left\| \overline{B^{H,\varepsilon }_{t}}\cdot \nabla _H\sigma _{k,2}^{3,\varepsilon }\right\| _{\varepsilon }^2}{2}. \end{aligned}$$

In conclusion arguing as when treating $H^2_t$, so far, we have proved that for each $\xi _3>0$

3.36 $$\begin{aligned} H^4_t&\le \frac{2\sqrt{2}|\rho |\zeta ^N_{H,\gamma /2} }{N^{\gamma /2-2}C_{1,H}C_{2,H}}\left\| \nabla \overline{B^{3,\varepsilon }_{t}}\right\| _{\varepsilon }\left\| \nabla \overline{A^{3,\varepsilon }_{t}}\right\| _{\varepsilon }+\frac{|\rho |}{\xi _3} \sum _{\begin{array}{c} k\in \mathbb {Z}^{3,N}_0\\ k_3=0,\quad j=1 \end{array}}\left\| \sigma _{k,j}^{\varepsilon }\cdot \nabla \overline{B^{3,\varepsilon }_{t}}\right\| ^2_{\varepsilon }\nonumber \\  &\quad +|\rho |\xi _3 \left( \frac{\zeta _{H,\gamma -2}}{N^{\gamma -4}C_{2,H}^2}+O(N^{3-\gamma })\right) \left\| \nabla \overline{A^{3,\varepsilon }_{t}}\right\| _{\varepsilon }^2. \end{aligned}$$

Let us analyse $$\sum _{\begin{array}{c} k\in \mathbb {Z}^{3,N}_0\\ k_3=0,\quad j=1 \end{array}}\left\| \sigma _{k,j}^{\varepsilon }\cdot \nabla \overline{B^{3,\varepsilon }_{t}}\right\| ^2_{\varepsilon }$$. Arguing as in the second part of Lemma 3.3 we have

$$\begin{aligned} \sum _{\begin{array}{c} k\in \mathbb {Z}^{3,N}_0\\ k_3=0,\quad j=1 \end{array}}\left\| \sigma _{k,j}^{\varepsilon }\cdot \nabla \overline{B^{3,\varepsilon }_{t}}\right\| ^2_{\varepsilon }&\le \left( \frac{\zeta _{H,2}}{C_{1,H}^2}+O(N^{-1})\right) \left\| \nabla \overline{B^{3,\varepsilon }_{t}}\right\| _{\varepsilon }^2. \end{aligned}$$

In conclusion, applying Young’s inequality to the first term of (3.36), it holds

3.37 $$\begin{aligned} H^4_t&\le \frac{|\rho |}{\xi _3} \left( \frac{\zeta _{H,2}}{C_{1,H}^2}+\sqrt{2}\frac{\zeta _{H,\gamma /2}}{C_{1,H}C_{2,H}}+O(N^{-1})\right) \left\| \nabla \overline{B^{3,\varepsilon }_{t}}\right\| _{\varepsilon }^2\nonumber \\  &\quad +|\rho |\xi _3 \left( \frac{\zeta _{H,\gamma -2}}{N^{\gamma -4}C_{2,H}^2}+\sqrt{2}\frac{\zeta _{H,\gamma /2}}{N^{\gamma -4}C_{1,H}C_{2,H}}+O(N^{3-\gamma })\right) \left\| \nabla \overline{A^{3,\varepsilon }_{t}}\right\| _{\varepsilon }^2. \end{aligned}$$

Combining (3.33), (3.34), (3.35) and (3.37) we get relation (3.24). □

Lemma 3.3 and Lemma 3.4 imply the following version of (2.36):

### Proposition 3.5

Assuming Hypothesis 2.6, for N>0 large enough

3.38 $$\begin{aligned} \mathbb {E} \left[ \sup _{t\in [0,T]}\left\| \overline{A^{3,\varepsilon }_{t}}\right\| _{\varepsilon }^2\right] +\mathbb {E} \left[ \int _0^T\left\| \nabla \overline{A^{3,\varepsilon }_{s}}\right\| _{\varepsilon }^2 ds\right]&\lesssim {\left\| B_0^{3,\varepsilon }\right\| _{\varepsilon }^2}+{\left\| \overline{A^{3,\varepsilon }_{0}}\right\| _{\varepsilon }^2}+{\left\| {{A}^{\varepsilon }_{0}}^{\prime }\right\| _{\varepsilon }^2}. \end{aligned}$$

3.39 $$\begin{aligned} \mathbb {E} \left[ \sup _{t\in [0,T]}\left\| \overline{B^{3,\varepsilon }_{t}}\right\| _{\varepsilon }^2\right] +\mathbb {E} \left[ \int _0^T\left\| \nabla \overline{B^{3,\varepsilon }_{s}}\right\| _{\varepsilon }^2 ds\right]&\lesssim {\left\| B_0^{3,\varepsilon }\right\| _{\varepsilon }^2}+{\left\| \overline{A^{3,\varepsilon }_{0}}\right\| _{\varepsilon }^2}+{\left\| {{A}^{\varepsilon }_{0}}^{\prime }\right\| _{\varepsilon }^2} .\end{aligned}$$

3.40 $$\begin{aligned} \mathbb {E} \left[ \sup _{t\in [0,T]}\left\| {{A}^{\varepsilon }_{t}}^{\prime }\right\| _{\varepsilon }^2\right] +\mathbb {E} \left[ \int _0^T\left\| \nabla {{A}^{\varepsilon }_{s}}^{\prime }\right\| _{\varepsilon }^2 ds\right]&\lesssim {\left\| B_0^{3,\varepsilon }\right\| _{\varepsilon }^2}+{\left\| \overline{A^{3,\varepsilon }_{0}}\right\| _{\varepsilon }^2}+{\left\| {{A}^{\varepsilon }_{0}}^{\prime }\right\| _{\varepsilon }^2}.\end{aligned}$$

3.41 $$\begin{aligned} \mathbb {E} \left[ \sup _{t\in [0,T]}\left\| {{B}^{3,\varepsilon }_{t}}^{\prime }\right\| _{\varepsilon }^2\right] +\mathbb {E} \left[ \int _0^T\left\| \nabla {{B}^{3,\varepsilon }_{s}}^{\prime }\right\| _{\varepsilon }^2 ds\right]&\lesssim {\left\| B_0^{3,\varepsilon }\right\| _{\varepsilon }^2}+{\left\| \overline{A^{3,\varepsilon }_{0}}\right\| _{\varepsilon }^2}+{\left\| {{A}^{\varepsilon }_{0}}^{\prime }\right\| _{\varepsilon }^2}. \end{aligned}$$

The hidden constants above all depend only on $\eta ,\beta ,\gamma ,C_V,C_{1,H},C_{2,H},|\rho |,T$.

### Proof

In order to simplify the notation, we will denote by $C_{par}$ several constants depending on η, |ρ|, γ, β, $C_{1,H}$, $C_{2,H}$, $C_V$, *T*, possibly changing their values line by line but independent of *N* in this proof. Let us introduce a new parameter $\eta _{aux}$ such that

$$\begin{aligned} \frac{\zeta _{H,0}}{C_{1,H}^2}<\eta _{aux}< \eta . \end{aligned}$$

Recalling the discussion at the beginning of Section 3, it is easy to show that for each ε>0 the stochastic integrals appearing in (3.6), (3.7), (3.23), (3.24) are true martingales. Let us start considering the expected value of (3.6). We get for each t∈[0,T]

$$\begin{aligned}&\mathbb {E} \left[ \left\| {{A}^{\varepsilon }_{t}}^{\prime }\right\| _{\varepsilon }^2\right] +2\eta \mathbb {E} \left[ \int _0^t\left\| \nabla {{A}^{\varepsilon }_{s}}^{\prime }\right\| ^2_{\varepsilon }ds\right] \\  &\quad \le {\left\| {{A}^{\varepsilon }_{0}}^{\prime }\right\| _{\varepsilon }^2} +\left( 6\frac{\zeta _{\beta }\zeta _{H,0} }{N^{\beta -2}C_V^2}+O(N^{1-\beta })\right) \mathbb {E} \left[ \int _0^t\Vert \nabla \overline{A^{\varepsilon }_{s}}\Vert _{\varepsilon }^2ds\right] \\  &\qquad +\left( 6\frac{\zeta _{\beta -2}\zeta _{H,0}}{N^{\beta -4}C_V^2}+O(N^{3-\beta })\right) \mathbb {E} \left[ \int _0^t\left\| \overline{A^{\varepsilon }_{s}}\right\| _{\varepsilon }^2 ds\right] \nonumber \\  &\qquad +2\left( \frac{\zeta _{H,0}}{C_{1,H}^2}+\frac{|\rho |}{\xi _1}\frac{\left( \zeta _{H,0}+\zeta _{H,2}\right) }{C_{1,H}^2}+O(N^{-1})\right) \mathbb {E} \left[ \int _0^t\left\| \nabla {{A}^{\varepsilon }_{s}}^{\prime }\right\| ^2_{\varepsilon }ds\right] . \end{aligned}$$

Therefore, if we choose $\xi _1$ large enough, there exists $N_0\in \mathbb {N}$ such that for each $N\ge N_0$ it holds

3.42 $$\begin{aligned} \frac{\zeta _{H,0}}{C_{1,H}^2}+\frac{|\rho |}{\xi _1}\frac{\left( \zeta _{H,0}+\zeta _{H,2}\right) }{C_{1,H}^2}+O(N^{-1})\le \eta _{aux} \end{aligned}$$

and we get

3.43 $$\begin{aligned}&\mathbb {E} \left[ \left\| {{A}^{\varepsilon }_{t}}^{\prime }\right\| _{\varepsilon }^2\right] +(\eta -\eta _{aux})\mathbb {E} \left[ \int _0^t\left\| \nabla {{A}^{\varepsilon }_{s}}^{\prime }\right\| ^2_{\varepsilon }ds\right] \nonumber \\  &\quad \le {\left\| {{A}^{\varepsilon }_{0}}^{\prime }\right\| _{\varepsilon }^2} +\left( 6\frac{\zeta _{\beta }\zeta _{H,0} }{N^{\beta -2}C_V^2}+O(N^{1-\beta })\right) \mathbb {E} \left[ \int _0^t\Vert \nabla \overline{A^{\varepsilon }_{s}}\Vert _{\varepsilon }^2ds\right] \nonumber \\  &\quad \quad +\left( 6\frac{\zeta _{\beta -2}\zeta _{H,0}}{N^{\beta -4}C_V^2}+O(N^{3-\beta })\right) \mathbb {E} \left[ \int _0^t\left\| \overline{A^{\varepsilon }_{s}}\right\| _{\varepsilon }^2 ds\right] . \end{aligned}$$

Now we take the expected value of (3.23) at time t∈[0,T] obtaining by Poincaré inequality (2.6)

$$\begin{aligned}&\mathbb {E} \left[ \left\| \overline{A^{3,\varepsilon }_{t}}\right\| _{\varepsilon }^2\right] +2\eta \mathbb {E} \left[ \int _0^t\left\| \nabla \overline{A^{3,\varepsilon }_{s}}\right\| _{\varepsilon }^2 ds\right] \\  &\quad \le {\left\| \overline{A^{3,\varepsilon }_{0}}\right\| _{\varepsilon }^2}+4\left( \frac{\zeta _{\beta }\zeta _{H,0}}{N^{\beta -2}C_V^2}+\frac{\zeta _{\beta -2}\zeta _{H,0}}{N^{\beta -2}C_V^2}+O(N^{1-\beta })\right) \mathbb {E} \left[ \int _0^t \left\| \nabla {{A}^{\varepsilon }_{s}}^{\prime }\right\| _{\varepsilon }^2 ds\right] . \end{aligned}$$

Plugging (3.43) in the relation above we get

3.44 $$\begin{aligned}&\mathbb {E} \left[ \left\| \overline{A^{3,\varepsilon }_{t}}\right\| _{\varepsilon }^2\right] +2\eta \mathbb {E} \left[ \int _0^t\left\| \nabla \overline{A^{3,\varepsilon }_{s}}\right\| _{\varepsilon }^2 ds\right] \nonumber \\&\quad \le {\left\| \overline{A^{3,\varepsilon }_{0}}\right\| _{\varepsilon }^2}+\left( \frac{C_{par}}{N^{\beta -2}}+O(N^{1-\beta })\right) \nonumber \\  &\quad \quad \times \left( \left\| {{A}^{\varepsilon }_{0}}^{\prime }\right\| _{\varepsilon }^2+\left( \frac{C_{par}}{N^{\beta -2}}+O(N^{1-\beta })\right) \mathbb {E} \left[ \int _0^t\Vert \nabla \overline{A^{\varepsilon }_{s}}\Vert _{\varepsilon }^2ds\right] \right. \nonumber \\  &\qquad \left. +\left( \frac{C_{par}}{N^{\beta -4}}+O(N^{3-\beta })\right) \mathbb {E} \left[ \int _0^t\left\| \overline{A^{\varepsilon }_{s}}\right\| _{\varepsilon }^2 ds\right] \right) . \end{aligned}$$

In order to simplify the expression above, let us observe that since $\overline{A^{\varepsilon }_{t}}$ is independent of the third component, Poincaré inequality with constant equal to 1 holds. Therefore we have

3.45 $$\begin{aligned} \mathbb {E} \left[ \int _0^t\left\| \overline{A^{\varepsilon }_{s}}\right\| _{\varepsilon }^2 ds\right] \le \mathbb {E} \left[ \int _0^t\left\| \nabla \overline{A^{\varepsilon }_{s}}\right\| _{\varepsilon }^2 ds\right] . \end{aligned}$$

Secondly, thanks to Proposition 3.1 it holds

3.46 $$\begin{aligned} \left\| \nabla \overline{A^{\varepsilon }_{t}}\right\| _{\varepsilon }^2&=\left\| \overline{B^{\varepsilon }_{t}}\right\| _{\varepsilon }^2=\left\| \overline{B^{3,\varepsilon }_{t}}\right\| _{\varepsilon }^2+\left\| \overline{B^{H,\varepsilon }_{t}}\right\| _{\varepsilon }^2=\left\| \overline{B^{3,\varepsilon }_{t}}\right\| _{\varepsilon }^2+\left\| \nabla \overline{A^{3,\varepsilon }_{t}}\right\| _{\varepsilon }^2,\end{aligned}$$

3.47 $$\begin{aligned} \left\| \overline{A^{\varepsilon }_{t}}\right\| _{\varepsilon }^2&=\left\| (-\Delta )^{-1/2}\overline{B^{\varepsilon }_{t}}\right\| _{\varepsilon }^2=\left\| (-\Delta )^{-1/2}\overline{B^{3,\varepsilon }_{t}}\right\| _{\varepsilon }^2+\left\| (-\Delta )^{-1/2}\overline{B^{H,\varepsilon }_{t}}\right\| _{\varepsilon }^2\nonumber \\  &\quad \le \left\| \overline{B^{3,\varepsilon }_{t}}\right\| _{\varepsilon }^2+\left\| \overline{A^{3,\varepsilon }_{t}}\right\| _{\varepsilon }^2. \end{aligned}$$

Combining (3.45) and (3.46) in relation (3.44) we get

$$\begin{aligned}&\mathbb {E} \left[ \left\| \overline{A^{3,\varepsilon }_{t}}\right\| _{\varepsilon }^2\right] +2\eta \mathbb {E} \left[ \int _0^t\left\| \nabla \overline{A^{3,\varepsilon }_{s}}\right\| _{\varepsilon }^2 ds\right] \\  &\quad \le {\left\| \overline{A^{3,\varepsilon }_{0}}\right\| _{\varepsilon }^2}+\frac{C_{par}}{N^{2-\beta }}{\left\| {{A}^{\varepsilon }_{0}}^{\prime }\right\| _{\varepsilon }^2} +\left( \frac{C_{par}}{N^{\beta -2}}+O(N^{1-\beta })\right) \mathbb {E} \left[ \int _0^t\Vert \nabla \overline{A^{3,\varepsilon }_{s}}\Vert _{\varepsilon }^2ds\right] \\  &\qquad +\left( \frac{C_{par}}{N^{\beta -2}}+O(N^{1-\beta })\right) \mathbb {E} \left[ \int _0^t\Vert \nabla \overline{B^{3,\varepsilon }_{s}}\Vert _{\varepsilon }^2ds\right] . \end{aligned}$$

Therefore, choosing $N_0\in \mathbb {N}$ large enough such that for each $N\ge N_0$ it holds

$$\begin{aligned} \frac{C_{par}}{N^{\beta -2}}+O(N^{1-\beta })\le \frac{\eta }{2}, \end{aligned}$$

we get

3.48 $$\begin{aligned}&\mathbb {E} \left[ \left\| \overline{A^{3,\varepsilon }_{t}}\right\| _{\varepsilon }^2\right] +\eta \mathbb {E} \left[ \int _0^t\left\| \nabla \overline{A^{3,\varepsilon }_{s}}\right\| _{\varepsilon }^2 ds\right] \nonumber \\  &\quad \le {\left\| \overline{A^{3,\varepsilon }_{0}}\right\| _{\varepsilon }^2}+\frac{C_{par}}{N^{2-\beta }}\left\| {{A}^{\varepsilon }_{0}}^{\prime }\right\| _{\varepsilon }^2\left( \frac{C_{par}}{N^{2-\beta }}+O(N^{1-\beta })\right) \nonumber \\  &\qquad +\mathbb {E} \left[ \int _0^t\Vert \nabla \overline{B^{3,\varepsilon }_{s}}\Vert _{\varepsilon }^2ds\right] . \end{aligned}$$

Now we consider the expected value of (3.7):

3.49 $$\begin{aligned}&\mathbb {E} \left[ \left\| {{B}^{3,\varepsilon }_{t}}^{\prime }\right\| ^2_{\varepsilon }\right] +2\eta \mathbb {E} \left[ \int _0^t\left\| \nabla {{B}^{3,\varepsilon }_{s}}^{\prime }\right\| ^2_{\varepsilon }ds\right] \nonumber \\  &\qquad +2\sum _{\begin{array}{c} k\in \mathbb {Z}^{3,N}_0\\ k_3\ne 0,\quad j\in \{1,2\} \end{array}}\mathbb {E} \left[ \int _0^t\left\| \overline{\sigma _{k,j}^{\varepsilon }\cdot \nabla {{B}^{3,\varepsilon }_{s}}^{\prime }-{{B}^{\varepsilon }_{s}}^{\prime }\cdot \nabla \sigma _{k,j}^{3,\varepsilon }}\right\| _{\varepsilon }^2 ds\right] \nonumber \\  &\qquad \le \left\| {{B}^{3,\varepsilon }_{0}}^{\prime }\right\| ^2_{\varepsilon }+2\frac{|\rho |}{\xi _2}\left( \frac{\zeta _{H,2}}{ C_{1,H}^2}+\sqrt{2}\frac{\zeta _{H,\gamma /2}}{C_{1,H}C_{2,H}}+O(N^{-1})\right) \nonumber \\  &\mathbb {E} \left[ \int _0^t \left\| \nabla {{B}^{3,\varepsilon }_{s}}^{\prime }\right\| _{\varepsilon }^2 ds\right] \nonumber \\  &\qquad +2\left( \frac{\zeta _{H,\gamma -2}}{N^{\gamma -4}C_{2,H}^2}+2\frac{\zeta _{\beta -2}\zeta _{H,0}}{N^{\beta -4}C_V^2}+\frac{|\rho |\xi _2}{N^{\gamma -4}}\left( \frac{\zeta _{H,\gamma -2}}{C_{2,H}^2}+\sqrt{2}\frac{\zeta _{H,\gamma /2}}{C_{1,H}C_{2,H}}\right) \right. \nonumber \\  &\qquad \left. +O(N^{3-\gamma }+N^{3-\beta })\right) \mathbb {E} \left[ \int _0^t \left\| \nabla {{A}^{\varepsilon }_{s}}^{\prime }\right\| ^2_{\varepsilon }ds\right] \nonumber \\  &\qquad +2\sum _{\begin{array}{c} k\in \mathbb {Z}^{3,N}_0\\ k_3\ne 0,\quad j\in \{1,2\} \end{array}}\mathbb {E} \left[ \int _0^t\left\| \sigma _{k,j}^{\varepsilon }\cdot \nabla \overline{B^{3,\varepsilon }_{s}}\right\| ^2_{\varepsilon }ds\right] +\left( 4\frac{\zeta _{\beta -2}\zeta _{H,0}}{N^{\beta -4}C_{V}^2}+O(N^{3-\beta })\right) \nonumber \\  &\mathbb {E} \left[ \int _0^t\left\| \overline{B^{\varepsilon }_{s}}\right\| ^2_{\varepsilon }ds\right] . \end{aligned}$$

Considering $\xi _2>0$ large enough, we can find $N_0\in \mathbb {N}$ such that for each $N\ge N_0$ it holds

$$\begin{aligned} \frac{|\rho |}{\xi _2}\left( \frac{\zeta _{H,2}}{ C_{1,H}^2}+\sqrt{2}\frac{\zeta _{H,\gamma /2}}{C_{1,H}C_{2,H}}+O(N^{-1})\right) \le \frac{\eta }{2}. \end{aligned}$$

Therefore (3.49) reduces to

$$\begin{aligned}&\mathbb {E} \left[ \left\| {{B}^{3,\varepsilon }_{t}}^{\prime }\right\| ^2_{\varepsilon }\right] +\eta \mathbb {E} \left[ \int _0^t\left\| \nabla {{B}^{3,\varepsilon }_{s}}^{\prime }\right\| ^2_{\varepsilon }ds\right] \\  &\qquad +2\sum _{\begin{array}{c} k\in \mathbb {Z}^{3,N}_0\\ k_3\ne 0,\quad j\in \{1,2\} \end{array}}\mathbb {E} \left[ \int _0^t\left\| \overline{\sigma _{k,j}^{\varepsilon }\cdot \nabla {{B}^{3,\varepsilon }_{s}}^{\prime }-{{B}^{\varepsilon }_{s}}^{\prime }\cdot \nabla \sigma _{k,j}^{3,\varepsilon }}\right\| _{\varepsilon }^2 ds\right] \nonumber \\  &\quad \le \left\| {{B}^{3,\varepsilon }_{0}}^{\prime }\right\| ^2_{\varepsilon }+2\sum _{\begin{array}{c} k\in \mathbb {Z}^{3,N}_0\\ k_3\ne 0,\quad j\in \{1,2\} \end{array}}\mathbb {E} \left[ \int _0^t\left\| \sigma _{k,j}^{\varepsilon }\cdot \nabla \overline{B^{3,\varepsilon }_{s}}\right\| ^2_{\varepsilon }ds\right] \\  &\qquad +\left( \frac{C_{par}}{N^{\beta -4}}+O(N^{3-\beta })\right) \mathbb {E} \left[ \int _0^t\left\| \overline{B^{\varepsilon }_{s}}\right\| ^2_{\varepsilon }ds\right] \nonumber \\  &\qquad +\left( C_{par}\left( \frac{1}{N^{\gamma -4}}+\frac{1}{N^{\beta -4}}\right) +O(N^{3-\gamma }+N^{3-\beta })\right) \mathbb {E} \left[ \int _0^t \left\| \nabla {{A}^{\varepsilon }_{s}}^{\prime }\right\| ^2_{\varepsilon }ds\right] \nonumber . \end{aligned}$$

Combining (3.43) and the relation above we get

3.50 $$\begin{aligned}&\mathbb {E} \left[ \left\| {{B}^{3,\varepsilon }_{t}}^{\prime }\right\| ^2_{\varepsilon }\right] +\eta \mathbb {E} \left[ \int _0^t\left\| \nabla {{B}^{3,\varepsilon }_{s}}^{\prime }\right\| ^2_{\varepsilon }ds\right] \nonumber \\  &\qquad +2\sum _{\begin{array}{c} k\in \mathbb {Z}^{3,N}_0\\ k_3\ne 0,\quad j\in \{1,2\} \end{array}}\mathbb {E} \left[ \int _0^t \left\| \overline{\sigma _{k,j}^{\varepsilon }\cdot \nabla {{B}^{3,\varepsilon }_{s}}^{\prime }-{{B}^{\varepsilon }_{s}}^{\prime }\cdot \nabla \sigma _{k,j}^{3,\varepsilon }}\right\| _{\varepsilon }^2 ds\right] \nonumber \\  &\quad \le {\left\| {{B}^{3,\varepsilon }_{0}}^{\prime }\right\| ^2_{\varepsilon }} +2\sum _{\begin{array}{c} k\in \mathbb {Z}^{3,N}_0\\ k_3\ne 0,\quad j\in \{1,2\} \end{array}}\mathbb {E} \left[ \int _0^t\left\| \sigma _{k,j}^{\varepsilon }\cdot \nabla \overline{B^{3,\varepsilon }_{s}}\right\| ^2_{\varepsilon }ds\right] \nonumber \\&\quad \quad +\left( \frac{C_{par}}{N^{\beta -4}}+O(N^{3-\beta })\right) \mathbb {E} \left[ \int _0^t\left\| \overline{B^{\varepsilon }_{s}}\right\| ^2_{\varepsilon }ds\right] \nonumber \\  &\quad \quad +\left( C_{par}\left( \frac{1}{N^{\gamma -4}}+\frac{1}{N^{\beta -4}}\right) +O(N^{3-\gamma }+N^{3-\beta })\right) \times \nonumber \\  &\quad \quad \left( {\left\| {{A}^{\varepsilon }_{0}}^{\prime }\right\| _{\varepsilon }^2} +\left( \frac{C_{par}}{N^{\beta -2}}+O(N^{1-\beta })\right) \mathbb {E} \left[ \int _0^t\Vert \nabla \overline{A^{\varepsilon }_{s}}\Vert _{\varepsilon }^2ds\right] \right. \nonumber \\  &\qquad \left. +\left( \frac{C_{par}}{N^{\beta -4}}+O(N^{3-\beta })\right) \mathbb {E} \left[ \int _0^t\left\| \overline{A^{\varepsilon }_{s}}\right\| _{\varepsilon }^2 ds\right] \right) . \end{aligned}$$

Combining (3.50), (3.45), (3.46) and (3.47) we obtain

3.51 $$\begin{aligned}&\mathbb {E} \left[ \left\| {{B}^{3,\varepsilon }_{t}}^{\prime }\right\| ^2_{\varepsilon }\right] +\eta \mathbb {E} \left[ \int _0^t\left\| \nabla {{B}^{3,\varepsilon }_{s}}^{\prime }\right\| ^2_{\varepsilon }ds\right] \nonumber \\  &\qquad +2\sum _{\begin{array}{c} k\in \mathbb {Z}^{3,N}_0\\ k_3\ne 0,\quad j\in \{1,2\} \end{array}}\mathbb {E} \left[ \int _0^t\left\| \overline{\sigma _{k,j}^{\varepsilon }\cdot \nabla {{B}^{3,\varepsilon }_{s}}^{\prime }-{{B}^{\varepsilon }_{s}}^{\prime }\cdot \nabla \sigma _{k,j}^{3,\varepsilon }}\right\| _{\varepsilon }^2 ds\right] \nonumber \\  &\quad \le {\left\| {{B}^{3,\varepsilon }_{0}}^{\prime }\right\| ^2_{\varepsilon }}+ C_{par}\left( \frac{1}{N^{\gamma -4}}+\frac{1}{N^{\beta -4}}\right) {\left\| {{A}^{\varepsilon }_{0}}^{\prime }\right\| _{\varepsilon }^2} \nonumber \\  &\qquad +2\sum _{\begin{array}{c} k\in \mathbb {Z}^{3,N}_0\\ k_3\ne 0,\quad j\in \{1,2\} \end{array}}\mathbb {E} \left[ \int _0^t\left\| \sigma _{k,j}^{\varepsilon }\cdot \nabla \overline{B^{3,\varepsilon }_{s}}\right\| ^2_{\varepsilon }ds\right] \nonumber \\&\quad \quad +\left( C_{par}\left( \frac{1}{N^{\gamma -4}}+\frac{1}{N^{\beta -4}}\right) +O(N^{3-\beta }+N^{3-\gamma })\right) \nonumber \\  &\quad \quad \times \left( \mathbb {E} \left[ \int _0^t \left\| \overline{B^{3,\varepsilon }_{s}}\right\| _{\varepsilon }^2 ds\right] +\mathbb {E} \left[ \int _0^t \left\| \nabla \overline{A^{3,\varepsilon }_{s}}\right\| _{\varepsilon }^2 ds\right] +\mathbb {E} \left[ \int _0^t \left\| \overline{A^{3,\varepsilon }_{s}}\right\| _{\varepsilon }^2 ds\right] \right) . \end{aligned}$$

Therefore, thanks to (3.48) we get

3.52 $$\begin{aligned}&\mathbb {E} \left[ \left\| {{B}^{3,\varepsilon }_{t}}^{\prime }\right\| ^2_{\varepsilon }\right] +2\eta \mathbb {E} \left[ \int _0^t\left\| \nabla {{B}^{3,\varepsilon }_{s}}^{\prime }\right\| ^2_{\varepsilon }ds\right] \nonumber \\  &\qquad +2\sum _{\begin{array}{c} k\in \mathbb {Z}^{3,N}_0\\ k_3\ne 0,\quad j\in \{1,2\} \end{array}}\mathbb {E} \left[ \int _0^t \left\| \overline{\sigma _{k,j}^{\varepsilon }\cdot \nabla {{B}^{3,\varepsilon }_{s}}^{\prime }-{{B}^{\varepsilon }_{s}}^{\prime }\cdot \nabla \sigma _{k,j}^{3,\varepsilon }}\right\| _{\varepsilon }^2 ds\right] \nonumber \\  &\quad \le {\left\| {{B}^{3,\varepsilon }_{0}}^{\prime }\right\| ^2_{\varepsilon }}+C_{par}\left( \frac{1}{N^{\gamma -4}}+\frac{1}{N^{\beta -4}}\right) \left( {\left\| {{A}^{\varepsilon }_{0}}^{\prime }\right\| _{\varepsilon }^2}+{\left\| \overline{A^{3,\varepsilon }_{0}}\right\| _{\varepsilon }^2} \right) \nonumber \\  &\qquad +2\sum _{\begin{array}{c} k\in \mathbb {Z}^{3,N}_0\\ k_3\ne 0,\quad j\in \{1,2\} \end{array}}\mathbb {E} \left[ \int _0^t\left\| \sigma _{k,j}^{\varepsilon }\cdot \nabla \overline{B^{3,\varepsilon }_{s}}\right\| ^2_{\varepsilon }ds\right] \nonumber \\&\quad \quad +\left( C_{par}\left( \frac{1}{N^{\gamma -4}}+\frac{1}{N^{\beta -4}}\right) +O(N^{3-\gamma }+N^{3-\beta })\right) \mathbb {E} \left[ \int _0^t \left\| \overline{B^{3,\varepsilon }_{s}}\right\| _{\varepsilon }^2 ds\right] \nonumber \\  &\quad \quad +\left( \frac{C_{par}}{N^{2-\beta }}+O(N^{1-\beta }) \right) \mathbb {E} \left[ \int _0^t\Vert \nabla \overline{B^{3,\varepsilon }_{s}}\Vert _{\varepsilon }^2ds\right] . \end{aligned}$$

Lastly we take the expected value of (3.24) obtaining

3.53 $$\begin{aligned}&\mathbb {E} \left[ \left\| \overline{B^{3,\varepsilon }_{t}}\right\| _{\varepsilon }^2\right] +2\eta \mathbb {E} \left[ \int _0^t\left\| \nabla \overline{B^{3,\varepsilon }_{s}}\right\| _{\varepsilon }^2 ds\right] \nonumber \\  &\qquad +2\sum _{\begin{array}{c} k\in \mathbb {Z}^{3,N}_0\\ k_3\ne 0,\quad j\in \{1,2\} \end{array}}\mathbb {E} \left[ \int _0^t\left\| \sigma _{k,j}^{\varepsilon }\cdot \nabla \overline{B^{3,\varepsilon }_{s}} \right\| _{\varepsilon }^2 ds\right] \nonumber \\  &\quad \le \left\| \overline{B^{3,\varepsilon }_{0}}\right\| _{\varepsilon }^2+ 2\left( \frac{\zeta _{H,\gamma -2}}{N^{\gamma -4}C_{2,H}^2}+|\rho |\xi _3 \frac{\zeta _{H,\gamma -2}}{N^{\gamma -4}C_{2,H}^2} \right. \nonumber \\  &\qquad \left. +\sqrt{2}|\rho |\xi _3\frac{\zeta _{H,\gamma /2}}{N^{\gamma -4}C_{1,H}C_{2,H}}+O(N^{3-\gamma })\right) \mathbb {E} \left[ \left\| \nabla \overline{A^{3,\varepsilon }_{s}}\right\| _{\varepsilon }^2 ds\right] \nonumber \\  &\quad \quad +2\frac{|\rho |}{\xi _3} \left( \frac{\zeta _{H,2}}{C_{1,H}^2}+\sqrt{2}\frac{\zeta _{H,\gamma /2}}{C_{1,H}C_{2,H}}+O(N^{-1})\right) \mathbb {E} \left[ \int _0^t \left\| \nabla \overline{B^{3,\varepsilon }_{s}}\right\| _{\varepsilon }^2 ds\right] \nonumber \\  &\quad \quad +2\sum _{\begin{array}{c} k\in \mathbb {Z}^{3,N}_0\\ k_3\ne 0,\quad j\in \{1,2\} \end{array}}\mathbb {E} \left[ \int _0^t\left\| \overline{\sigma _{k,j}^{\varepsilon }\cdot \nabla {{B}^{3,\varepsilon }_{s}}^{\prime }-{{B}^{\varepsilon }_{s}}^{\prime }\cdot \nabla \sigma _{k,j}^{3,\varepsilon }}\right\| _{\varepsilon }^2ds\right] . \end{aligned}$$

Considering $\xi _3>0$ large enough we can find $N_0\in \mathbb {N}$ such that for each $N\ge N_0$ it holds

$$\begin{aligned} \frac{|\rho |}{\xi _3} \left( \frac{\zeta _{H,2}}{C_{1,H}^2}+\sqrt{2}\frac{\zeta _{H,\gamma /2}}{C_{1,H}C_{2,H}}+O(N^{-1})\right) \le \frac{\eta }{2}. \end{aligned}$$

Therefore (3.53) reduces to

$$\begin{aligned}&\mathbb {E} \left[ \left\| \overline{B^{3,\varepsilon }_{t}}\right\| _{\varepsilon }^2\right] +\eta \mathbb {E} \left[ \int _0^t\left\| \nabla \overline{B^{3,\varepsilon }_{s}}\right\| _{\varepsilon }^2 ds\right] +2\sum _{\begin{array}{c} k\in \mathbb {Z}^{3,N}_0\\ k_3\ne 0,\quad j\in \{1,2\} \end{array}}\mathbb {E} \left[ \int _0^t\left\| \sigma _{k,j}^{\varepsilon }\cdot \nabla \overline{B^{3,\varepsilon }_{s}} \right\| _{\varepsilon }^2 ds\right] \\  &\quad \le \left\| \overline{B^{3,\varepsilon }_{0}}\right\| _{\varepsilon }^2+\left( \frac{C_{par}}{N^{\gamma -4}}+O(N^{3-\gamma })\right) \mathbb {E} \left[ \left\| \nabla \overline{A^{3,\varepsilon }_{s}}\right\| _{\varepsilon }^2 ds\right] \\  &\qquad +2\sum _{\begin{array}{c} k\in \mathbb {Z}^{3,N}_0\\ k_3\ne 0,\quad j\in \{1,2\} \end{array}}\mathbb {E} \left[ \int _0^t\left\| \overline{\sigma _{k,j}^{\varepsilon }\cdot \nabla {{B}^{3,\varepsilon }_{s}}^{\prime }-{{B}^{\varepsilon }_{s}}^{\prime }\cdot \nabla \sigma _{k,j}^{3,\varepsilon }}\right\| _{\varepsilon }^2ds\right] . \end{aligned}$$

Combining relations (3.48) and (3.52) in the formula above we obtain

$$\begin{aligned}&\mathbb {E} \left[ \left\| \overline{B^{3,\varepsilon }_{t}}\right\| _{\varepsilon }^2\right] +\eta \mathbb {E} \left[ \int _0^t\left\| \nabla \overline{B^{3,\varepsilon }_{s}}\right\| _{\varepsilon }^2ds \right] \\  &\quad \le {\left\| \overline{B^{3,\varepsilon }_{0}}\right\| _{\varepsilon }^2}+{\left\| {{B}^{3,\varepsilon }_{0}}^{\prime }\right\| ^2_{\varepsilon }}+C_{par}\left( \frac{1}{N^{\gamma -4}}+\frac{1}{N^{\beta -4}}\right) \left( {\left\| {{A}^{\varepsilon }_{0}}^{\prime }\right\| _{\varepsilon }^2}+{\left\| \overline{A^{3,\varepsilon }_{0}}\right\| _{\varepsilon }^2} \right) \nonumber \\&\qquad +\left( C_{par}\left( \frac{1}{N^{\gamma -4}}+\frac{1}{N^{\beta -4}}\right) +O(N^{3-\gamma }+N^{3-\beta })\right) \mathbb {E} \left[ \int _0^t \left\| \overline{B^{3,\varepsilon }_{s}}\right\| _{\varepsilon }^2 ds\right] \nonumber \\  &\qquad +\left( \frac{C_{par}}{N^{\beta -2}}+O(N^{1-\beta })\right) \mathbb {E} \left[ \int _0^t\Vert \nabla \overline{B^{3,\varepsilon }_{s}}\Vert _{\varepsilon }^2ds\right] . \end{aligned}$$

Choosing $N_0\in \mathbb {N}$ large enough such that for each $N\ge N_0$ it holds

$$\begin{aligned} \frac{C_{par}}{N^{\beta -2}}+O(N^{1-\beta }) \le \frac{\eta }{2}. \end{aligned}$$

Analogously, the term which multiplies $\mathbb {E} \left[ \int _0^t\Vert \nabla \overline{B^{3,\varepsilon }_{s}}\Vert _{\varepsilon }^2ds\right]$ and the initial conditions can be bounded uniformly in *N* depending only on $\eta , \beta ,\gamma , C_V,C_{1,H}, C_{2,H},|\rho |, T$. Therefore previous expression simplifies and becomes, due to Lemma 2.3,

3.54 $$\begin{aligned}&\mathbb {E} \left[ \left\| \overline{B^{3,\varepsilon }_{t}}\right\| _{\varepsilon }^2\right] +\eta \mathbb {E} \left[ \int _0^t\left\| \nabla \overline{B^{3,\varepsilon }_{s}}\right\| _{\varepsilon }^2ds \right] \nonumber \\&\quad \lesssim _{\eta ,\beta ,\gamma ,C_V,C_{1,H},C_{2,H},|\rho |,T} {\left\| \overline{B^{3,\varepsilon }_{0}}\right\| _{\varepsilon }^2}+{\left\| {{B}^{3,\varepsilon }_{0}}^{\prime }\right\| ^2_{\varepsilon }}+\left( \frac{1}{N^{\beta -4}}+\frac{1}{N^{\gamma -4}}\right) \nonumber \\  &\quad \left( {\left\| \overline{A^{3,\varepsilon }_{0}}\right\| _{\varepsilon }^2}{+}{\left\| {{A}^{\varepsilon }_{0}}^{\prime }\right\| _{\varepsilon }^2}{+}\mathbb {E} \left[ \int _0^t \left\| \overline{B^{3,\varepsilon }_{s}}\right\| _{\varepsilon }^2 ds\right] \right) . \end{aligned}$$

Applying Grownall Lemma to (3.54) we obtain that for each t∈[0,T] it holds

3.55 $$\begin{aligned}&\mathbb {E} \left[ \left\| \overline{B^{3,\varepsilon }_{t}}\right\| _{\varepsilon }^2\right] +\mathbb {E} \left[ \int _0^t\left\| \nabla \overline{B^{3,\varepsilon }_{s}}\right\| _{\varepsilon }^2ds \right] \nonumber \\&\quad \lesssim _{\eta ,\beta ,\gamma ,C_V,C_{1,H},C_{2,H},|\rho |,T} {\left\| B^{3,\varepsilon }_0\right\| _{\varepsilon }^2}\nonumber \\  &\quad +{\left\| \overline{A^{3,\varepsilon }_{0}}\right\| _{\varepsilon }^2}+{\left\| {{A}^{\varepsilon }_{0}}^{\prime }\right\| _{\varepsilon }^2}. \end{aligned}$$

Exploiting the estimates implied by (3.55) in (3.48) and (3.52), we get

3.56 $$\begin{aligned}&\mathbb {E} \left[ \left\| \overline{A^{3,\varepsilon }_{t}}\right\| _{\varepsilon }^2\right] +\mathbb {E} \left[ \int _0^t\left\| \nabla \overline{A^{3,\varepsilon }_{s}}\right\| _{\varepsilon }^2ds \right] \nonumber \\&\quad \lesssim _{\eta ,\beta ,\gamma ,C_V,C_{1,H},C_{2,H},|\rho |,T} {\left\| B^{3,\varepsilon }_0\right\| _{\varepsilon }^2} +{\left\| \overline{A^{3,\varepsilon }_{0}}\right\| _{\varepsilon }^2}+{\left\| {{A}^{\varepsilon }_{0}}^{\prime }\right\| _{\varepsilon }^2},\end{aligned}$$

3.57 $$\begin{aligned}&\mathbb {E} \left[ \left\| {{B}^{3,\varepsilon }_{t}}^{\prime }\right\| _{\varepsilon }^2\right] +\mathbb {E} \left[ \int _0^t\left\| \nabla {{B}^{3,\varepsilon }_{s}}^{\prime }\right\| _{\varepsilon }^2ds \right] \nonumber \\&\quad \lesssim _{\eta ,\beta ,\gamma ,C_V,C_{1,H},C_{2,H},|\rho |,T} {\left\| B^{3,\varepsilon }_0\right\| _{\varepsilon }^2}{\left\| \overline{A^{3,\varepsilon }_{0}}\right\| _{\varepsilon }^2}+{\left\| {{A}^{\varepsilon }_{0}}^{\prime }\right\| _{\varepsilon }^2}. \end{aligned}$$

Combining (3.46), (3.47), (3.55) and (3.56) we get

3.58 $$\begin{aligned}&\mathbb {E} \left[ \left\| {{A}^{\varepsilon }_{t}}^{\prime }\right\| _{\varepsilon }^2\right] +\mathbb {E} \left[ \int _0^t\left\| \nabla {{A}^{\varepsilon }_{s}}^{\prime }\right\| _{\varepsilon }^2ds \right] \nonumber \\&\quad \lesssim _{\eta ,\beta ,\gamma ,C_V,C_{1,H},C_{2,H},|\rho |,T} {\left\| B^{3,\varepsilon }_0\right\| _{\varepsilon }^2}+{\left\| \overline{A^{3,\varepsilon }_{0}}\right\| _{\varepsilon }^2}+{\left\| {{A}^{\varepsilon }_{0}}^{\prime }\right\| _{\varepsilon }^2}. \end{aligned}$$

We omit the easy details in order to obtain (3.56), (3.57) and (3.58). Having already shown (3.55), (3.56), (3.57), (3.58), in order to conclude the proof we need only to put the supremum in time inside the expected value. Since this is a standard procedure we do it only ${{A}^{\varepsilon }_{t}}^{\prime }$, the other being analogous. Considering the expected value of the supremum in time of (3.6) for $\xi _1=1$ we obtain, thanks to (3.55) and (3.56)

3.59 $$\begin{aligned}&\mathbb {E} \left[ \operatorname {sup}_{t\in [0,T]}\left\| {{A}^{\varepsilon }_{t}}^{\prime }\right\| _{\varepsilon }^2\right] \le {\left\| {{A}^{\varepsilon }_{0}}^{\prime }\right\| _{\varepsilon }^2}+2\left( \frac{\zeta _{H,0}}{C_{1,H}^2}+|\rho |\frac{\left( \zeta _{H,0}+\zeta _{H,2}\right) }{C_{1,H}^2}+O(N^{-1})\right) \nonumber \\  &\quad \mathbb {E} \left[ \int _0^T\left\| \nabla {{A}^{\varepsilon }_{s}}^{\prime }\right\| ^2_{\varepsilon }ds\right] \nonumber \\  &\quad +\left( 6\frac{\zeta _{\beta }\zeta _{H,0} }{N^{\beta -2}C_V^2}+O(N^{1-\beta })\right) \mathbb {E} \left[ \int _0^T\Vert \nabla \overline{A^{\varepsilon }_{s}}\Vert _{\varepsilon }^2ds\right] \nonumber \\  &\quad +\left( 6\frac{\zeta _{\beta -2}\zeta _{H,0}}{N^{\beta -4}C_V^2}+O(N^{3-\beta })\right) \mathbb {E} \left[ \int _0^T\left\| \overline{A^{\varepsilon }_{s}}\right\| _{\varepsilon }^2 ds\right] \nonumber \\  &\quad +\mathbb {E} \left[ \operatorname {sup}_{t\in [0,T]}|W'_t|\right] \nonumber \\  &\le C_{par} \left( {\left\| B_0^{3,\varepsilon }\right\| _{\varepsilon }^2}+{\left\| \overline{A^{3,\varepsilon }_{0}}\right\| _{\varepsilon }^2}+{\left\| {{A}^{\varepsilon }_{0}}^{\prime }\right\| _{\varepsilon }^2}\right) +\mathbb {E} \left[ \operatorname {sup}_{t\in [0,T]}|W'_t|\right] , \end{aligned}$$

$W'_t$ being defined in the first part of Lemma 3.3. Therefore we are left to analyse $\mathbb {E} \left[ \operatorname {sup}_{t\in [0,T]}|W'_t|\right]$ which can be treated by Burkholder-Davis-Gundy inequality, Hölder’s and Young’s inequalities obtaining

3.60 $$\begin{aligned}&\mathbb {E} \left[ \operatorname {sup}_{t\in [0,T]}|W'_t|\right] \lesssim _{|\rho |} \mathbb {E} \left[ \left( \sum _{\begin{array}{c} k\in \mathbb {Z}^{3,N}_0\\ j\in \{1,2\} \end{array}}\int _0^T \langle D\sigma _{k,j}^{\varepsilon }{{A}^{\varepsilon }_{t}}^{\prime },{{A}^{\varepsilon }_{t}}^{\prime } \rangle ^2_{\varepsilon }dt\right) ^{1/2}\right] \nonumber \\&\quad + \mathbb {E} \left[ \left( \sum _{\begin{array}{c} k\in \mathbb {Z}^{3,N}_0\\ k_3\ne 0,\quad j\in \{1,2\} \end{array}}\int _0^T \langle \sigma _{k,j}^{\varepsilon }\cdot \nabla \overline{A^{\varepsilon }_{t}}+D\sigma _{k,j}^{\varepsilon }\overline{A^{\varepsilon }_{t}},{{A}^{\varepsilon }_{t}}^{\prime } \rangle ^2_{\varepsilon }dt\right) ^{1/2}\right] \nonumber \\  &\le \mathbb {E} \left[ \operatorname {sup}_{t\in [0,T]}\left\| {{A}^{\varepsilon }_{t}}^{\prime }\right\| _\varepsilon \left( \sum _{\begin{array}{c} k\in \mathbb {Z}^{3,N}_0\\ j\in \{1,2\} \end{array}}\int _0^T \left\| D\sigma _{k,j}^{\varepsilon }{{A}^{\varepsilon }_{t}}^{\prime }\right\| ^2_{\varepsilon }dt\right) ^{1/2}\right] \nonumber \\  &\quad + \mathbb {E} \left[ \operatorname {sup}_{t\in [0,T]}\left\| {{A}^{\varepsilon }_{t}}^{\prime }\right\| _\varepsilon \left( \sum _{\begin{array}{c} k\in \mathbb {Z}^{3,N}_0\\ k_3\ne 0,\quad j\in \{1,2\} \end{array}}\int _0^T \left\| \sigma _{k,j}^{\varepsilon }\cdot \nabla \overline{A^{\varepsilon }_{t}}+D\sigma _{k,j}^{\varepsilon }\overline{A^{\varepsilon }_{t}} \right\| ^2_{\varepsilon }dt\right) ^{1/2}\right] \nonumber \\  &\le \frac{1}{2}\mathbb {E} \left[ \operatorname {sup}_{t\in [0,T]}\left\| {{A}^{\varepsilon }_{t}}^{\prime }\right\| _{\varepsilon }^2\right] + C_{par}\mathbb {E} \left[ \sum _{\begin{array}{c} k\in \mathbb {Z}^{3,N}_0\\ j\in \{1,2\} \end{array}}\int _0^T \left\| D\sigma _{k,j}^{\varepsilon }{{A}^{\varepsilon }_{t}}^{\prime }\right\| ^2_{\varepsilon }dt\right] \nonumber \\  &\quad + C_{par}\mathbb {E} \left[ \sum _{\begin{array}{c} k\in \mathbb {Z}^{3,N}_0\\ k_3\ne 0,\quad j\in \{1,2\} \end{array}}\int _0^T \left\| \sigma _{k,j}^{\varepsilon }\cdot \nabla \overline{A^{\varepsilon }_{t}}+D\sigma _{k,j}^{\varepsilon }\overline{A^{\varepsilon }_{t}}\right\| ^2_{\varepsilon }dt\right] \nonumber \\  &\le C_{par} \left( {\left\| B_0^{3,\varepsilon }\right\| _{\varepsilon }^2}+{\left\| \overline{A^{3,\varepsilon }_{0}}\right\| _{\varepsilon }^2}+{\left\| {{A}^{\varepsilon }_{0}}^{\prime }\right\| _{\varepsilon }^2}\right) +\frac{1}{2}\mathbb {E} \left[ \operatorname {sup}_{t\in [0,T]}\left\| {{A}^{\varepsilon }_{t}}^{\prime }\right\| _{\varepsilon }^2\right] , \end{aligned}$$

where in the last step we combine the computations in order to obtain (3.11), (3.12), (3.13) and the bounds (3.55), (3.56), (3.58). Combining relations (3.59) and (3.60), the a priori bound (3.40) follows. □

As a corollary of (3.38), (3.39) we get

### Corollary 3.6

Assuming Hypothesis 2.6 and there exists $N_0\in \mathbb {N}$ such that for each $N\ge N_0$ it holds

3.61 $$\begin{aligned} {\left\| B_0^{3,\varepsilon }\right\| _{\varepsilon }^2}+{\left\| \overline{A^{3,\varepsilon }_{0}}\right\| _{\varepsilon }^2}+{\left\| {{A}^{\varepsilon }_{0}}^{\prime }\right\| _{\varepsilon }^2}\lesssim \varepsilon , \end{aligned}$$

then there exists $N_1\in \mathbb {N}$ large enough such that for each $N\ge N_1$ we have3.62where the hidden constants depend only on $\eta ,\beta ,\gamma ,C_V,C_{1,H},C_{2,H},|\rho |,T$

### Proof

The thesis follows immediately combining Proposition 3.5 and assumption (3.61). □

## Proof of Theorem 2.15

Let $\kappa _{\varepsilon }:=\eta +\eta ^{\varepsilon }_T\ge \eta >0$ and let us introduce the following notation

$$\begin{aligned} Z^{1,\varepsilon }_t&=\sum _{\begin{array}{c} k\in \mathbb {Z}^{3,N}_0\\ k_3=0,\quad j\in \{1,2\} \end{array}}\int _0^t e^{(t-s)\kappa _{\varepsilon }\Delta }\left( \sigma _{k,j}^{\varepsilon }\cdot \nabla \overline{B^{3,\varepsilon }_{s}}-\overline{B^{\varepsilon }_{s}}\cdot \nabla \sigma _{k,j}^{3,\varepsilon }\right) dW^{k,j}_s\\  &\quad +\sum _{\begin{array}{c} k\in \mathbb {Z}^{3,N}_0\\ k_3\ne 0,\quad j\in \{1,2\} \end{array}}\int _0^t e^{(t-s)\kappa _{\varepsilon }\Delta } \overline{\sigma _{k,j}^{\varepsilon }\cdot \nabla {{B}^{3,\varepsilon }_{s}}^{\prime }-{{B}^{\varepsilon }_{s}}^{\prime }\cdot \nabla \sigma _{k,j}^{3,\varepsilon }} dW^{k,j}_s, \\ Z^{2,\varepsilon }_t&=\sum _{\begin{array}{c} k\in \mathbb {Z}^{3,N}_0\\ k_3=0,\quad j\in \{1,2\} \end{array}}\int _0^t e^{(t-s)\kappa _{\varepsilon }\Delta }\sigma _{k,j}^{\varepsilon }\cdot \nabla \overline{A^{3,\varepsilon }_{s}}dW^{k,j}_s\\  &\quad +\sum _{\begin{array}{c} k\in \mathbb {Z}^{3,N}_0\\ k_3\ne 0,\quad j\in \{1,2\} \end{array}}\int _0^t e^{(t-s)\kappa _{\varepsilon }\Delta }Q\left[ \overline{\sigma _{k,j}^{\varepsilon }\cdot \nabla {{A}^{3,\varepsilon }_{s}}^{\prime }+\partial _3\sigma _{k,j}^{\varepsilon }\cdot {{A}^{\varepsilon }_{t}}^{\prime }}\right] dW^{k,j}_s. \end{aligned}$$

The stochastic integrals above are well defined thanks to the regularity properties of $\overline{B^{\varepsilon }_{t}},\ {{B}^{\varepsilon }_{t}}^{\prime },\overline{A^{\varepsilon }_{t}},{{A}^{\varepsilon }_{t}}^{\prime }$ and the summability properties of the coefficients in Hypothesis 2.6. The first step in order to prove Theorem 2.15 is to rewrite $\overline{B^{3,\varepsilon }_{t}}$ and $\overline{A^{3,\varepsilon }_{t}}$ in mild form. Indeed, recalling the definition of $\mathcal {R}_{\varepsilon ,\gamma }$, see (2.19), the following lemma holds true:

### Lemma 4.1

The following mild formulas hold true

4.1 $$\begin{aligned} \overline{A^{3,\varepsilon }_{t}}&=e^{t\kappa _{\varepsilon }\Delta }\overline{A^{3,\varepsilon }_{0}}-Z^{2,\varepsilon }_t,\end{aligned}$$

4.2 $$\begin{aligned} \overline{B^{3,\varepsilon }_{t}}&=e^{t\kappa _{\varepsilon }\Delta }\overline{B^{3,\varepsilon }_{0}}-Z^{1,\varepsilon }_t+\int _0^t e^{(t-s)\kappa _{\varepsilon }\Delta }\operatorname {div}\left( \mathcal {R}_{\varepsilon ,\gamma }\nabla ^{\perp }\overline{A^{3,\varepsilon }_{s}}\right) ds. \end{aligned}$$

### Proof

Let us take $\phi =(-\nabla ^{\perp }_H(-\Delta )^{-1}\psi _h,0),\ \psi _h=e^{ih\cdot x}$ as test function for (2.28) obtaining, since all the functions appearing in the integrals are independent of $x_3$, that it holds

4.3 $$\begin{aligned} \langle \overline{B^{H,\varepsilon }_{t}},-\nabla ^{\perp }_H(-\Delta )^{-1}\psi _h\rangle&= \langle \overline{B^{H,\varepsilon }_{0}},-\nabla ^{\perp }_H(-\Delta )^{-1}\psi _h\rangle + \kappa _{\varepsilon }\int _0^t \langle \overline{B^{H,\varepsilon }_{s}},\nabla _{H}^{\perp }\psi _h\rangle \, ds\nonumber \\  &-\sum _{\begin{array}{c} k\in \mathbb {Z}^{3,N}_0\\ k_3=0,\quad j\in \{1,2\} \end{array}}\int _0^t\langle \sigma _{k,j}^{\varepsilon }\cdot \nabla \overline{B^{H,\varepsilon }_{s}}-\overline{B^{\varepsilon }_{s}}\cdot \nabla \sigma _{k,j}^{H,\varepsilon },\nonumber \\  &-\nabla ^{\perp }_H(-\Delta )^{-1}\psi _h\rangle dW^{k,j}_s\nonumber \nonumber \\  &- \sum _{\begin{array}{c} k\in \mathbb {Z}^{3,N}_0\\ k_3\ne 0,\quad j\in \{1,2\} \end{array}}\int _0^t\langle \overline{\sigma _{k,j}^{\varepsilon }\cdot \nabla {{B}^{H,\varepsilon }_{s}}^{\prime }-{{B}^{\varepsilon }_{s}}^{\prime }\cdot \nabla \sigma _{k,j}^{H,\varepsilon }},\nonumber \\  &-\nabla ^{\perp }_H(-\Delta )^{-1}\psi _h\rangle dW^{k,j}_s. \end{aligned}$$

Recalling that, by (2.2) and Proposition 3.2, it holds

$$\begin{aligned} \overline{B^{\varepsilon }_{t}}&=\operatorname {curl}\overline{A^{\varepsilon }_{t}}\\ \sum _{\begin{array}{c} k\in \mathbb {Z}^{3,N}_0\\ k_3=0,\quad j\in \{1,2\} \end{array}}\sigma _{k,j}^{\varepsilon }\cdot \nabla \overline{B^{\varepsilon }_{t}}-\overline{B^{\varepsilon }_{t}}\cdot \nabla \sigma _{k,j}^{\varepsilon }&=\sum _{\begin{array}{c} k\in \mathbb {Z}^{3,N}_0\\ k_3=0,\quad j\in \{1,2\} \end{array}}\operatorname {curl}(\sigma _{k,j}^{\varepsilon }\cdot \nabla \overline{A^{\varepsilon }_{t}}+D\sigma _{k,j}^{\varepsilon }\overline{A^{\varepsilon }_{t}})\\ \sum _{\begin{array}{c} k\in \mathbb {Z}^{3,N}_0\\ k_3\ne 0,\quad j\in \{1,2\} \end{array}}\sigma _{k,j}^{\varepsilon }\cdot \nabla {{B}^{\varepsilon }_{t}}^{\prime }-{{B}^{\varepsilon }_{t}}^{\prime }\cdot \nabla \sigma _{k,j}^{\varepsilon }&=\sum _{\begin{array}{c} k\in \mathbb {Z}^{3,N}_0\\ k_3\ne 0,\quad j\in \{1,2\} \end{array}}\operatorname {curl}(\sigma _{k,j}^{\varepsilon }\cdot \nabla {{A}^{\varepsilon }_{t}}^{\prime }+D\sigma _{k,j}^{\varepsilon }{{A}^{\varepsilon }_{t}}^{\prime }), \end{aligned}$$

looking at the horizontal component, we have in particular

4.4 $$\begin{aligned} \overline{B^{H,\varepsilon }_{t}}&=-\nabla _{H}^{\perp }\overline{A^{3,\varepsilon }_{t}}\end{aligned}$$

4.5 $$\begin{aligned} \sum _{\begin{array}{c} k\in \mathbb {Z}^{3,N}_0\\ k_3=0,\quad j\in \{1,2\} \end{array}}\sigma _{k,j}^{\varepsilon }\cdot \nabla \overline{B^{H,\varepsilon }_{t}}-\overline{B^{\varepsilon }_{t}}\cdot \nabla \sigma _{k,j}^{H,\varepsilon }&=-\sum _{\begin{array}{c} k\in \mathbb {Z}^{3,N}_0\\ k_3=0,\quad j\in \{1,2\} \end{array}}\nabla _{H}^{\perp }(\sigma _{k,j}^{\varepsilon }\cdot \nabla \overline{A^{3,\varepsilon }_{t}})\end{aligned}$$

4.6 $$\begin{aligned} \sum _{\begin{array}{c} k\in \mathbb {Z}^{3,N}_0\\ k_3\ne 0,\quad j\in \{1,2\} \end{array}}\overline{\sigma _{k,j}^{\varepsilon }\cdot \nabla {{B}^{H,\varepsilon }_{t}}^{\prime }-{{B}^{\varepsilon }_{t}}^{\prime }\cdot \nabla \sigma _{k,j}^{H,\varepsilon }}&=-\sum _{\begin{array}{c} k\in \mathbb {Z}^{3,N}_0\\ k_3\ne 0,\quad j\in \{1,2\} \end{array}}\nabla _H^{\perp }\left( \overline{\sigma _{k,j}^{\varepsilon }\cdot \nabla {{A}^{3,\varepsilon }_{t}}^{\prime }+\partial _3\sigma _{k,j}^{\varepsilon }\cdot {{A}^{\varepsilon }_{t}}^{\prime }} \right) \nonumber \\  &=-\sum _{\begin{array}{c} k\in \mathbb {Z}^{3,N}_0\\ k_3\ne 0,\quad j\in \{1,2\} \end{array}}\nabla _H^{\perp }Q\left( \overline{\sigma _{k,j}^{\varepsilon }\cdot \nabla {{A}^{3,\varepsilon }_{t}}^{\prime }+\partial _3\sigma _{k,j}^{\varepsilon }\cdot {{A}^{\varepsilon }_{t}}^{\prime }} \right) . \end{aligned}$$

Now both $\sigma _{k,j}^{\varepsilon }\cdot \nabla \overline{A^{3,\varepsilon }_{t}}$ an $Q\left( \overline{\sigma _{k,j}^{\varepsilon }\cdot \nabla {{A}^{3,\varepsilon }_{t}}^{\prime }+\partial _3\sigma _{k,j}^{\varepsilon }\cdot {{A}^{\varepsilon }_{t}}^{\prime }} \right)$ are zero mean. Substituting (4.4), (4.5), (4.6) in (4.3) and integrating by parts we obtain

$$\begin{aligned} \langle \overline{A^{3,\varepsilon }_{t}},\psi _h\rangle&= \langle \overline{A^{3,\varepsilon }_{0}},\psi _h\rangle - \kappa _{\varepsilon }|h|^2 \int _0^t \langle \overline{A^{3,\varepsilon }_{s}},\psi _h\rangle \, ds\\&\quad -\sum _{\begin{array}{c} k\in \mathbb {Z}^{3,N}_0\\ k_3=0,\quad j\in \{1,2\} \end{array}}\int _0^t\langle \sigma _{k,j}^{\varepsilon }\cdot \nabla \overline{A^{3,\varepsilon }_{s}},\psi _h\rangle dW^{k,j}_s\nonumber \\  &\quad - \sum _{\begin{array}{c} k\in \mathbb {Z}^{3,N}_0\\ k_3\ne 0,\quad j\in \{1,2\} \end{array}}\int _0^t\left\langle Q\left[ \overline{\sigma _{k,j}^{\varepsilon }\cdot \nabla {{A}^{3,\varepsilon }_{s}}^{\prime }+\partial _3\sigma _{k,j}^{\varepsilon }\cdot {{A}^{\varepsilon }_{s}}^{\prime }}\right] ,\psi _h\right\rangle dW^{k,j}_s. \end{aligned}$$

Therefore, applying Itô formula to $e^{t\kappa _{\varepsilon }|h|^2}\langle \overline{A^{3,\varepsilon }_{t}},\psi _h\rangle$ we have

4.7 $$\begin{aligned} \langle \overline{A^{3,\varepsilon }_{t}},\psi _h\rangle&=e^{-t\kappa _{\varepsilon }|h|^2} \langle \overline{A^{3,\varepsilon }_{0}},\psi _h\rangle \nonumber \\  &\quad -\sum _{\begin{array}{c} k\in \mathbb {Z}^{3,N}_0\\ k_3=0,\quad j\in \{1,2\} \end{array}}\int _0^t e^{-(t-s)\kappa _{\varepsilon }|h|^2}\langle \sigma _{k,j}^{\varepsilon }\cdot \nabla \overline{A^{3,\varepsilon }_{s}},\psi _h\rangle dW^{k,j}_s\nonumber \\  &\quad - \sum _{\begin{array}{c} k\in \mathbb {Z}^{3,N}_0\\ k_3\ne 0,\quad j\in \{1,2\} \end{array}}\int _0^t e^{-(t-s)\kappa _{\varepsilon }|h|^2}\left\langle Q\left[ \overline{\sigma _{k,j}^{\varepsilon }\cdot \nabla {{A}^{3,\varepsilon }_{s}}^{\prime }+\partial _3\sigma _{k,j}^{\varepsilon }\cdot {{A}^{\varepsilon }_{s}}^{\prime }}\right] ,\right. \nonumber \\  &\left. \quad \psi _h\right\rangle dW^{k,j}_s\quad \mathbb {P}-a.s.\quad \forall t\in [0,T]. \end{aligned}$$

We can find $\Gamma \subseteq \Omega$ of full probability such that the above equality holds for all t∈[0,T] and all $h\in \mathbb {Z}^2_0$. But this is exactly (4.1) written in Fourier modes.

Let us consider for $h\in \mathbb {Z}^2_0$ $\phi =(0,0,\psi _h)^t,\ \psi _h=e^{ih\cdot x}$ as test function in for (2.28) obtaining, since all the functions appearing in the integrals are independent of $x_3$, that it holds

$$\begin{aligned} \langle \overline{B^{3,\varepsilon }_{t}},\psi _h\rangle&= \langle \overline{B^{3,\varepsilon }_{0}},\psi _h\rangle - \kappa _{\varepsilon }|h|^2\int _0^t \langle \overline{B^{3,\varepsilon }_{s}},\psi _h\rangle \, ds+\int _0^t \langle \operatorname {div}\left( \mathcal {R}_{\varepsilon ,\gamma }\nabla ^{\perp }\overline{A^{3,\varepsilon }_{s}}\right) ,\psi _h\rangle ds \\  &\quad -\sum _{\begin{array}{c} k\in \mathbb {Z}^{3,N}_0\\ k_3=0,\quad j\in \{1,2\} \end{array}}\int _0^t\langle \sigma _{k,j}^{\varepsilon }\cdot \nabla \overline{B^{3,\varepsilon }_{s}}-\overline{B^{\varepsilon }_{s}}\cdot \nabla \sigma _{k,j}^{3,\varepsilon },\psi _h\rangle dW^{k,j}_s\\  &\quad - \sum _{\begin{array}{c} k\in \mathbb {Z}^{3,N}_0\\ k_3\ne 0,\quad j\in \{1,2\} \end{array}}\int _0^t\langle \overline{\sigma _{k,j}^{\varepsilon }\cdot \nabla {{B}^{3,\varepsilon }_{s}}^{\prime }-{{B}^{\varepsilon }_{s}}^{\prime }\cdot \nabla \sigma _{k,j}^{3,\varepsilon }},\psi _h\rangle dW^{k,j}_s. \end{aligned}$$

Therefore, applying Itô formula to $e^{t\kappa _{\varepsilon }|h|^2}\langle \overline{B^{3,\varepsilon }_{t}},\psi _h\rangle$ we obtain

4.8 $$\begin{aligned} \langle \overline{B^{3,\varepsilon }_{t}},\psi _h\rangle&=e^{-t\kappa _{\varepsilon }|h|^2} \langle \overline{B^{3,\varepsilon }_{0}},\psi _h\rangle + \int _0^t e^{-(t-s)\kappa _{\varepsilon }|h|^2}\langle \operatorname {div}\left( \mathcal {R}_{\varepsilon ,\gamma }\nabla ^{\perp }\overline{A^{3,\varepsilon }_{s}}\right) ,\psi _h\rangle ds \nonumber \\  &\quad -\sum _{\begin{array}{c} k\in \mathbb {Z}^{3,N}_0\\ k_3=0,\quad j\in \{1,2\} \end{array}}\int _0^t e^{-(t-s)\kappa _{\varepsilon }|h|^2}\langle \sigma _{k,j}^{\varepsilon }\cdot \nabla \overline{B^{3,\varepsilon }_{s}}-\overline{B^{\varepsilon }_{s}}\cdot \nabla \sigma _{k,j}^{3,\varepsilon },\psi _h\rangle dW^{k,j}_s\nonumber \\  &\quad - \sum _{\begin{array}{c} k\in \mathbb {Z}^{3,N}_0\\ k_3\ne 0,\quad j\in \{1,2\} \end{array}}\int _0^t e^{-(t-s)\kappa _{\varepsilon }|h|^2}\langle \overline{\sigma _{k,j}^{\varepsilon }\cdot \nabla {{B}^{3,\varepsilon }_{s}}^{\prime }-{{B}^{\varepsilon }_{s}}^{\prime }\cdot \nabla \sigma _{k,j}^{3,\varepsilon }},\psi _h\rangle dW^{k,j}_s \nonumber \\  &\quad \mathbb {P}-a.s.\quad \forall t\in [0,T]. \end{aligned}$$

We can then find $\Gamma \subseteq \Omega$ of full probability such that the above equality holds for all t∈[0,T] and all $h\in \mathbb {Z}^2_0$. But this is exactly (4.2) written in Fourier modes. □

Secondly we need to study some properties of the stochastic convolutions $Z^{1,\varepsilon }_t,Z^{2,\varepsilon }_t$. Lemma 4.2 and Lemma 4.3 below are analogous to [6, Lemma 2.5] in our framework. In particular it is important to point out that [6, Assumption 2.4] is false in our case and we have to deal also with the stochastic stretching, which was neglected in previous results.

### Lemma 4.2

For each δ>0 and $N\in \mathbb {N}$ large enough such that Proposition 3.5 holds, then4.94.10The hidden constants in the inequality above depend only on $\eta ,\beta ,\gamma ,C_V,C_{1,H},C_{2,H},|\rho |,T$.

### Proof

By Burkholder-Davis-Gundy inequality and Lemma 2.1 we have for each t∈[0,T]

4.11 $$\begin{aligned} \mathbb {E} \left[ \left\| Z^{1,\varepsilon }_t\right\| _{\dot{H}^{-\delta }}^2\right]&\lesssim _{|\rho |} \sum _{\begin{array}{c} k\in \mathbb {Z}^{3,N}_0\\ k_3=0,\quad j\in \{1,2\} \end{array}}\mathbb {E} \left[ \int _0^t \left\| e^{(t-s)\kappa _{\varepsilon }\Delta }\left( \sigma _{k,j}^{\varepsilon }\cdot \nabla \overline{B^{3,\varepsilon }_{s}}-\overline{B^{\varepsilon }_{s}}\cdot \nabla \sigma _{k,j}^{3,\varepsilon }\right) \right\| _{\dot{H}^{-\delta }}^2 ds\right] \nonumber \\  &\quad +\sum _{\begin{array}{c} k\in \mathbb {Z}^{3,N}_0\\ k_3\ne 0,\quad j\in \{1,2\} \end{array}}\mathbb {E} \left[ \int _0^t \left\| e^{(t-s)\kappa _{\varepsilon }\Delta } \overline{\sigma _{k,j}^{\varepsilon }\cdot \nabla {{B}^{3,\varepsilon }_{s}}^{\prime }-{{B}^{\varepsilon }_{s}}^{\prime }\cdot \nabla \sigma _{k,j}^{3,\varepsilon }}\right\| _{\dot{H}^{-\delta }}^2 ds\right] \nonumber \\  &\lesssim _{\eta } \sum _{\begin{array}{c} k\in \mathbb {Z}^{3,N}_0\\ k_3=0,\quad j\in \{1,2\} \end{array}}\mathbb {E} \left[ \int _0^t \frac{1}{(t-s)^{1-\delta }}\left\| \sigma _{k,j}^{\varepsilon }\cdot \nabla \overline{B^{3,\varepsilon }_{s}}\right\| _{\dot{H}^{-1}}^2+\left\| \overline{B^{\varepsilon }_{s}}\cdot \nabla \sigma _{k,j}^{3,\varepsilon }\right\| ^2 ds\right] \nonumber \\  &\quad +\sum _{\begin{array}{c} k\in \mathbb {Z}^{3,N}_0\\ k_3\ne 0,\quad j\in \{1,2\} \end{array}}\mathbb {E} \left[ \int _0^t \frac{1}{(t-s)^{1-\delta }} \left\| \overline{\sigma _{k,j}^{\varepsilon }\cdot \nabla {{B}^{3,\varepsilon }_{s}}^{\prime }}\right\| _{\dot{H}^{-1}}^2+\left\| \overline{{{B}^{\varepsilon }_{s}}^{\prime }\cdot \nabla \sigma _{k,j}^{3,\varepsilon }}\right\| ^2ds\right] \nonumber \\  &=\frac{1}{\varepsilon }\sum _{\begin{array}{c} k\in \mathbb {Z}^{3,N}_0\\ k_3=0,\quad j\in \{1,2\} \end{array}}\mathbb {E} \left[ \int _0^t \frac{1}{(t-s)^{1-\delta }}\left\| \sigma _{k,j}^{\varepsilon }\cdot \nabla \overline{B^{3,\varepsilon }_{s}}\right\| _{\dot{H}^{-1}(\mathbb {T}^{3}_{\varepsilon })}^2+\left\| \overline{B^{\varepsilon }_{s}}\cdot \nabla \sigma _{k,j}^{3,\varepsilon }\right\| _{\varepsilon }^2 ds\right] \nonumber \\  &\quad +\frac{1}{\varepsilon }\sum _{\begin{array}{c} k\in \mathbb {Z}^{3,N}_0\\ k_3\ne 0,\quad j\in \{1,2\} \end{array}}\mathbb {E} \left[ \int _0^t \frac{1}{(t-s)^{1-\delta }}\left\| \overline{\sigma _{k,j}^{\varepsilon }\cdot \nabla {{B}^{3,\varepsilon }_{s}}^{\prime }}\right\| _{\dot{H}^{-1}(\mathbb {T}^{3}_{\varepsilon })}^2 +\left\| \overline{{{B}^{\varepsilon }_{s}}^{\prime }\cdot \nabla \sigma _{k,j}^{3,\varepsilon }}\right\| _{\varepsilon }^2ds\right] \nonumber \\  &= V^{1,\varepsilon }_t+V^{2,\varepsilon }_t+V^{3,\varepsilon }_t+V^{4,\varepsilon }_t. \end{aligned}$$

Now let us estimate $V^{1,\varepsilon }_t,\ V^{2,\varepsilon }_t,\ V^{3,\varepsilon }_t,\ V^{4,\varepsilon }_t$. First we have by Hölder’s inequality since the $\sigma _{k,j}^{\varepsilon }$ are divergence free

4.12 $$\begin{aligned} V^{1,\varepsilon }_t+V^{3,\varepsilon }_t&\le \frac{1}{\varepsilon }\mathbb {E} \left[ \sum _{\begin{array}{c} k\in \mathbb {Z}^{3,N}_0\\ k_3=0,\quad j=1 \end{array}}{\int _0^t \frac{1}{(t-s)^{1-\delta }} \left\| \sigma _{k,j}^{\varepsilon }\overline{B^{3,\varepsilon }_{s}}\right\| ^2_\varepsilon ds}+\sum _{\begin{array}{c} k\in \mathbb {Z}^{3,N}_0\\ k_3\ne 0,\quad j\in \{1,2\} \end{array}}{\int _0^t \frac{1}{(t-s)^{1-\delta } } \left\| \sigma _{k,j}^{\varepsilon }{{B}^{3,\varepsilon }_{s}}^{\prime }\right\| ^2_\varepsilon ds}\right] \nonumber \\  &\lesssim _{\beta ,C_{1,H},C_V} \frac{1}{\varepsilon }\left( {\int _0^t \frac{1}{(t-s)^{1-\delta }} \mathbb {E} \left[ \left\| \overline{B^{3,\varepsilon }_{s}}\right\| ^2_\varepsilon \right] ds}+\frac{1}{N^{\beta -2}}{\int _0^t \frac{1}{(t-s)^{1-\delta } }} \mathbb {E} \left[ \left\| {{B}^{3,\varepsilon }_{s}}^{\prime }\right\| ^2_\varepsilon \right] ds\right) \nonumber \\  &\lesssim _{\eta ,\beta ,\gamma ,C_V,C_{1,H},C_{2,H},|\rho |,T} \frac{1}{\varepsilon \delta }\left( {\left\| B_0^{3,\varepsilon }\right\| _{\varepsilon }^2}+{\left\| \overline{A^{3,\varepsilon }_{0}}\right\| _{\varepsilon }^2}+{\left\| {{A}^{\varepsilon }_{0}}^{\prime }\right\| _{\varepsilon }^2}\right) , \end{aligned}$$

where in the last step we exploit (3.39) and (3.41). For what concerns the other two terms, noting that if $k_3=0$ then $\sigma _{k,j}^{3,\varepsilon }\ne 0$ if and only if j=2, thanks to Hölder’s inequality and (3.38), (3.40) we get

4.13 $$\begin{aligned} V^{2,\varepsilon }_t+V^{3,\varepsilon }_t&\lesssim _{\beta , \gamma ,C_{2,H},C_V} \frac{1}{\varepsilon } \int _0^t \frac{1}{N^{\gamma -4}}\mathbb {E} \left[ \left\| \overline{B^{H,\varepsilon }_{s}}\right\| _{\varepsilon }^2\right] +\frac{1}{N^{\beta -4}} \mathbb {E} \left[ \left\| {{B}^{\varepsilon }_{s}}^{\prime }\right\| ^2_{\varepsilon }\right] ds\nonumber \\  &= \frac{1}{\varepsilon } \int _0^t \frac{1}{N^{\gamma -4}}\mathbb {E} \left[ \left\| \nabla \overline{A^{3,\varepsilon }_{s}}\right\| _{\varepsilon }^2\right] +\frac{1}{N^{\beta -4}} \mathbb {E} \left[ \left\| \nabla {{A}^{\varepsilon }_{s}}^{\prime }\right\| ^2_{\varepsilon }\right] ds\nonumber \\  &\lesssim _{\eta ,\beta ,\gamma ,C_V,C_{1,H},C_{2,H},|\rho |,T} \frac{1}{\varepsilon }\left( {\left\| B_0^{3,\varepsilon }\right\| _{\varepsilon }^2}+{\left\| \overline{A^{3,\varepsilon }_{0}}\right\| _{\varepsilon }^2}+{\left\| {{A}^{\varepsilon }_{0}}^{\prime }\right\| _{\varepsilon }^2}\right) . \end{aligned}$$

Combining (4.11), (4.12), (4.13) relation (4.9) follows. The proof of (4.10) is similar and we omit the details. □

### Lemma 4.3

For each δ>0 and $N\in \mathbb {N}$ large enough such that Proposition 3.5 holds, then4.144.15where the hidden constants depend only on $\eta ,\beta ,\gamma ,C_V,C_{1,H},C_{2,H},|\rho |,T$.

### Proof

By Burkholder-Davis-Gundy inequality and Lemma 2.1 we have for each t∈[0,T]

4.16 $$\begin{aligned} \mathbb {E} \left[ \left\| Z^{1,\varepsilon }_t\right\| _{\dot{H}^{-1-2\delta }}^2\right]&\lesssim _{|\rho |} \sum _{\begin{array}{c} k\in \mathbb {Z}^{3,N}_0\\ k_3=0,\quad j\in \{1,2\} \end{array}}\mathbb {E} \left[ \int _0^t \left\| e^{(t-s)\kappa _{\varepsilon }\Delta }\left( \sigma _{k,j}^{\varepsilon }\cdot \nabla \overline{B^{3,\varepsilon }_{s}}-\overline{B^{\varepsilon }_{s}}\cdot \nabla \sigma _{k,j}^{3,\varepsilon }\right) \right\| _{\dot{H}^{-1-2\delta }}^2 ds\right] \nonumber \\  &\quad +\sum _{\begin{array}{c} k\in \mathbb {Z}^{3,N}_0\\ k_3\ne 0,\quad j\in \{1,2\} \end{array}}\mathbb {E} \left[ \int _0^t \left\| e^{(t-s)\kappa _{\varepsilon }\Delta } \overline{\sigma _{k,j}^{\varepsilon }\cdot \nabla {{B}^{3,\varepsilon }_{s}}^{\prime }-{{B}^{\varepsilon }_{s}}^{\prime }\cdot \nabla \sigma _{k,j}^{3,\varepsilon }}\right\| _{\dot{H}^{-1-2\delta }}^2 ds\right] \nonumber \\  &\lesssim _{\eta } \sum _{\begin{array}{c} k\in \mathbb {Z}^{3,N}_0\\ k_3=0,\quad j\in \{1,2\} \end{array}}\mathbb {E} \left[ \int _0^t \frac{1}{(t-s)^{1-\delta }}\left\| \sigma _{k,j}^{\varepsilon }\cdot \nabla \overline{B^{3,\varepsilon }_{s}}\right\| _{\dot{H}^{-2-\delta }}^2+\left\| \overline{B^{\varepsilon }_{s}}\cdot \nabla \sigma _{k,j}^{3,\varepsilon }\right\| _{\dot{H}^{-1-2\delta }}^2 ds\right] \nonumber \\  &\quad +\sum _{\begin{array}{c} k\in \mathbb {Z}^{3,N}_0\\ k_3\ne 0,\quad j\in \{1,2\} \end{array}}\mathbb {E} \left[ \int _0^t {\left\| \overline{\sigma _{k,j}^{\varepsilon }\cdot \nabla {{B}^{3,\varepsilon }_{s}}^{\prime }}\right\| _{\dot{H}^{-1}}^2}+\left\| \overline{{{B}^{\varepsilon }_{s}}^{\prime }\cdot \nabla \sigma _{k,j}^{3,\varepsilon }}\right\| _{\dot{H}^{-1-2\delta }}^2ds\right] \nonumber \\  &=\sum _{\begin{array}{c} k\in \mathbb {Z}^{3,N}_0\\ k_3=0,\quad j\in \{1,2\} \end{array}}\mathbb {E} \left[ \int _0^t \frac{1}{(t-s)^{1-\delta }}\left\| \sigma _{k,j}^{\varepsilon }\cdot \nabla \overline{B^{3,\varepsilon }_{s}}\right\| _{\dot{H}^{-2-\delta }}^2+\left\| \overline{B^{\varepsilon }_{s}}\cdot \nabla \sigma _{k,j}^{3,\varepsilon }\right\| _{\dot{H}^{-1-2\delta }}^2 ds\right] \nonumber \\  &\quad +\sum _{\begin{array}{c} k\in \mathbb {Z}^{3,N}_0\\ k_3\ne 0,\quad j\in \{1,2\} \end{array}}\mathbb {E} \left[ \int _0^t \frac{1}{\varepsilon }\left\| \overline{\sigma _{k,j}^{\varepsilon }\cdot \nabla {{B}^{3,\varepsilon }_{s}}^{\prime }}\right\| _{\dot{H}^{-1}(\mathbb {T}^{3}_{\varepsilon })}^2 +\left\| \overline{{{B}^{\varepsilon }_{s}}^{\prime }\cdot \nabla \sigma _{k,j}^{3,\varepsilon }}\right\| _{\dot{H}^{-1-2\delta }}^2ds\right] \nonumber \\  &= Y^{1,\varepsilon }_t+Y^{2,\varepsilon }_t+Y^{3,\varepsilon }_t+Y^{4,\varepsilon }_t. \end{aligned}$$

The term $Y^{3,\varepsilon }_t$ can be simply treated as $V^{3,\varepsilon }_t$ in the proof of Lemma 4.2 obtaining

4.17 $$\begin{aligned} Y^{3,\varepsilon }_t \lesssim _{\eta ,\beta ,\gamma ,C_V,C_{1,H},C_{2,H},|\rho |,T} \frac{1}{\varepsilon N^{\beta -2}}\left( {\left\| B_0^{3,\varepsilon }\right\| _{\varepsilon }^2}+{\left\| \overline{A^{3,\varepsilon }_{0}}\right\| _{\varepsilon }^2}+{\left\| {{A}^{\varepsilon }_{0}}^{\prime }\right\| _{\varepsilon }^2}\right) . \end{aligned}$$

Let us consider now the others which are the more difficult ones. We will use in the following, even if not specified later, that all the vector fields considered are divergence free, moreover for $k_3=0$ we $\sigma _{k,j}^{H,\varepsilon }\ne 0$ if and only if j=1 and $\sigma _{k,j}^{3,\varepsilon }\ne 0$ if and only if j=2. Let us start considering $Y^{1,\varepsilon }_t$ obtaining, thanks to the definition of Sobolev norms in the two dimensional torus

4.18 $$\begin{aligned} Y^{1,\varepsilon }_t&\lesssim \sum _{\begin{array}{c} k\in \mathbb {Z}^{3,N}_0\\ k_3=0,\quad j=1 \end{array}}\mathbb {E} \left[ \int _0^t \frac{1}{(t-s)^{1-\delta }} \left\| \sigma _{k,j}^{H,\varepsilon } \overline{B^{3,\varepsilon }_{s}}\right\| _{\dot{H}^{-1-\delta }}^2 ds\right] \nonumber \\  &\lesssim \frac{1}{N^2}\sum _{k\in \mathbb {Z}^2_0}\mathbb {E} \left[ \int _0^t \frac{1}{(t-s)^{1-\delta }} \left\| e^{ik\cdot x} \overline{B^{3,\varepsilon }_{s}}\right\| _{\dot{H}^{-1-\delta }}^2 ds\right] \nonumber \\  &= \frac{1}{2\pi N^2}{\int _0^t \frac{1}{(t-s)^{1-\delta }}\sum _{k\in \mathbb {Z}^2_0}\sum _{j\in \mathbb {Z}^2_0}\frac{1}{|j|^{2+2\delta }} \mathbb {E} \left[ \langle \overline{B^{3,\varepsilon }_{s}}e^{ik\cdot x},e^{-ij\cdot x} \rangle ^2\right] ds}\nonumber \\  &=\frac{1}{2\pi N^2}{\int _0^t \frac{1}{(t-s)^{1-\delta }}\sum _{k\in \mathbb {Z}^2_0} \mathbb {E} \left[ \langle \overline{B^{3,\varepsilon }_{s}},e^{-ik\cdot x} \rangle ^2\right] \sum _{j\in \mathbb {Z}^2_0}\frac{1}{|j|^{2+2\delta }}ds}\nonumber \\  &\lesssim \frac{1}{\delta N^2} \int _0^t \frac{1}{(t-s)^{1-\delta }}\mathbb {E} \left[ \left\| \overline{B^{3,\varepsilon }_{s}}\right\| ^2\right] ds \nonumber \\  &\lesssim _{\eta ,\beta ,\gamma ,C_V,C_{1,H},C_{2,H},|\rho |,T} \frac{1}{\varepsilon \delta ^2 N^2} \left( {\left\| B_0^{3,\varepsilon }\right\| _{\varepsilon }^2}+{\left\| \overline{A^{3,\varepsilon }_{0}}\right\| _{\varepsilon }^2}+{\left\| {{A}^{\varepsilon }_{0}}^{\prime }\right\| _{\varepsilon }^2}\right) , \end{aligned}$$

where in the last step we exploited (3.39). Let us consider $Y^{2,\varepsilon }_t$ obtaining, thanks to (3.25)

4.19 $$\begin{aligned} Y^{2,\varepsilon }_t&\lesssim \sum _{\begin{array}{c} k\in \mathbb {Z}^{3,N}_0\\ k_3=0,\quad j=2 \end{array}}\int _0^t \mathbb {E} \left[ \left\| \sigma _{k,j}^{3,\varepsilon }\overline{B^{H,\varepsilon }_{s}}\right\| ^2\right] ds \nonumber \\  &\lesssim _{C_{2,H}} \sum _{\begin{array}{c} k\in \mathbb {Z}^2_0\\ N\le |k|\le 2N \end{array}} \frac{1}{|k|^{\gamma }} \int _0^T \mathbb {E} \left[ \left\| \overline{B^{H,\varepsilon }_{s}}\right\| ^2\right] ds\nonumber \\  &\lesssim _{\gamma } \frac{1}{N^{\gamma -2}} \int _0^T \mathbb {E} \left[ \left\| \nabla \overline{A^{3,\varepsilon }_{s}}\right\| ^2\right] ds\nonumber \\  &\lesssim _{\eta ,\beta ,\gamma ,C_V,C_{1,H},C_{2,H},|\rho |,T} \frac{1}{\varepsilon N^{\gamma -2}} \left( {\left\| B_0^{3,\varepsilon }\right\| _{\varepsilon }^2}+{\left\| \overline{A^{3,\varepsilon }_{0}}\right\| _{\varepsilon }^2}+{\left\| {{A}^{\varepsilon }_{0}}^{\prime }\right\| _{\varepsilon }^2}\right) , \end{aligned}$$

where in the last step we exploited (3.38). Lastly we treat $Y^{4,\varepsilon }_t$, obtaining thanks to (3.8) and the fact that $\operatorname {div}$ and $M^{\varepsilon }$ commute

4.20 $$\begin{aligned} Y^{4,\varepsilon }_t&\lesssim \sum _{\begin{array}{c} k\in \mathbb {Z}^{3,N}_0\\ k_3\ne 0,\quad j\in \{1,2\} \end{array}}\mathbb {E} \left[ \int _0^t \left\| \overline{{{B}^{\varepsilon }_{s}}^{\prime }\sigma _{k,j}^{3,\varepsilon }}\right\| ^2ds\right] \nonumber \\&= \frac{1}{\varepsilon }\sum _{\begin{array}{c} k\in \mathbb {Z}^{3,N}_0\\ k_3\ne 0,\quad j\in \{1,2\} \end{array}}\mathbb {E} \left[ \int _0^t \left\| \overline{{{B}^{\varepsilon }_{s}}^{\prime }\sigma _{k,j}^{3,\varepsilon }}\right\| _{\varepsilon }^2ds\right] \nonumber \\  &\le \frac{1}{\varepsilon }\sum _{\begin{array}{c} k\in \mathbb {Z}^{3,N}_0\\ k_3\ne 0,\quad j\in \{1,2\} \end{array}}\mathbb {E} \left[ \int _0^t \left\| {{{B}^{\varepsilon }_{s}}^{\prime }\sigma _{k,j}^{3,\varepsilon }}\right\| _{\varepsilon }^2ds\right] \nonumber \\  &\lesssim _{\beta , C_V} \frac{1}{\varepsilon N^{\beta -2}} \mathbb {E} \left[ \int _0^t \left\| {{B}^{\varepsilon }_{s}}^{\prime }\right\| _{\varepsilon }^2ds \right] \nonumber \\  &\lesssim _{\eta ,\beta ,\gamma ,C_V,C_{1,H},C_{2,H},|\rho |,T} \frac{1}{\varepsilon N^{\beta -2}} \left( {\left\| B_0^{3,\varepsilon }\right\| _{\varepsilon }^2}+{\left\| \overline{A^{3,\varepsilon }_{0}}\right\| _{\varepsilon }^2}+{\left\| {{A}^{\varepsilon }_{0}}^{\prime }\right\| _{\varepsilon }^2}\right) . \end{aligned}$$

Relation (4.14) follows combining (4.16), (4.17), (4.18), (4.19), (4.20). The proof of (4.15) is similar and we omit the details. □

Interpolating between Lemma 4.2 and Lemma 4.3 we obtain:

### Corollary 4.4

For each $\theta \in (0,1]$ and $\delta \in (0,\theta ]$ it holdsThe hidden constants in the inequality above depend only on $\eta ,\beta ,\gamma ,C_V,C_{1,H},C_{2,H},|\rho |,T$.

Moreover, we recall that by classical theory of evolution equations, see for example [18], or arguing as in Lemma 4.1, the unique weak solutions of (2.31), (2.30) can be written in mild form as

$$\begin{aligned} \overline{A_t^{3,\gamma }}=e^{t(\eta +\eta _T)\Delta }\overline{A^3_0}, \quad \overline{B_t^{3,\gamma }}=e^{t(\eta +\eta _T)\Delta }\overline{B^3_0}+\int _0^t e^{(t-s)(\eta +\eta _T)\Delta }\operatorname {div}\left( \mathcal {R}_{\gamma }\nabla ^{\perp }\overline{A^3_s}\right) ds. \end{aligned}$$

Now we introduce some intermediate functions between $(\overline{B^{3,\varepsilon }_{t}},\overline{A^{3,\varepsilon }_{t}})$ and $(\overline{B_t^3},\overline{A_t^3})$. Let $\left( \widehat{B_t^{3,\varepsilon }},\widehat{A_t^{3,\varepsilon }}\right)$ the unique weak solutions of the linear systems

4.21 $$\begin{aligned}&{\left\{ \begin{array}{ll} \partial _t \widehat{A^{3,\varepsilon }_t}& =(\eta +\eta ^{\varepsilon }_T)\Delta \widehat{A^{3,\varepsilon }_t}\quad x\in \mathbb {T}^2,\ t\in (0,T)\\ \widehat{A^{3,\varepsilon }_t}|_{t=0}& =\overline{A^{3,\varepsilon }_{0}}, \end{array}\right. }\end{aligned}$$

4.22 $$\begin{aligned}&{\left\{ \begin{array}{ll} \partial _t \widehat{B^{3,\varepsilon }_t}& =(\eta +\eta ^{\varepsilon }_T)\Delta \widehat{B^{3,\gamma }_t}+\operatorname {div}\left( \mathcal {R}_{\varepsilon ,\gamma }\nabla ^{\perp }\widehat{A^{3,\varepsilon }_t}\right) \quad x\in \mathbb {T}^2,\ t\in (0,T)\\ \widehat{B^{3,\varepsilon }_t}|_{t=0}& =\overline{B^{3,\varepsilon }_{0}}. \end{array}\right. } \end{aligned}$$

Again, by classical theory of evolution equations, see for example [18], or arguing as in Lemma 4.1, the unique weak solutions of (4.21), (4.22) can be written in mild form as

$$\begin{aligned}&\widehat{A_t^{3,\varepsilon }}=e^{t(\eta +\eta ^{\varepsilon }_T)\Delta }\overline{A^{3,\varepsilon }_{0}}, \quad \\  &\widehat{B_t^{3,\varepsilon }}=e^{t(\eta +\eta ^{\varepsilon }_T)\Delta }\overline{B^{3,\varepsilon }_{0}}+\int _0^t e^{(t-s)(\eta +\eta _T^{\varepsilon })\Delta } \operatorname {div}\left( \mathcal {R}_{\varepsilon ,\gamma }\nabla ^{\perp }\widehat{A^{3,\varepsilon }_s}\right) ds. \end{aligned}$$

Now we prove that $(\widehat{B^{3,\varepsilon }_t},\widehat{A^{3,\varepsilon }_t})$ and $(\overline{B^{3,\gamma }_t},\overline{A^{3,\gamma }_t})$ are close.

### Lemma 4.5

Under the same assumptions of Theorem 2.15, for each $\theta _1\in (0,1],\ \theta _2\in (0,\theta _1)$ we have

4.23 $$\begin{aligned}&\operatorname {sup}_{t\in [0,T]}\left\| \widehat{A^{3,\varepsilon }_t}-\overline{A^{3,\gamma }_t}\right\| _{\dot{H}^{-\theta _2}}\lesssim _{\eta ,T}|\eta ^{\varepsilon }_T-\eta _T|^{\theta _2/2}+\left\| \overline{v^3_0}-\overline{A^{3,\varepsilon }_{0}}\right\| _{\dot{H}^{-\theta _2}}, \end{aligned}$$

4.24 $$\begin{aligned}&\operatorname {sup}_{t\in [0,T]}\left\| \widehat{B^{3,\varepsilon }_t}-\overline{B^{3,\gamma }_t}\right\| _{\dot{H}^{-\theta _1}}\nonumber \\  &\quad \lesssim _{\eta ,T} {\left\{ \begin{array}{ll} \frac{|\eta ^{\varepsilon }_T-\eta _T|^{\theta _2/2}+\left\| \overline{A^3_0}-\overline{A^{3,\varepsilon }_{0}}\right\| _{\dot{H}^{-\theta _2}}}{\theta _1-\theta _2}+\left\| \overline{B^3_0}-\overline{B^{3,\varepsilon }_{0}}\right\| _{\dot{H}^{-\theta _1}}+\frac{1}{\theta _1 N}\\ \text {if }\gamma =4,\\ \frac{|\eta ^{\varepsilon }_T-\eta _T|^{\theta _2/2}+\left\| \overline{A^3_0}-\overline{A^{3,\varepsilon }_{0}}\right\| _{\dot{H}^{-\theta _2}}}{\theta _1-\theta _2}+\left\| \overline{B^3_0}-\overline{B^{3,\varepsilon }_{0}}\right\| _{\dot{H}^{-\theta _1}}+\frac{1}{\theta _1 N^{\gamma /2-2}} \\ \text {if }\gamma >4. \end{array}\right. } \end{aligned}$$

### Proof

First let us observe that by assumptions

$$\begin{aligned} \overline{B^{3,\varepsilon }_{0}}\rightharpoonup \overline{B^3_0} \text { in }\dot{L}^2(\mathbb {T}^2),\quad \overline{A^{3,\varepsilon }_{0}}\rightharpoonup \overline{A^3_0} \text { in }\dot{L}^2(\mathbb {T}^2) \end{aligned}$$

we have in particular that the families $\left\{ \left\| \overline{B^{3,\varepsilon }_{0}}\right\| ^2\right\} _{\varepsilon \in (0,1)},\ \left\{ \left\| \overline{A^{3,\varepsilon }_{0}}\right\| ^2\right\} _{\varepsilon \in (0,1)}$ are bounded. Therefore

4.25 $$\begin{aligned} \operatorname {sup}_{\varepsilon \in (0,1)}\frac{{\left\| \overline{B^{3,\varepsilon }_{0}}\right\| _{\varepsilon }^2}}{\varepsilon }=\operatorname {sup}_{\varepsilon \in (0,1)}{\left\| \overline{B^{3,\varepsilon }_{0}}\right\| }^2<+\infty ,\,\,\overline{B^{3,\varepsilon }_{0}}\rightarrow \overline{B^3_0} \text { in }\dot{H}^{-\theta _2}(\mathbb {T}^2)\quad \forall \theta _2>0,\nonumber \\ \operatorname {sup}_{\varepsilon \in (0,1)}\frac{{\left\| \overline{A^{3,\varepsilon }_{0}}\right\| _{\varepsilon }^2}}{\varepsilon }=\operatorname {sup}_{\varepsilon \in (0,1)}{\left\| \overline{A^{3,\varepsilon }_{0}}\right\| }^2<+\infty ,\,\,\overline{A^{3,\varepsilon }_{0}}\rightarrow \overline{A^3_0} \text { in }\dot{H}^{-\theta _2}(\mathbb {T}^2)\quad \forall \theta _2>0. \end{aligned}$$

Moreover, since by Lemma 2.10$\mathcal {R}_{\varepsilon ,\gamma }\rightarrow \mathcal {R}_{\varepsilon },$ we have also

4.26 $$\begin{aligned} \operatorname {sup}_{\varepsilon \in (0,1)}\left\| \mathcal {R}_{\varepsilon ,\gamma }\right\| _{HS}<+\infty . \end{aligned}$$

Lastly, by simple energy estimates on (4.21) we have for each $\varepsilon \in (0,1)$

4.27 $$\begin{aligned} \operatorname {sup}_{t\in [0,T]}\left\| \widehat{A^{3,\varepsilon }_t}\right\| ^2+\int _0^T \left\| \nabla \widehat{A^{3,\varepsilon }_s}\right\| ^2 ds\lesssim _{\eta } \operatorname {sup}_{\varepsilon \in (0,1)}{\left\| \overline{A^{3,\varepsilon }_{0}}\right\| }^2<+\infty . \end{aligned}$$

The convergence of $\widehat{A^{\varepsilon ,3}_t}$ to $\overline{A^{3,\gamma }_t}$ then follows by triangle inequality, Lemma 2.1 and (4.25). Indeed for each t∈[0,T] it holds

$$\begin{aligned} \left\| \widehat{A^{3,\varepsilon }_t}-\overline{A^{3,\gamma }_t}\right\| _{\dot{H}^{-\theta _2}}&\le \left\| \left( e^{t(\eta +\eta _T^{\varepsilon })\Delta }-e^{t(\eta +\eta _T)\Delta }\right) \overline{A^3_0}\right\| _{\dot{H}^{-\theta _2}}\\  &\quad +\left\| e^{t(\eta +\eta ^{\varepsilon }_T)\Delta }\left( \overline{A^{3,\varepsilon }_{0}}-\overline{A^3_0}\right) \right\| _{\dot{H}^{-\theta _2}}\\  &\lesssim _{\eta ,T}|\eta ^{\varepsilon }_T-\eta _T|^{\theta _2/2}\left\| \overline{A^3_0}\right\| +\left\| \overline{A^3_0}-\overline{A^{3,\varepsilon }_{0}}\right\| _{\dot{H}^{-\theta _2}}\\  &\lesssim |\eta ^{\varepsilon }_T-\eta _T|^{\theta _2/2}+\left\| \overline{A^3_0}-\overline{A^{3,\varepsilon }_{0}}\right\| _{\dot{H}^{-\theta _2}}. \end{aligned}$$

Thanks to (4.23) we can obtain also (4.24). Indeed thanks to triangle inequality for each t∈[0,T] we have

$$\begin{aligned} \left\| \widehat{B^{3,\varepsilon }_t}-\overline{B^{3,\gamma }_t}\right\| _{\dot{H}^{-\theta _1}}&\le \left( \left\| \left( e^{t(\eta +\eta _T^{\varepsilon })\Delta }-e^{t(\eta +\eta _T)\Delta }\right) \overline{B^3_0}\right\| _{\dot{H}^{-\theta _1}} \right. \\  &\quad \left. +\left\| e^{t(\eta +\eta ^{\varepsilon }_T)\Delta }\left( \overline{B^{3,\varepsilon }_{0}}-\overline{B^3_0}\right) \right\| _{\dot{H}^{-\theta _1}}\right) \\  &\quad +\left\| \int _0^t \left( e^{(t-s)(\eta +\eta _T^{\varepsilon })\Delta }-e^{(t-s)(\eta +\eta _T)\Delta }\right) \operatorname {div}\left( \mathcal {R}_{\varepsilon ,\gamma }\nabla ^{\perp }\widehat{A^{3,\varepsilon }_s}\right) ds\right\| _{\dot{H}^{-\theta _1}} \\  &\quad + \left\| \int _0^t e^{(t-s)(\eta +\eta _T)\Delta } \operatorname {div}\left( \left( \mathcal {R}_{\varepsilon ,\gamma }-\mathcal {R}_{\gamma }\right) \nabla ^{\perp }\widehat{A^{3,\varepsilon }_s}\right) ds\right\| _{\dot{H}^{-\theta _1}}\\  &\quad + \left\| \int _0^t e^{(t-s)(\eta +\eta _T)\Delta } \operatorname {div}\left( \mathcal {R}_{\gamma }\left( \nabla ^{\perp }\widehat{A^{3,\varepsilon }_s}-\nabla ^{\perp }\overline{A^{3,\gamma }_s}\right) \right) ds\right\| _{\dot{H}^{-\theta _1}}\\  &=I_1+I_2+I_3+I_4. \end{aligned}$$

$I_1$ can be treated as above obtaining:

4.28 $$\begin{aligned} I_1\lesssim _{\eta ,T}|\eta ^{\varepsilon }_T-\eta _T|^{\theta _1/2}+\left\| \overline{B^3_0}-\overline{B^{3,\varepsilon }_{0}}\right\| _{\dot{H}^{-\theta _1}}. \end{aligned}$$

Thanks to Lemma 2.1, (4.26) and (4.27) it holds

4.29 $$\begin{aligned} I_2&\lesssim |\eta ^{\varepsilon }_T-\eta _T|^{\theta _1/2}\int _0^t|t-s|^{\theta _1/2}\left\| e^{(t-s)\eta \Delta }\operatorname {div}\left( \mathcal {R}_{\varepsilon ,\gamma }\nabla ^{\perp }\widehat{A^{3,\varepsilon }_s}\right) \right\| ds\nonumber \\  &\lesssim _{\eta } |\eta ^{\varepsilon }_T-\eta _T|^{\theta _1/2}\int _0^t \frac{1}{(t-s)^{1-\theta _1/2}}\left\| \widehat{A^{3,\varepsilon }_s}\right\| ds\nonumber \\  &\lesssim _{T} \frac{1}{\theta _1} |\eta ^{\varepsilon }_T-\eta _T|^{\theta _1/2}. \end{aligned}$$

Due to Lemma 2.1 and (4.27) we can treat $I_3$ obtaining:

4.30 $$\begin{aligned} I_3&\lesssim _{\eta ,\theta _1} \int _0^T \frac{1}{(t-s)^{1-\theta _1/2}} \left\| \operatorname {div}\left( \left( \mathcal {R}_{\varepsilon ,\gamma }-\mathcal {R}_{\gamma }\right) \nabla ^{\perp }\widehat{A^{3,\varepsilon }_s}\right) \right\| _{\dot{H}^{-2}} ds \nonumber \\  &\lesssim \left\| \mathcal {R}_{\varepsilon ,\gamma }-\mathcal {R}_{\gamma }\right\| _{HS}\int _0^T \frac{1}{(t-s)^{1-\theta _1/2}} \left\| \widehat{A^{3,\varepsilon }_s}\right\| ds \nonumber \\  &\lesssim _{T}\frac{1}{\theta _1}\left\| \mathcal {R}_{\varepsilon ,\gamma }-\mathcal {R}_{\gamma }\right\| _{HS}. \end{aligned}$$

In particular, combining (4.30) and Lemma 2.10 we have that

4.31 $$\begin{aligned} I_3\lesssim _{\eta ,T}{\left\{ \begin{array}{ll} \frac{1}{\theta _1 N} &  \text {if } \gamma =4,\\ \frac{1}{\theta _1 N^{\gamma /2-2}} &  \text {if } \gamma >4. \end{array}\right. } \end{aligned}$$

Lastly we treat $I_4$ combining Lemma 2.1 and (4.23):

4.32 $$\begin{aligned} I_4&\lesssim _{\eta } \int _0^t \frac{1}{(t-s)^{1+\theta _2/2-\theta _1/2}} \left\| \widehat{A^{3,\varepsilon }_s}-\overline{A^{3,\gamma }_s}\right\| _{\dot{H}^{-\theta _2}}ds\nonumber \\  &\lesssim _{T} \frac{1}{\theta _1-\theta _2}\operatorname {sup}_{t\in [0,T]} \left\| \widehat{A^{3,\varepsilon }_t}-\overline{A^{3,\gamma }_t}\right\| _{\dot{H}^{-\theta _2}}\nonumber \\  &\lesssim _{\eta ,T} \frac{|\eta ^{\varepsilon }_T-\eta _T|^{\theta _2/2}+\left\| \overline{A^3_0}-\overline{A^{3,\varepsilon }_{0}}\right\| _{\dot{H}^{-\theta _2}}}{\theta _1-\theta _2} . \end{aligned}$$

Combining (4.28),(4.29),(4.31),(4.32) we get the claim. □

Secondly we provide a quantitative result on the closeness of $(\widehat{B^{3,\varepsilon }_t},\widehat{A^{3,\varepsilon }_t})$ and $(\overline{B^{3,\varepsilon }_{t}},\overline{A^{3,\varepsilon }_{t}}).$

### Lemma 4.6

Under the same assumptions of Theorem 2.15, there exists $N_0$ large enough such that for each $N\ge N_0$, for each $\theta _1\in (0,1],\ \theta _2\in (0,\theta _1),\ \delta \in (0,\theta _2)$ we have4.334.34where the hidden constants depend only on $\eta ,\beta ,\gamma ,C_V,C_{1,H},C_{2,H},|\rho |, T$.

### Proof

From Lemma 4.1, we already know that $\overline{B^{3,\varepsilon }_{t}}$ and $\overline{A^{3,\varepsilon }_{t}}$ can be rewritten in mild form as

$$\begin{aligned}&\overline{B^{3,\varepsilon }_{t}}=e^{t(\eta +\eta _T^{\varepsilon })\Delta }\overline{B^{3,\varepsilon }_{0}}-Z_{t}^{1,\varepsilon }+\int _0^t e^{(t-s)(\eta +\eta ^{\varepsilon }_T)\Delta }\operatorname {div}\left( \mathcal {R}_{\varepsilon ,\gamma }\nabla ^{\perp }\overline{A^{3,\varepsilon }_{s}}\right) ds,\quad \\  &\overline{A^{3,\varepsilon }_{t}}=e^{t(\eta +\eta _T^{\varepsilon })\Delta }\overline{A^{3,\varepsilon }_{0}}-Z_{t}^{2,\varepsilon }. \end{aligned}$$

Therefore denoting by

$$\begin{aligned} D^{\varepsilon }_t=\overline{B^{3,\varepsilon }_{t}}-\widehat{B^{3,\varepsilon }_t},\quad d^{\varepsilon }_t=\overline{A^{3,\varepsilon }_{t}}-\widehat{A^{3,\varepsilon }_t}, \end{aligned}$$

we have

$$\begin{aligned} D^{\varepsilon }_t=-Z_{t}^{1,\varepsilon }+\int _0^t e^{(t-s)(\eta +\eta ^{\varepsilon }_T)\Delta }\operatorname {div}\left( \mathcal {R}_{\varepsilon ,\gamma }\nabla ^{\perp }d^{\varepsilon }_s\right) ds,\quad d^{\varepsilon }_s=-Z_{t}^{2,\varepsilon }. \end{aligned}$$

Due to Corollary 4.4 we immediately obtain (4.33). Now we move to the analysis of $D^{\varepsilon }_t$. $Z^{1,\varepsilon }_t$ can be treated easily by Corollary 4.4 obtaining

4.35 $$\begin{aligned} \operatorname {sup}_{t\in [0,T]}\mathbb {E} \left[ \left\| Z^{1,\varepsilon }_t\right\| ^2_{\dot{H}^{-\theta _1}}\right]&\lesssim _{\eta ,\beta ,\gamma ,C_V,C_{1,H},C_{2,H},|\rho |, T} \frac{1}{\delta ^{\frac{1+\theta _1}{1+\delta }}N^{\frac{2(\theta _1-\delta )}{1+\delta }}}. \end{aligned}$$

To treat the deterministic convolution, we first argue by interpolation obtaining thanks to Lemma 2.1, (4.26)

4.36 $$\begin{aligned}&\left\| \int _0^t e^{(t-s)(\eta +\eta ^{\varepsilon }_T)\Delta }\operatorname {div}\left( \mathcal {R}_{\varepsilon ,\gamma }\nabla ^{\perp }d^{\varepsilon }_s\right) ds\right\| ^2_{\dot{H}^{-\theta _1}}\nonumber \\  &\quad \le \left\| \int _0^t e^{(t-s)(\eta +\eta ^{\varepsilon }_T)\Delta }\operatorname {div}\left( \mathcal {R}_{\varepsilon ,\gamma }\nabla ^{\perp }d^{\varepsilon }_s\right) ds\right\| ^{\theta _1-\theta _2}_{\dot{H}^{-2-\theta _2}} \nonumber \\  &\qquad \times \left\| \int _0^t e^{(t-s)(\eta +\eta ^{\varepsilon }_T)\Delta }\operatorname {div}\left( \mathcal {R}_{\varepsilon ,\gamma }\nabla ^{\perp }d^{\varepsilon }_s\right) ds\right\| ^{2-\theta _1+\theta _2}_{\dot{H}^{-\theta _2}}\nonumber \\  &\quad \lesssim _{\eta } \left( \int _0^t \left\| d^{\varepsilon }_s\right\| _{\dot{H}^{-\theta _2}}ds \right) ^{\theta _1-\theta _2}\left( \int _0^t \frac{1}{(t-s)^{1-\theta _2/2}} \left\| \widehat{A^{3,\varepsilon }_s}-\overline{A^{3,\varepsilon }_{s}}\right\| ds\right) ^{2-\theta _1+\theta _2}\nonumber \\  &\quad \lesssim _{T} \frac{1}{\theta _2^{2-\theta _1+\theta _2}}\left( \int _0^t \left\| d^{\varepsilon }_s\right\| _{\dot{H}^{-\theta _2}}^2ds \right) ^{\frac{\theta _1-\theta _2}{2}}\operatorname {sup}_{t\in [0,T]}\left( \left\| \overline{A^{3,\varepsilon }_{t}}\right\| +\left\| \widehat{A^{3,\varepsilon }_t}\right\| \right) ^{2-\theta _1+\theta _2}. \end{aligned}$$

Combining (4.36) and (4.33), (), (4.27) we obtain an estimate of the deterministic convolution. Indeed, by Hölder’s inequality, for each t∈[0,T] it holds:

4.37 $$\begin{aligned}&\mathbb {E} \left[ \left\| \int _0^t e^{(t-s)(\eta +\eta ^{\varepsilon }_T)\Delta }\operatorname {div}\left( \mathcal {R}_{\varepsilon ,\gamma }\nabla ^{\perp }d^{\varepsilon }_s\right) ds\right\| ^2_{\dot{H}^{-\theta _1}}\right] \nonumber \\  &\quad \lesssim _{\eta ,T}\frac{1}{\theta _2^{2-\theta _1+\theta _2}} \mathbb {E} \left[ \left( \int _0^T \left\| d^{\varepsilon }_s\right\| _{\dot{H}^{-\theta _2}}^2ds \right) ^{\frac{\theta _1-\theta _2}{2}}\operatorname {sup}_{t\in [0,T]}\left( \left\| \overline{A^{3,\varepsilon }_{t}}\right\| +\left\| \widehat{A^{3,\varepsilon }_t}\right\| \right) ^{2-\theta _1+\theta _2}\right] \nonumber \\  &\quad \le \frac{1}{\theta _2^{2-\theta _1+\theta _2}} \mathbb {E} \left[ \int _0^T \left\| d^{\varepsilon }_s\right\| _{\dot{H}^{-\theta _2}}^2ds \right] ^{\frac{\theta _1-\theta _2}{2}}\mathbb {E} \left[ \operatorname {sup}_{t\in [0,T]}\left( \left\| \overline{A^{3,\varepsilon }_{t}}\right\| ^2+\left\| \widehat{A^{3,\varepsilon }_t}\right\| ^2\right) \right] ^{\frac{2-\theta _1+\theta _2}{2}}\nonumber \\  &\quad \lesssim _{\eta ,\beta ,\gamma ,C_V,C_{1,H},C_{2,H},|\rho |, T} \frac{1}{\theta _2^{2-\theta _1+\theta _2}\delta ^{\frac{(\theta _1-\theta _2)(1+\theta _2)}{2(1+\delta )}}N^{\frac{(\theta _2-\delta )(\theta _1-\theta _2)}{(1+\delta )}}}. \end{aligned}$$

Combining (4.35) and (4.37) the claim follows. □

Now we are ready to prove Theorem 2.15.

### Proof of Theorem 2.15

Recalling that, due to the properties of Riemann sums,

$$\begin{aligned} |\eta ^{\varepsilon }_T-\eta _T|\lesssim \frac{1}{N}, \end{aligned}$$

then the claim follows immediately by triangle inequality combining Subsection 4.5 and Subsection 4.6. □

## Data Availability

No datasets were generated or analysed during the current study.

## Proof of Lemma 2.12

We start showing that

$$\begin{aligned} \sum _{\begin{array}{c} k\in \mathbb {Z}^{3,N}_0\\ j\in \{1,2\} \end{array}}\mathcal {L}_{\sigma _{k,j}^{\varepsilon }}\mathcal {L}_{\sigma _{-k,j}^{\varepsilon }} F&=\Lambda ^{\varepsilon }F. \end{aligned}$$

First let us observe that it holds

$$\begin{aligned} \sum _{\begin{array}{c} k\in \mathbb {Z}^{3,N}_0\\ j\in \{1,2\} \end{array}}\mathcal {L}_{\sigma _{k,j}^{\varepsilon }}\mathcal {L}_{\sigma _{-k,j}^{\varepsilon }} F&= \sum _{\begin{array}{c} k\in \mathbb {Z}^{3,N}_0\\ j\in \{1,2\} \end{array}}\operatorname {div}(\sigma _{k,j}^{\varepsilon }\otimes \sigma _{-k,j}^{\varepsilon }\nabla F)-R\\  &=\operatorname {div}\left( Q^{\varepsilon }_0(0) \nabla F\right) -R\\  &= \Lambda ^{\varepsilon }F-R, \end{aligned}$$

where we denoted by

$$\begin{aligned} R=\sum _{\begin{array}{c} k\in \mathbb {Z}^{3,N}_0\\ j\in \{1,2\} \end{array}}\sigma _{k,j}^{\varepsilon }\cdot \nabla \left( F\cdot \nabla \sigma _{-k,j}^{\varepsilon }\right) +\left( \sigma _{-k,j}^{\varepsilon }\cdot \nabla F\right) \cdot \nabla \sigma _{k,j}^{\varepsilon }-(F\cdot \nabla \sigma _{-k,j}^{\varepsilon })\cdot \nabla \sigma _{k,j}^{\varepsilon }. \end{aligned}$$

Therefore, it is enough to show that

A.1 $$\begin{aligned} R=0. \end{aligned}$$

First we observe that

$$\begin{aligned} \sigma _{k,j}^{\varepsilon }\cdot \nabla \left( F\cdot \nabla \sigma _{-k,j}^{\varepsilon }\right) =(\sigma _{k,j}^{\varepsilon }\cdot \nabla F)\cdot \nabla \sigma _{-k,j}^{\varepsilon }+\sigma _{k,j}^{\varepsilon }\cdot \nabla ^2 \sigma _{-k,j}^{\varepsilon }F. \end{aligned}$$

Therefore

A.2 $$\begin{aligned} R&=\left( \sum _{\begin{array}{c} k\in \mathbb {Z}^{3,N}_0\\ j\in \{1,2\} \end{array}}(\sigma _{k,j}^{\varepsilon }\cdot \nabla F)\cdot \nabla \sigma _{-k,j}^{\varepsilon }+\left( \sigma _{-k,j}^{\varepsilon }\cdot \nabla F\right) \cdot \nabla \sigma _{k,j}^{\varepsilon }\right) \nonumber \\  &\quad +\left( \sum _{\begin{array}{c} k\in \mathbb {Z}^{3,N}_0\\ j\in \{1,2\} \end{array}}\sigma _{k,j}^{\varepsilon }\cdot \nabla ^2 \sigma _{-k,j}^{\varepsilon }F-(F\cdot \nabla \sigma _{-k,j}^{\varepsilon })\cdot \nabla \sigma _{k,j}^{\varepsilon }\right) \nonumber \\  &= R_1+R_2. \end{aligned}$$

$R_1$ is easily 0 due to (2.17). In order to show that $R_2=0$, we observe that, recalling the definition of $\sigma _{k,j}^{\varepsilon }$ and expanding the sum, it holds for each $k\in \mathbb {Z}^{3,N}_0,\ j\in \{1,2\}$

A.3 $$\begin{aligned} \sigma _{k,j}^{\varepsilon }\cdot \nabla ^2 \sigma _{-k,j}^{\varepsilon }F-(F\cdot \nabla \sigma _{-k,j}^{\varepsilon })\cdot \nabla \sigma _{k,j}^{\varepsilon }&= -2\sum _{l,m\in \{1,2,3\}}\left\lvert \theta _{k,j}^{\varepsilon }\right\rvert ^2 a_{k,j} k_l k_m(a_{k,j})_l F_m \nonumber \\  &=2\sigma _{k,j}^{\varepsilon }\cdot \nabla ^2 \sigma _{-k,j}^{\varepsilon }F\nonumber \\  &=2\sum _{m\in \{1,2,3\}}\sigma _{k,j}^{\varepsilon }\cdot \nabla \partial _m\sigma _{-k,j}^{\varepsilon }F_m. \end{aligned}$$

Let us show that for each $m\in \{1,2,3\}$ it holds $$\sum _{\begin{array}{c} k\in \mathbb {Z}^{3,N}_0\\ j\in \{1,2\} \end{array}}\sigma _{k,j}^{\varepsilon }\cdot \nabla \partial _m\sigma _{-k,j}^{\varepsilon }=0$$. Due to the fact that $\sigma _{k,j}^{\varepsilon }$ are divergence free, we have

A.4 $$\begin{aligned} \sum _{\begin{array}{c} k\in \mathbb {Z}^{3,N}_0\\ j\in \{1,2\} \end{array}}\sigma _{k,j}^{\varepsilon }\cdot \nabla \partial _m\sigma _{-k,j}^{\varepsilon }&=\operatorname {div}\left( \sum _{\begin{array}{c} k\in \mathbb {Z}^{3,N}_0\\ j\in \{1,2\} \end{array}}\sigma _{k,j}^{\varepsilon }\otimes \partial _m \sigma _{-k,j}^{\varepsilon }\right) \nonumber \\  &= i\operatorname {div}\left( \sum _{\begin{array}{c} k\in \mathbb {Z}^{3,N}_0\\ j\in \{1,2\} \end{array}}\left\lvert \theta _{k,j}^{\varepsilon }\right\rvert ^2 k_m a_{k,j}\otimes a_{k,j}\right) =0. \end{aligned}$$

Our claim (A.1) then follows combining (A.2), (A.3), (A.4).

Now let us prove that

$$\begin{aligned} \rho \sum _{\begin{array}{c} k\in \mathbb {Z}^{3,N}_0\\ k_3=0 \end{array}}\left( \mathcal {L}_{\sigma _{k,1}^{\varepsilon }}\mathcal {L}_{\sigma _{-k,2}^{\varepsilon }}+\mathcal {L}_{\sigma _{k,2}^{\varepsilon }}\mathcal {L}_{\sigma _{-k,1}^{\varepsilon }}\right) F&=\Lambda _{\rho }^{\varepsilon }F. \end{aligned}$$

First let us observe that it holds

$$\begin{aligned}&\sum _{\begin{array}{c} k\in \mathbb {Z}^{3,N}_0\\ k_3=0 \end{array}}\left( \mathcal {L}_{\sigma _{k,1}^{\varepsilon }}\mathcal {L}_{\sigma _{-k,2}^{\varepsilon }}+\mathcal {L}_{\sigma _{k,2}^{\varepsilon }}\mathcal {L}_{\sigma _{-k,1}^{\varepsilon }}\right) F \\  &\quad =\sum _{\begin{array}{c} k\in \mathbb {Z}^{3,N}_0\\ k_3=0 \end{array}}\operatorname {div}\left( (\sigma _{k,1}^{\varepsilon }\otimes \sigma _{-k,2}^{\varepsilon }+\sigma _{k,2}^{\varepsilon }\otimes \sigma _{-k,1}^{\varepsilon }) \nabla F\right) -\tilde{R}\\  &\quad =\operatorname {div}\left( \overline{Q^{\varepsilon }_{\rho }}(0) \nabla F\right) -\tilde{R}, \end{aligned}$$

where we denoted by

$$\begin{aligned} \tilde{R}&=\sum _{\begin{array}{c} k\in \mathbb {Z}^{3,N}_0\\ k_3=0 \end{array}}\sigma _{k,1}^{\varepsilon }\cdot \nabla \left( F\cdot \nabla \sigma _{-k,2}^{\varepsilon }\right) +\left( \sigma _{-k,1}^{\varepsilon }\cdot \nabla F\right) \cdot \nabla \sigma _{k,2}^{\varepsilon }-(F\cdot \nabla \sigma _{-k,1}^{\varepsilon })\cdot \nabla \sigma _{k,2}^{\varepsilon }\\  &\quad +\sum _{\begin{array}{c} k\in \mathbb {Z}^{3,N}_0\\ k_3=0 \end{array}}\sigma _{k,2}^{\varepsilon }\cdot \nabla \left( F\cdot \nabla \sigma _{-k,1}^{\varepsilon }\right) +\left( \sigma _{-k,2}^{\varepsilon }\cdot \nabla F\right) \cdot \nabla \sigma _{k,1}^{\varepsilon }-(F\cdot \nabla \sigma _{-k,2}^{\varepsilon })\cdot \nabla \sigma _{k,1}^{\varepsilon }. \end{aligned}$$

Since $\overline{Q^{\varepsilon }_{\rho }}(0)=0$, we are left to show that

A.5 $$\begin{aligned} -\rho \tilde{R}=\Lambda ^{\varepsilon }_{\rho }F. \end{aligned}$$

First we observe that

$$\begin{aligned} \sigma _{k,1}^{\varepsilon }\cdot \nabla \left( F\cdot \nabla \sigma _{-k,2}^{\varepsilon }\right) +\sigma _{k,2}^{\varepsilon }\cdot \nabla \left( F\cdot \nabla \sigma _{-k,1}^{\varepsilon }\right)&=\left( \sigma _{k,1}^{\varepsilon }\cdot \nabla F\right) \cdot \nabla \sigma _{-k,2}^{\varepsilon }+\sigma _{k,1}^{\varepsilon }\cdot \nabla ^2 \sigma _{-k,2}^{\varepsilon } F\\  &\quad +\left( \sigma _{k,2}^{\varepsilon }\cdot \nabla F\right) \cdot \nabla \sigma _{-k,1}^{\varepsilon }+\sigma _{k,2}^{\varepsilon }\cdot \nabla ^2 \sigma _{-k,1}^{\varepsilon } F. \end{aligned}$$

Therefore

A.6 $$\begin{aligned} \tilde{R}&=\left( \sum _{\begin{array}{c} k\in \mathbb {Z}^{3,N}_0\\ k_3=0 \end{array}}\left( \sigma _{-k,1}^{\varepsilon }\cdot \nabla F\right) \cdot \nabla \sigma _{k,2}^{\varepsilon }+\left( \sigma _{-k,2}^{\varepsilon }\cdot \nabla F\right) \cdot \nabla \sigma _{k,1}^{\varepsilon }+\left( \sigma _{k,1}^{\varepsilon }\cdot \nabla F\right) \cdot \nabla \sigma _{-k,2}^{\varepsilon }+\left( \sigma _{k,2}^{\varepsilon }\cdot \nabla F\right) \cdot \nabla \sigma _{-k,1}^{\varepsilon }\right) \nonumber \\  &\quad + \left( \sum _{\begin{array}{c} k\in \mathbb {Z}^{3,N}_0\\ k_3=0 \end{array}}\sigma _{k,1}^{\varepsilon }\cdot \nabla ^2-(F\cdot \nabla \sigma _{-k,1}^{\varepsilon })\cdot \nabla \sigma _{k,2}^{\varepsilon } \sigma _{-k,2}^{\varepsilon } F+\sigma _{k,2}^{\varepsilon }\cdot \nabla ^2 \sigma _{-k,1}^{\varepsilon } F-(F\cdot \nabla \sigma _{-k,2}^{\varepsilon })\cdot \nabla \sigma _{k,1}^{\varepsilon }\right) \nonumber \\  &= \tilde{R}_1+\tilde{R}_2. \end{aligned}$$

$\tilde{R}_2=0$ arguing as above when showing that $R_2=0.$ We have for each *k* such that $k_3=0$

A.7 $$\begin{aligned}&\left( \sigma _{-k,1}^{\varepsilon }\cdot \nabla F\right) \cdot \nabla \sigma _{k,2}^{\varepsilon }=\left( \sigma _{k,1}^{\varepsilon }\cdot \nabla F\right) \cdot \nabla \sigma _{-k,2}^{\varepsilon },\quad \nonumber \\  &\left( \sigma _{-k,2}^{\varepsilon }\cdot \nabla F\right) \cdot \nabla \sigma _{k,1}^{\varepsilon }=\left( \sigma _{k,2}^{\varepsilon }\cdot \nabla F\right) \cdot \nabla \sigma _{-k,1}^{\varepsilon } \end{aligned}$$

due to our choice of the $\sigma _{k,j}^{\varepsilon }$. Therefore

$$\begin{aligned} \tilde{R}=2\sum _{\begin{array}{c} k\in \mathbb {Z}^{3,N}_0\\ k_3=0 \end{array}}\left( \sigma _{k,1}^{\varepsilon }\cdot \nabla F\right) \cdot \nabla \sigma _{-k,2}^{\varepsilon }+\left( \sigma _{k,2}^{\varepsilon }\cdot \nabla F\right) \cdot \nabla \sigma _{-k,1}^{\varepsilon }. \end{aligned}$$

Since, due to (2.15), we have for each $l\in \{1,2,3\}$

$$\begin{aligned} \partial _l \overline{Q^{\varepsilon }_{\rho }}(0)=2i\rho \sum _{\begin{array}{c} k\in \mathbb {Z}^{3,N}_0\\ k_3=0 \end{array}}\left( \theta _{k,1}^{\varepsilon }{\theta _{k,2}^{\varepsilon }}^* a_{k,1}\otimes a_{k,2}+\theta _{k,2}^{\varepsilon }{\theta _{k,1}^{\varepsilon }}^* a_{k,2}\otimes a_{k,1}\right) k_l, \end{aligned}$$

it follows that

A.8 $$\begin{aligned} -\rho \tilde{R}=-\sum _{l\in \{1,2\}} \partial _l \overline{Q^{\varepsilon }_{\rho }}(0)\cdot \nabla F_l. \end{aligned}$$

Relation (A.5) is a consequence of (A.6), (A.7), (A.8).

### Remark A.1

Due to Lemma 2.12, recalling the definition of $\mathcal {R}_{\varepsilon ,\gamma }$ in (2.19), it follows that if $F:\mathbb {T}^2\rightarrow \mathbb {R}^3$ we have

$$\begin{aligned} \Lambda _{\rho }^\varepsilon F&=-\sum _{l\in \{1,2\}}(0,0,\partial _l \overline{Q^{\varepsilon ,3,H}_{\rho }}(0)\cdot \nabla _H F_l)^t\\  &=2\rho \sum _{l\in \{1,2\}}\sum _{\begin{array}{c} k\in \mathbb {Z}^{3,N}_0\\ k_3=0 \end{array}}(0,0,\theta _{k,1}^{\varepsilon }\theta _{k,2}^{\varepsilon } k_l a_{k,1}\cdot \nabla _H F_l)^t\\  &=2\rho \sum _{l\in \{1,2\}}\sum _{\begin{array}{c} k\in \mathbb {Z}^{3,N}_0\\ k_3=0 \end{array}}(0,0,\theta _{k,1}^{\varepsilon }\theta _{k,2}^{\varepsilon }\operatorname {div}\left( k_l a_{k,1}F_l\right) )^t\\  &= -\left( 0,0,\operatorname {div}\left( \mathcal {R}_{\varepsilon ,\gamma } F_H\right) \right) ^t. \end{aligned}$$

More generally if $F:\mathbb {T}^3_{\varepsilon }\rightarrow \mathbb {R}^3$ we have

$$\begin{aligned} \Lambda ^{\varepsilon }_{\rho }F&=-\sum _{l\in \{1,2\}}(\partial _l \overline{Q^{\varepsilon ,H,3}_{\rho }}(0)\partial _3 F_l,\partial _l \overline{Q^{\varepsilon ,3,H}_{\rho }}(0)\cdot \nabla _H F_l)^t\\  &=-\left( \sum _{l\in \{1,2\}}\partial _3(\partial _l \overline{Q^{\varepsilon ,H,3}_{\rho }}(0) F_l),\operatorname {div}_H\left( \mathcal {R}_{\varepsilon ,\gamma } F_H\right) \right) ^t. \end{aligned}$$

## Some physical remarks

In this section we discuss some physical properties of our random vector field connected with the appearance of the alpha-term, in particular we show that the helicity of our vector field approach a constant value in the limit, despite the noise is weakly converging to zero. Let us compute the time average of the helicity of our random velocity field (in the following we will omit the dependence on time and space)

$$\begin{aligned} \mathcal {H}^{\varepsilon ,\gamma }:= \frac{1}{2T}\mathbb {E} \left[ W^\varepsilon (T,x)\cdot ({{\,\mathrm{\nabla }\,}}\times W^\varepsilon (T, x))\right] . \end{aligned}$$

As before we split our noise in mean and fluctuations along the thin direction $W^\varepsilon = \overline{W^\varepsilon }+ {W^\varepsilon }'$, and split the mean part in horizontal and vertical components $\overline{W^\varepsilon }= \overline{W^{\varepsilon ,H}} + \overline{W^{\varepsilon ,3}}e_3$. Observe that

$$\begin{aligned} {{\,\mathrm{\nabla }\,}}\times \overline{W^\varepsilon } = (-{{\,\mathrm{\nabla }\,}}^\perp \overline{W^{\varepsilon ,3}}, {{\,\mathrm{\nabla }\,}}^\perp \cdot \overline{W^{\varepsilon ,H}}), \end{aligned}$$

where with a small abuse of notation we have indicated with $\overline{W^{\varepsilon ,3}}$ the scalar quantity $\overline{W^\varepsilon }\cdot e_3$

$$\begin{aligned} \mathcal {H}^{\varepsilon ,\gamma } = \frac{1}{2T}\mathbb {E} \left[ -\overline{W^{\varepsilon ,H}} \cdot {{\,\mathrm{\nabla }\,}}^\perp \overline{W^{\varepsilon ,3}} + \overline{W^{\varepsilon ,3}}{{\,\mathrm{\nabla }\,}}^\perp \cdot \overline{W^{\varepsilon ,H}} + {W^\varepsilon }'\cdot ({{\,\mathrm{\nabla }\,}}\times \overline{W^\varepsilon }) + \overline{W^\varepsilon }\cdot ({{\,\mathrm{\nabla }\,}}\times {W^\varepsilon }') + {W^\varepsilon }'\cdot ({{\,\mathrm{\nabla }\,}}\times {W^\varepsilon }')\right] . \end{aligned}$$

Notice that the mixed terms are zero by the independence of the Brownian motions, thus we are left with the first two terms, and the last one. We start by the first two, we have, by our choices of the noise coefficients,

$$\begin{aligned} -\overline{W^{\varepsilon ,H}} \cdot {{\,\mathrm{\nabla }\,}}^\perp \overline{W^{\varepsilon ,3}}&= -\sum _{\begin{array}{c} k, k'\in \mathbb {Z}^2_0,\\ N\le |k|,|k'|\le 2N \end{array}} \theta ^{\varepsilon }_{k,1}\theta ^{\varepsilon }_{k',2} a_k^1\cdot (i{k'}^\perp )e^{i(k+k')\cdot x} W^{k,1}W^{k', 2}\\&= \frac{1}{C_{1,H}C_{2,H}}\sum _{\begin{array}{c} k, k'\in \mathbb {Z}^2_0,\\ N\le |k|,|k'|\le 2N \end{array}} \frac{k^\perp \cdot (k')^\perp }{|k|^{2}|k'|^{\gamma /2}} e^{i(k+k')\cdot x}W^{k,1}W^{k', 2}. \end{aligned}$$

Taking the expected value and remembering that $\mathbb {E} \left[ W_T^{k,1}W_T^{k',2}\right] = 2T\rho \delta _{k+k'}$ we get

$$\begin{aligned} \mathbb {E} \left[ -\overline{W^{\varepsilon ,H}} \cdot {{\,\mathrm{\nabla }\,}}^\perp \overline{W^{\varepsilon ,3}}\right] = -\frac{2\rho T}{C_{1,H}C_{2,H}} \sum _{\begin{array}{c} k\in \mathbb {Z}^2_0 \\ N\le |k|\le 2N \end{array}} \frac{1}{|k|^{\gamma /2}}. \end{aligned}$$

Analogously, we have

$$\begin{aligned} \overline{W^{\varepsilon ,3}} ({{\,\mathrm{\nabla }\,}}^\perp \cdot \overline{W^{\varepsilon , H}})&= -\frac{1}{C_{1,H}C_{2,H}}\sum _{\begin{array}{c} k, k'\in \mathbb {Z}^2_0,\\ N\le |k|,|k'|\le 2N \end{array}} \frac{1}{|k|^{\gamma /2}} e^{i(k+k')\cdot x}W^{k,2}W^{k', 1}. \end{aligned}$$

This implies

$$\begin{aligned} \mathbb {E} \left[ \overline{W^{\varepsilon ,3}} ({{\,\mathrm{\nabla }\,}}^\perp \cdot \overline{W^{\varepsilon , H}})\right] = - \frac{2\rho T}{C_{1,H}C_{2,H}}\sum _{\begin{array}{c} k\in \mathbb {Z}^2_0 \\ N\le |k|\le 2N \end{array}} \frac{1}{|k|^{\gamma /2}}. \end{aligned}$$

Finally for the last term

$$\begin{aligned} {W^\varepsilon }'\cdot ({{\,\mathrm{\nabla }\,}}\times {W^\varepsilon }')= \sum _{\begin{array}{c} k, k'\in \mathbb {Z}^{3, \varepsilon }_0,\\ k_3, k'_3\ne 0 \\ N\le |k_H|,|k_H'|\le 2N\\ i,j\in \{1,2\} \end{array}}\theta ^{\varepsilon }_{i,k}\theta ^{\varepsilon }_{k,k'} a_k^i\cdot (ik'\times a_{k'}^j) e^{i(k+k')\cdot x}W^{k,i}W^{k', j}. \end{aligned}$$

Taking expectation, we see that only the terms with $k^{\prime }=-k$ and i=j survive but then, $$a_k^i\cdot (k\times a_{-k}^i)= a_k^i\cdot (k\times a_{k}^i)= 0$$. In the end $$\mathcal {H}^{\varepsilon ,\gamma }$$ does not vanish in the limit if and only if γ=4. Indeed, we have

B.1 $$\begin{aligned} \mathcal {H}^{\varepsilon ,\gamma }=-\frac{2\rho \zeta ^{N}_{H,\gamma /2}}{N^{\gamma /2-2}C_{1,H}C_{2,H}}\rightarrow \mathcal {H}^{\gamma }={\left\{ \begin{array}{ll} -\frac{4\pi \rho \log 2}{C_{1,H}C_{2,H}} & \text {if } \gamma =4\\ 0 & \text {if } \gamma >4. \end{array}\right. } \end{aligned}$$

Due to (B.1) it follows that

B.2 $$\begin{aligned} \mathcal {R}_{\varepsilon ,\gamma }=-\frac{\mathcal {H}^{\varepsilon ,\gamma }}{2}\begin{bmatrix} 0 &  -1\\ 1 &  0 \end{bmatrix},\ \mathcal {R}_{\gamma }=-\frac{\mathcal {H}^{\gamma }}{2}\begin{bmatrix} 0 &  -1\\ 1 &  0 \end{bmatrix}. \end{aligned}$$

Finally, due to (B.2), denoting by

$$\begin{aligned} \overline{B^{\gamma }_t}=(-\nabla ^{\perp } \overline{A^{3,\gamma }_t}, \overline{B^{3,\gamma }_t})^t, \quad \mathcal {A}^{\gamma }=-\frac{\mathcal {H}^{\gamma }}{2}\begin{bmatrix} 1&  0 &  0\\ 0 & 1 &  0\\ 0 &  0 &  0 \end{bmatrix}, \end{aligned}$$

our limit equations (2.30), (2.31) read as

B.3 $$\begin{aligned} {\left\{ \begin{array}{ll} \partial _t \overline{B^{\gamma }_t} & =(\eta +\eta _T)\Delta \overline{B^{\gamma }_t}+\nabla \times (\mathcal {A}^{\gamma }\overline{B^{\gamma }_t})\quad x\in \mathbb {T}^2,\ t\in (0,T)\\ \operatorname {div}\overline{B^{\gamma }_t}&  =0\\ \overline{B^{\gamma }_t}|_{t=0}& = \overline{B}_0. \end{array}\right. } \end{aligned}$$
